# Low-Temperature Meltable Elastomers Based on Linear Polydimethylsiloxane Chains Alpha, Omega-Terminated with Mesogenic Groups as Physical Crosslinkers: A Passive Smart Material with Potential as Viscoelastic Coupling. Part I: Synthesis and Phase Behavior

**DOI:** 10.3390/polym12112476

**Published:** 2020-10-25

**Authors:** Sabina Horodecka, Adam Strachota, Beata Mossety-Leszczak, Beata Strachota, Miroslav Šlouf, Alexander Zhigunov, Michaela Vyroubalová, Dana Kaňková, Miloš Netopilík, Zuzana Walterová

**Affiliations:** 1 Institute of Macromolecular Chemistry, Czech Academy of Sciences, Heyrovskeho nam. 2, CZ-162 06 Praha, Czech Republic; horodecka@imc.cas.cz (S.H.); beata@imc.cas.cz (B.S.); slouf@imc.cas.cz (M.Š.); zhigunov@imc.cas.cz (A.Z.); vyroubalova@imc.cas.cz (M.V.); kankova@imc.cas.cz (D.K.); netopilik@imc.cas.cz (M.N.); walterova@imc.cas.cz (Z.W.); 2 Faculty of Science, Charles University, Albertov 6, CZ-128 00 Praha 2, Czech Republic; 3 Faculty of Chemistry, Rzeszow University of Technology, al. Powstancow Warszawy 6, PL-35-959 Rzeszow, Poland; mossety@prz.edu.pl

**Keywords:** reversible networks, self-assembly, self-healing, liquid crystals, smart materials

## Abstract

Physically crosslinked low-temperature elastomers were prepared based on linear polydimethylsiloxane (PDMS) elastic chains terminated on both ends with mesogenic building blocks (LC) of azobenzene type. They are generally (and also structurally) highly different from the well-studied LC polymer networks (light-sensitive actuators). The LC units also make up only a small volume fraction in our materials and they do not generate elastic energy upon irradiation, but they act as physical crosslinkers with thermotropic properties. Our elastomers lack permanent chemical crosslinks—their structure is fully linear. The aggregation of the relatively rare, small, and spatially separated terminal LC units nevertheless proved to be a considerably strong crosslinking mechanism. The most attractive product displays a rubber plateau extending over 100 °C, melts near 8 °C, and is soluble in organic solvents. The self-assembly (via LC aggregation) of the copolymer molecules leads to a distinctly lamellar structure indicated by X-ray diffraction (XRD). This structure persists also in melt (polarized light microscopy, XRD), where 1–2 thermotropic transitions occur. The interesting effects of the properties of this lamellar structure on viscoelastic and rheological properties in the rubbery and in the melt state are discussed in a follow-up paper (“Part II”). The copolymers might be of interest as passive smart materials, especially as temperature-controlled elastic/viscoelastic mechanical coupling. Our study focuses on the comparison of physical properties and structure–property relationships in three systems with elastic PDMS segments of different length (8.6, 16.3, and 64.4 repeat units).

## 1. Introduction

This work is dedicated to low-temperature reversible elastomers which melt somewhat below room temperature and which are based on linear polydimethylsiloxane (PDMS) of different chain lengths, terminated with liquid crystalline (LC) units in the α- and ω- position. The LC end-groups act as physical crosslinkers in this special variety of polysiloxane/LC copolymers.

Generally, polysiloxane copolymers with mesogenic (LC) building blocks are compounds which can offer fascinating material properties, due to the combination of the highly flexible and hydrophobic polysiloxane with the phase behavior of the mesogenic (LC) units [1,2]. These materials have attracted deserved research interest since Finkelmann’s pioneering works in the 1980s [3,4,5,6,7]. Subsequently, numerous studies were dedicated to liquid crystalline siloxane polymers (LCPs) with mesogens as side chains (comb-like LCPs; see e.g., [1,8], (Volume 3: pp. 121–302) and [9,10,11]), whereas main-chain siloxane LC copolymers, to which the studied materials also belong as a special case, were studied only in a few works in total [12,13,14,15,16,17].

All the abovementioned polysiloxane LCPs studied in the literature were very rich in the LC component, which made up a dominant volume fraction, and the behavior of the usually more or less fixed (via chemical crosslinking) mesogens was responsible for practically all the material properties. In contrast to that, in the presented work, the mesogen makes up a relatively small volume fraction of most of the copolymers, the elastic properties originate from the PDMS component, while the melting, the gelation, and the mechanical disconnection and reconnection of the physical crosslinks are controlled by the phase behavior of the mesogen.

Synthesis routes to polysiloxane LCPs most frequently employed the hydrosilylation reaction, as is the case in this work, namely by connecting vinyl-functional mesogens with hydrido-functional (Si–H) PDMS. All the abovementioned main-chain siloxane LCPs were prepared via this route, while in the case of the more frequently investigated comb-like polysiloxane LCPs, alkyne/SiH addition [11], thiol-ene addition [18], or the Huisgen “click reaction” [19] are employed as alternative synthesis routes.

From the application point of view, polysiloxane LCPs have been investigated as electro-optic [20], light-emitting [21], gas separation (membrane-) [22,23], and chromatography materials [24], as well as actuators (see e.g., [16,25,26,27]). In the latter application, which gained by far the most research attention, especially in recent years, they are often referred to as “liquid-crystalline elastomers” (LCEs). The relatively rarely studied main-chain polysiloxane LCPs, to which the materials studied in this work belong, were investigated mainly for their thermotropic phase behavior (polyester-*co*-PDMS systems) [12,13,14,15], for their thermomechanical properties [15], as thermosensitive actuators (mechanism via smectic ↔ isotropic transition of the polyester LC units) [16], or as photoresponsive optical material (azo units’ cis/trans isomerization) [17]. In contrast to that, in the present work, the polysiloxane LCPs were studied as potential structural smart materials, namely as thermoresponsive viscoelastic coupling, with marked phase transitions also in the oily (melt) state.

The physical crosslinking in the copolymers which are studied in this work is based on the aggregation and microphase separation of the terminal mesogenic (LC) units, and thus, results in an organic–organic nanocomposite morphology. This morphology plays a key role in the copolymers’ material properties, especially in the efficiency of the crosslinking and in the thermomechanical properties. In their previous work [28,29], the authors studied in detail the nano-aggregation behavior of rigid inorganic polyhedral oligomeric silsesquioxane (POSS) building blocks in epoxy–POSS hybrid copolymers. The nanodomains of POSS (physical crosslinker and simultaneously, a hard nanofiller) caused a very strong mechanical reinforcement, as well as thermal stabilization. The organic substituents which covered the POSS surface were found to control its aggregation; substituents with the strongest crystallization tendency were the most efficient in achieving physical crosslinking and mechanical reinforcement by POSS. Similar results concerning aggregation behavior were also observed for the heavier homologue of POSS, the stannoxane dodecamer cages (“Sn-POSS”), which additionally introduced the specific chemical reactivity of the filler phase, namely matrix repair reactions under oxidizing conditions [30,31].

The authors’ experience with physical crosslinking via aggregation (nanocrystallization) of the abovementioned POSS nano-building blocks, as well as the previous study on liquid crystalline epoxy copolymer networks that were able to be orientated [32], inspired the present work. The authors also recently studied [33] a PDMS–LC copolymer which is structurally highly different from the presently investigated materials. In the mentioned study, a linear PDMS chain was tethered with spatially separated quartets of pendant LC groups. The study in [33] was the authors’ first foray into the broader topic of the present work. In this present work, however, the same LC groups are tested in a completely different copolymer architecture.

Related to the presented work also is the topic of physical (non-covalent) crosslinking of PDMS. Several interesting approaches were studied in the literature, namely crosslinking via strongly hydrogen-bridging groups attached to PDMS macromolecules (or oligomers), like urea [34], ureidopyrimidinone [35], benzene-1,3,5-tricarboxamide (BTA) derivatives [36], or cyclodextrin (in α,ω-positions) [37]. Other studied non-covalent crosslink-forming groups attached to PDMS included π-stacking moieties [38,39,40]. Combinations of Lewis acidic and Lewis basic functional groups, both attached to PDMS molecules (cyclic boronic esters and amines) [41] or combinations of mildly bonding ligand groups on PDMS and free metal cations [42] already could be considered as PDMS crosslinking via reversible covalent bonds, rather than physical crosslinking. Depending on the crosslink strength, the described products, which were often referred to as “thermoplastic elastomers”, were processable by the combination of heat and solvent, by melting or dissolution alone, or even by cold pressing. In contrast to the above, the PDMS copolymers studied in the presented work are crosslinked by a rather mild variety of non-covalent interactions. The aggregation of the “active” structural units is driven by their crystallization tendency, with no hydrogen bridging, no strong π-stacking, and no strong electrostatic attraction.

The aim of the presented work was to prepare physically crosslinked meltable elastomers based on linear polydimethylsiloxane chains (PDMS macromonomer) α,ω-terminated with mesogenic (LC) building blocks. The nanophase separation and crystallization of the latter was favored by PDMS/mesogen incompatibility and was expected to efficiently crosslink the elastic PDMS chains. Thus, the LC building blocks were expected to play a somewhat similar role (non-covalent crosslinker) like the inorganic POSS units studied in the authors’ early work. Moreover, due to their specific properties, the LC units could cause interesting thermotropic behavior of the physical crosslinks in the studied materials. This would be of interest for smart applications, such as viscoelastic coupling materials, behaving as oils with greatly varying temperature-controlled viscoelasticity near room temperature, and as rubbers at lower temperatures. The detailed aims of this work included the elucidation of the structure–property relationships in the PDMS–LC elastomers, especially the efficiency as a physical crosslinker of the relatively small mesogen unit, and the effect of the length of the elastic PDMS chains. While the presented work focuses on the synthesis and on the comprehensive characterization of the thermotropic phase behavior, including simple mechanical properties, a follow-up paper [43] (“Part II”) is dedicated to the study of the complex viscoelastic and rheological properties of the copolymers in the molten as well as in the rubbery state.

## 2. Experimental Section

### 2.1. Materials

#### 2.1.1. Commercial Chemicals

The hydride-α,ω-terminated polydimethylsiloxanes—DMS H03 (*M*_n_ = 623.9 g/mol), DMS H11 (*M*_n_ = 1196.5 g/mol), and DMS H21 (*M*_n_ = 4764 g/mol) were purchased from Gelest, Morrisville, PA, USA. Chloroform (solvent) was purchased from Sigma-Aldrich, St. Louis, MO, USA, while a 2% solution of Karstedt’s catalyst was purchased from Merck (Darmstadt, Germany). All the commercial products were used as received. Prior to use, however, the equivalent molecular masses per SiH group of the DMS H03, DMS H11, and DMS H21 were more precisely determined by ^1^H-NMR. The so-obtained equivalent molecular masses (per Si–H function: DMS H03—311.9325 g/mol; DMS H11—598.241 g/mol; DMS H21—2382.2 g/mol) were then used for calculations of the amounts of reactants in the syntheses.

#### 2.1.2. Synthesis of the Liquid Crystalline Mesogen named “BAFKU”

The synthesis of the azo-type mesogen BAFKU (*M*_w_ = 420.587 g/mol) was described in detail in a previous work by some of the authors of this paper [44].

#### 2.1.3. Preparation of the Main Chain Liquid Crystalline Copolymers Based on PDMS (DMS H03, DMS H11, or DMS H21) and LC Mesogen BAFKU

Appropriate quantities of the BAFKU mesogen and of PDMS (see Table 1) were placed into a small vessel equipped with a magnetic stirring bar. In total, 2 mL of chloroform was added, and after flushing the reactants with argon, they were dissolved by brief stirring at 60 °C. Subsequently, Karstedt’s catalyst (8.7 mg of a 2 wt.% solution, 0.0228 mmol Pt) was added and the solution was stirred at 60 °C for 5 min. Thereafter, the reaction mixture was cooled down to room temperature and the solvent was removed under reduced pressure. The so-obtained copolymer was further dried under vacuum (10 mbar) for 30 min at 90 °C (molten state). The product was then cooled down to room temperature and stored. The trace amounts of the Karstedt catalyst (between 0.008 and 0.02 wt.%) were tolerated as non-problematic impurity in the case of standard syntheses (it was possible to remove them by adsorption on silica or on activated carbon at the cost of reduced yield).

The volume fractions of the mesogen BAFKU were calculated using Equation (1) under consideration of the densities (ρ(BAFKU) = 1.05 g/cm^3^, ρ(H03) = 0.90 g/cm^3^, ρ(H11) = 0.93 g/cm^3^, and ρ(H21) = 0.97 g/cm^3^) and of the stoichiometric synthesis amounts (see above in Table 1) of the individual components.

(1) $$\mathrm{volume}\;\mathrm{percent}\;\left(\mathrm{Vol}\%\right)=\;\frac{\frac{m\left(\mathrm{component}\right)}{\rho \left(\mathrm{component}\right)}}{\Sigma \left(\frac{m\left(i\right)}{\rho \left(i\right)}\right)}\times 100\%$$

Yield: 

H03–BAFKU_2_: 0.8715 g (1.19 mmol), 100% of theory (one-pot synthesis).

H11–BAFKU_2_: 1.2069 g (1.19 mmol), 100% of theory (one-pot synthesis).

H21–BAFKU_2_: 3.311 g (1.19 mmol), 100% of theory (one-pot synthesis).

Characterization by ^1^H-NMR (proton nuclear magnetic resonance), in CDCl_3_, δ (ppm) (see also spectra in SI Figures S7 and S8 in the Supplementary Materials):

H03–BAFKU_2_: 7.94 (4H, d, arom.), 7.84 (4H, d, arom.), 7.33 (4H, d, arom.), 7.26 (4H, d, arom.), 2.69 (4H, m, CH_2_), 2.58 (4H, m, CH_2_), 1.75 (4H, m, CH_2_), 1.65 (4H, m, CH_2_), 1.55 (s, H_2_O: moisture in CDCl_3_), 1.29 (32H, m, CH_2_), 0.94 (6H, m, CH_2_), 0.53 (4H, m, CH_2_), 0.06 (52H, s, CH_3_ on Si, plus two CH_2_ groups on Si, the latter two originating from BAFKU).

H11–BAFKU_2_: 7.92 (4H, d, arom.), 7.82 (4H, d, arom.), 7.30 (4H, d, arom.), 7.22 (4H, d, arom.), 2.69 (4H, m, CH_2_), 2.58 (4H, m, CH_2_), 1.77 (4H, m, CH_2_), 1.63 (4H, m, CH_2_), 1.55 (s, H_2_O: moisture in CDCl_3_), 1.29 (32H, m, CH_2_), 0.91 (6H, m, CH_3_), 0.53 (4H, m, CH_2_), 0.07 (96H, s, CH_3_ on Si, plus two CH_2_ groups on Si, the latter two originating from BAFKU).

H21–BAFKU_2_: 7.92 (4H, d, arom.), 7.84 (4H, d, arom.), 7.33 (4H, d, arom.), 7.24 (4H, d, arom.), 2.69 (4H, m, CH_2_), 2.58 (4H, m, CH_2_), 1.77 (4H, m, CH_2_), 1.65 (4H, m, CH_2_), 1.29 (32H, m, CH_2_), 0.94 (6H, m, CH_3_), 0.52 (4H, m, CH_2_), 0.06 (392H, s, CH_3_ on Si, plus two CH_2_ groups on Si, the latter two originating from BAFKU).

For comparison (see also spectra in SI Figures S4–S6):

Neat BAFKU: 7.90 (2H, d, arom.), 7.82 (2H, d, arom.), 7.31 (2H, d, arom.), 7.22 (2H, d, arom.), 5.79 (1H, m, CH), 4.94 (2H, m, CH_2_), 2.59 (2H, m, CH_2_), 2.56 (2H, m, CH_2_), 2.03 (2H, m, allyl-type CH_2_), 1.65 (2H, m, CH_2_), 1.60 (2H, m, CH_2_), 1.31 (12H, m, CH_2_), 0.92 (3H, m, CH_3_).

Neat DMS H03: 4.70 (2H, m, SiH), 0.18 (12H, s, CH_3_ on Si in terminal units), 0.06 (40H, s, CH_3_ on Si in internal repeat units).

Neat DMS H11: 4.71 (2H, m, SiH), 0.17 (12H, s, CH_3_), 0.08 (84H, s, CH_3_).

Neat DMS H21: 4.69 (2H, m, SiH), 0.13 (12H, s, CH_3_), 0.06 (380H, s, CH_3_).

Check of quantitative conversion:

FTIR (Fourier-transform infrared spectroscopy): Complete disappearance of the peak of the Si–H bond stretching at about 2120 cm^−1^ (see SI Figures S1–S3).

^1^H-NMR: Complete disappearance of the peaks near 4.7 ppm (Si–H) and of the multiplets near 5.79 and 4.94 ppm (vinyl groups of LC mesogen), see spectra in SI Figures S7 and S8.

### 2.2. Characterization of the PDMS–BAFKU_2_ Copolymers

#### 2.2.1. Chemical Microstructure

^1^H-NMR (proton nuclear magnetic resonance): The chemical structure (including the molecular masses, via end-group determination) of the synthesized PDMS–BAFKU_2_ copolymers was verified using ^1^H-NMR spectroscopy. The same method was also used for verifying the purity of the mesogen “BAFKU” and for determining the equivalent molecular masses per SiH of the α,ω-hydrido-functional polydimethylsiloxanes DMS H03, DMS H11, and DMS H21. The spectra were recorded on an Avance DPX 300 spectrometer (from Bruker, Karlsruhe, Germany) at 300 MHz. CDCl_3_-d_1_ was used as solvent for all experiments. Tetramethylsilane (TMS; *δ* = 0 ppm) was employed as internal standard.

FTIR (Fourier-transform infrared spectroscopy): The completion of hydrosilylation reactions between SiH-functional polydimethyl-siloxanes and the monovinyl-functional mesogen was verified by means of FTIR spectroscopy. The spectra were recorded in the attenuated total reflection (ATR) mode using a Nicolet 8700 spectrometer (from Thermo Scientific, Madison, WI, USA). The ATR spectra were recorded using a Golden Gate™ heatable Diamond ATR Top-Plate (MKII single reflection ATR system, from Specac, Orprington, Greater London, UK). 

Molecular masses via SEC (size exclusion chromatography; or gel permeation chromatography “GPC”): The molecular masses of the prepared copolymers and of their precursors were determined by SEC. For this purpose, we used a Deltachrom pump with computer-controlled piston movement (from Watrex Praha, s.r.o., Praha, Czech Republic), the autosampler MIDAS (from Spark, Emmen, Holland), two columns “PLgel 5 μm MIXED-B” (10 μm particles; from Polymer Laboratories, now Agilent Technologies, Santa Clara, CA, USA), which according to the manufacturer, separate in the molecular mass range of approximately 10^2^ ≤ *M* ≤ 1 × 10^6^. Evaporative light scattering detector (ELSD) PL ELS 1000 (from Polymer Laboratories, Church Stretton, Shropshire, UK, laser wavelength: 658 nm) and a UV–vis (refraction index) DeltaChrom UVD 200 detector (from Watrex Praha, s.r.o., Praha, Czech Republic) with a flow-cell volume of 8 μL, operating at wavelength *λ* = 264 nm, were the detectors in the order of flow. The data were collected into the Clarity software (from DataApex Ltd., Praha, Czech Republic), which communicated with the detectors using a U-PAD2 USB acquisition device (also from DataApex). The mobile phase was tetrahydrofuran (Thermo Fisher Scientific, Waltham, MA, USA) at 25 °C (controlled ambient temperature), used as received. The concentration of measured solutions was 5 mg/mL. Polystyrene standards obtained from Polymer Standards Services (Mainz, Germany) were used for calibration.

Molecular masses via MALDI-TOF (matrix-assisted laser desorption ionization/time of flight) mass spectroscopy: 

The samples were prepared by the “dried droplet method”: THF (tetrahydrofuran, ≥ 99.9%, Sigma-Aldrich, St. Louis, MO, USA) solutions of the analyzed polymer (10 mg mL^−1^), of the matrix DCTB (trans-2-[3-(4-t-butyl-phenyl)-2-methyl-2-propenyli-dene]malo-nitrile, Sigma-Aldrich, 10 mg mL^−1^), and of the cationization agent sodium trifluoroacetate (CF_3_COONa; Sigma-Aldrich, 10 mg mL^−1^) were mixed in the volume ratio 4:20:1. Then, 1 μL of the mixture (the “droplet”) was deposited on the ground-steel target plate, and dried at ambient atmosphere.

The MALDI-TOF mass spectra were acquired with an UltrafleXtreme apparatus (Bruker Daltonics, Bremen, Germany) in the positive ion reflectron and linear mode. Each spectrum was the sum of 25,000 shots with a DPSS (diode-pumped solid-state) Nd: YAG laser (neodymium-doped yttrium-aluminum garnet laser; 355 nm, 2000 Hz). Delayed extraction and external calibration were used.

#### 2.2.2. Thermo-Mechanical and Thermal Properties

DMTA (dynamic-mechanical thermal analysis): The advanced multi-functional rheometer ARES-G2 (from TA Instruments, New Castle, DE, USA—part of Waters, Milford, MA, USA) was used for characterizing the thermomechanical properties of the prepared copolymers. 

Typical combined tests, which covered the glassy state, the glass transition, the rubbery, as well as the melting region, were carried out using small parallel plate geometry (exchangeable stainless-steel plates, diameter: 6.1 mm). An oscillatory shear deformation at the constant frequency of 1 Hz and at a small strain amplitude (0.001% to 4%, adjusted by auto-strain) was applied. In the first scan, which was a cooling one, the samples were cooled down from the molten state at the rate of 3 °C/min, down to the final low temperature (−135 °C in the standard case). Subsequently, in the second scan, the heating one, the samples were heated at the same rate up to the final high temperature (40 °C in the standard case). For both scans, the temperature dependences of the storage and of the loss shear modulus were recorded, as well as of the loss factor (*G*’, *G*”, and tan(δ), respectively).

Differential scanning calorimetry (DSC): The nature of the transitions observed in the copolymers in the DMTA tests was further investigated by DSC. The experiments were performed on a DSC Q2000 instrument from TA Instruments (New Castle, DE, USA) under a nitrogen atmosphere. The temperature range was from −90 to 100 °C. The heating and cooling rate was always 10 °C/min. The samples were first cooled down to −90 °C, after which they were subjected to the first heating scan from −90 to 80 °C (with final temperature corresponding to the isotropic state of the polymer melt), followed by the first cooling scan (80 to −90 °C) and the second heating scan.

#### 2.2.3. Long-Range-Structure and Crystallinity Characterization

SAXS and XRD (small-angle X-ray scattering and X-ray diffraction): X-ray scattering experiments were performed using a MolMet pinhole camera from Rigaku, Tokyo, Japan, modified by SAXSLAB/Xenocs (headquarters: Grenoble, France) which was attached to a microfocused X-ray beam generator MicroMax 003 from Rigaku, operating at 50 kV and 0.6 mA (30 W). The camera was equipped with a vacuum version of the Pilatus 300K detector (from DECTRIS Ltd., Baden-Daettwil, Aargau, Switzerland). Variable detector positioning that covers the *q* range of 0.004–3.6 Å^−1^ was chosen. The scattering vector, *q*, is defined as: *q* = (4π/*λ*)sinθ, where *λ* = 1.54 Å is the wavelength of the installed source and 2θ is the scattering angle. The calibration of primary beam position and sample-to-detector distances was performed using Ag behenate and the Si powder sample. Homemade software based on the PyFAI Python library [45] was used for data reduction.

#### 2.2.4. Polarized Light Microscopy (PLM) Observation of Phase Transitions

Polarized light microscopy (PLM) [46,47]: In order to visualize the phase transitions in the studied copolymers, polarized light microscopy was employed. PLM micrographs were recorded with a Nikon Eclipse 80i (from Nikon, Shinagawa, Tokyo, Japan) microscope equipped with a ProgRes CT3 digital camera (Jenoptik, Jena, Germany) and THMS 600 heating stage (from Linkam Scientific Instruments Ltd. Tadworth, Surrey, UK).

Thin specimens for PLM (thickness < 10 μm) were prepared by compression molding on a heating bar (temperature of the bar 120 °C, compression between two glasses, load 5 kg for 10 min). The best PLM micrographs were obtained for the thinnest polymer films after slight shear deformation, which was induced by manual shift of the two glasses used for compression molding. 

All PLM experiments started at room temperature. The samples were then gradually heated with the rate of 1 °C/min up to a final temperature, where the melt was fully isotropic. After a short pause at the maximum temperature (5 min), the sample was cooled down to room temperature using the same rate.

The intensity of illumination was kept constant and PLM micrographs were recorded during the whole process. The intensity of polarized light was evaluated from the image analysis of the individual micrographs as the mean intensity of all pixels within a given micrograph (using the software NIS Elements 4.0 from Laboratory Imaging s.r.o., Praha, Czech Republic; morphological descriptor MeanIntensity). As the final output, the normalized MeanIntensity as a function of temperature was plotted. It has been demonstrated in previous studies by some of the authors of this paper [46,47] that the polarized light intensity recorded in this way is proportional to the amount of crystalline/anisotropic structures in the investigated specimen.

## 3. Results and Discussion

### 3.1. Synthesis

**Scheme 1:**
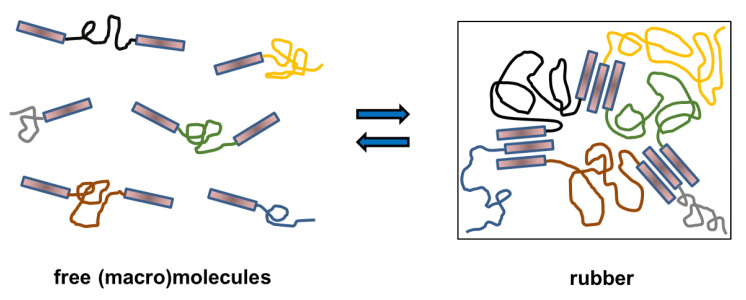
Symbolic representation of the linear PDMS–BAFKU_2_ copolymers and of their physical crosslinking via aggregation of the BAFKU units.

**Scheme 2:**
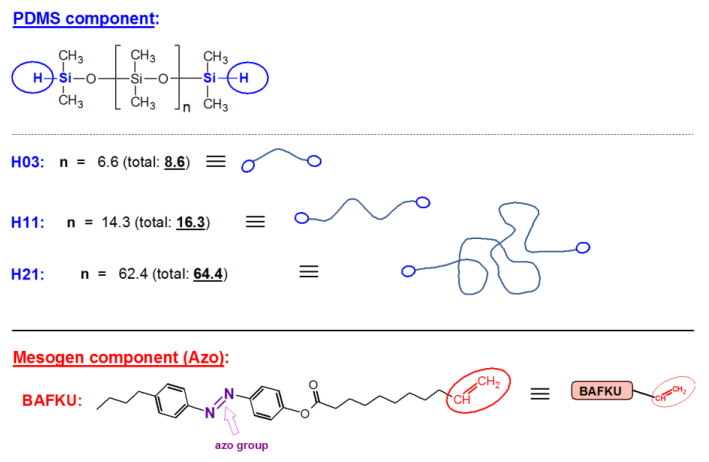
Components used to synthesize the studied copolymers: (**top**): general structure of the PDMS precursors with Si–H functions in α and ω-position, as well as the representation of typical molecules of the “hydrido-polysiloxanes” DMS H03, DMS H11, and DMS H21; (**bottom**): the mono-vinyl-functional mesogen BAFKU.

Several copolymers consisting of polydimethylsiloxane (PDMS) of different chain length, α,ω-terminated with mesogenic units of azobenzene-type (“BAFKU”), were synthesized and studied as potential passive smart materials. The assembly of these medium/short copolymer molecules to reversible (meltable) elastomeric networks is shown in Scheme 1. The structure of the constituent components of the prepared low-temperature reversible rubbers is shown in Scheme 2; they were chosen in order to combine properties of PDMS, such as polymer backbone flexibility and a low glass transition temperature (*T*_g_), with the crystallization tendency of the liquid crystalline BAFKU units, which had to act as thermotropic physical crosslinks, via BAFKU–BAFKU aggregation, as shown in Scheme 1. α,ω-SiH-terminated PDMS of three different lengths was employed: a 8.6-mer (on average, “H03”), a 16.3-mer (“H11”), and a 64.4-mer (“H21”). A potentially important feature of the prepared copolymers is the azo unit in their mesogenic building blocks. This unit, which normally is *trans*-configured, can be reversibly switched between *cis*- and *trans*- configurations by UV irradiation. In this way, the thermotropic properties of the physical crosslinks potentially could also be photo-switched between those of isomeric states. Photosensitivity was not studied in this already extensive work, however.

A key feature was the desired lack of miscibility (in the absence of solvent) of both components of the studied copolymers, which was achieved due to the specific properties of PDMS. This immiscibility favors nanophase separation in the copolymers via the formation of BAFKU aggregates, and hence, an easy formation of the physical network shown in Scheme 1. Additionally, the mesogen BAFKU introduces further interesting properties like the thermotropic behavior of the crosslinks.

**Scheme 3:**
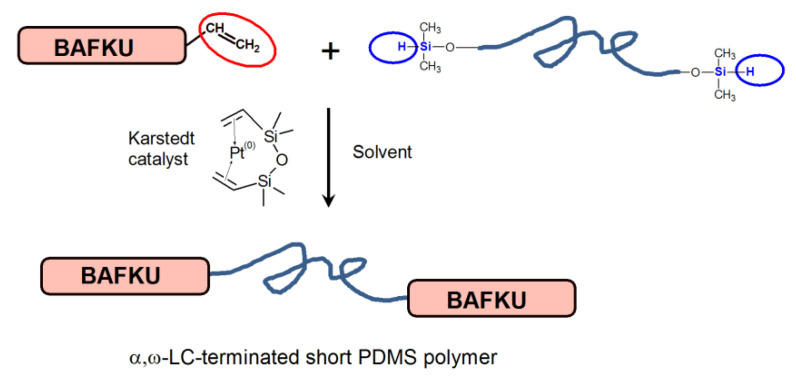
Synthesis of PDMS–BAFKU_2_ copolymers via hydrosilylation catalyzed by Karstedt’s catalyst.

SiH–PDMS and BAFKU were coupled via the hydrosilylation reaction (Scheme 3), which was considered to be practically a “click reaction”. All the syntheses were carried out in chloroform at 60 °C and catalyzed by Karstedt’s catalyst (also shown in Scheme 3). Prior to the syntheses, highly precise equivalent molecular masses per SiH functional group were determined for all the PDMS precursors by means of proton nuclear magnetic resonance (^1^H-NMR) analysis (see SI Table S1 in the Supplementary Materials), as the copolymers’ components had to be coupled in precisely stoichiometric ratios.

**Scheme 4:**
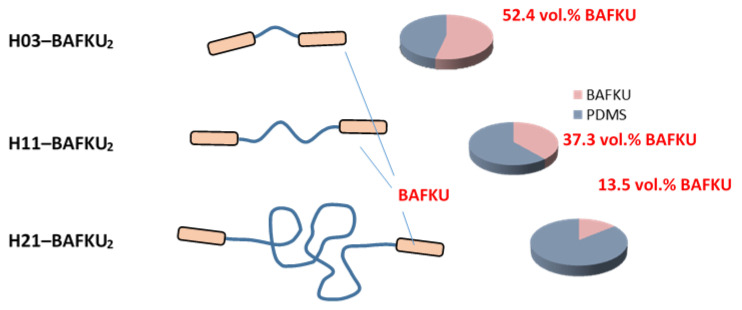
(**Left**): Representation of the prepared PDMS–BAFKU_2_ copolymers: H03–BAFKU_2_, H11–BAFKU_2_, and H21–BAFKU_2_, highlighting elastic chain length between the BAFKU mesogens attached at both ends; (**Right**): diagrams of volume fractions of the BAFKU mesogen units in each copolymer.

The structure of the obtained products with approximately realistic proportional sizes of the elastic chains and mesogens is shown in Scheme 4 further below. The latter scheme also shows diagrams with calculated volume fractions of PDMS and BAFKU in each copolymer. The volume fractions of BAFKU (see also Table 1) were calculated under consideration of the densities and of the stoichiometric synthesis amounts (see Table 1) of the individual components (details of the calculation are given in the Supplementary Materials).

General properties of the copolymers: All the obtained products were oily liquids at standard room temperature (25 °C), with melting points at 22 °C (H03–BAFKU_2_), 8 °C (H11–BAFKU_2_) and −15 °C (H21–BAFKU_2_). The respective freezing points are: 5, 0 and −20 °C. The copolymers can be dissolved in some common organic solvents like chloroform, tetrahydrofuran or toluene. In contrast to the liquid but colorless (and very low-temperature freezing) PDMS precursors, the copolymers display a dark orange color, like the molten neat BAFKU mesogen.

The efficiency of the hydrosilylation reaction was of great importance for accurate characterization of the copolymers’ material properties. A quantitative (100%) conversion was proven in all three reactant pairs by means of Fourier-transform infrared (FTIR) and ^1^H-NMR spectroscopy, namely as the disappearance of the characteristic signals of the Si–H groups, and in the case of ^1^H-NMR, also as the disappearance of distinct vinyl signals (see an example of H11–BAFKU2 in the Supplementary Materials in SI Figure S1a,b, respectively; all spectra are shown in SI Figures S1–S8.

The molecular masses of the prepared copolymers were evaluated by ^1^H-NMR spectroscopy (end-group analysis: ratio of terminal groups to PDMS repeat units), as well as by size exclusion chromatography (SEC, “GPC”) and by MALDI-TOF (matrix-assisted laser desorption ionization/time of flight mass spectroscopy; the results are shown in the Supplementary Materials). The latter two methods were useful for characterizing molecular mass distributions. The masses of the employed PDMS precursors and of the mesogen BAFKU also were evaluated. The results obtained by the different methods are all compared in SI Table S2 in the Supplementary Materials, where a detailed discussion is also included, together with all spectra. Generally, it was observed that the SEC analysis provided relatively accurate molecular mass values for all the PDMS–BAFKU_2_ copolymers, but less accurate ones for the PDMS precursors, especially for the low-molecular-weight H03, but also for H11. The SEC analysis also indicated the presence of some non-bonded BAFKU mesogen, ca. 5% of the total intensity of all peaks if the sensitive but relatively unbiased evaporative light scattering (ELSD) detector was used. This seems to be overestimated if compared to ^1^H-NMR data, where no characteristic peaks of the unreacted components were observed, neither vinyl (BAFKU excess) nor SiH (PDMS excess). Especially, in the case of the low-molecular-weight copolymer H03–BAFKU_2_, such residues in amounts as high as 5% would be well visible (estimated possible residue in the latter case would be below 1%). The overestimation of the BAFKU residue can be explained by the high/very high sensitivity of both the employed detectors to BAFKU, due to the employed wavelengths (see details in the discussion in the SI File). MALDI-TOF yielded very accurate values for the copolymers H03–BAFKU_2_ and H11–BAFKU_2_, but the vaporization of H21–BAFKU_2_ was no longer successful, similarly like in the case of neat H21, due to the too high molecular mass. The H03 precursor underwent radical coupling (oligomerization, SiH reactivity) during the laser pulses of the MALDI-TOF experiment, while H11 was characterized relatively accurately (SiH groups already diluted, *M*_n_ not too high).

To sum up, ^1^H-NMR spectroscopy was found to be the most sensitive method to verify the completeness of conversion during the synthesis (which was confirmed), and it also provided accurate values of the number average molecular masses of the PDMS precursors, as well as of the studied copolymers. The careful evaluation of SEC data also indicates only minimal amounts of unreacted components (BAFKU).

### 3.2. Thermo-Mechanical Properties

The reversible physical crosslinking in the prepared copolymers, as well as their rubbery properties, were investigated by dynamic-mechanical thermal analysis (DMTA) using parallel-plate tools with small plates (diameter 6.1 mm). This geometry made possible reasonably accurate characterization in the glassy and rubbery state, as well as in the early stages of melting. 

**Figure 1:**
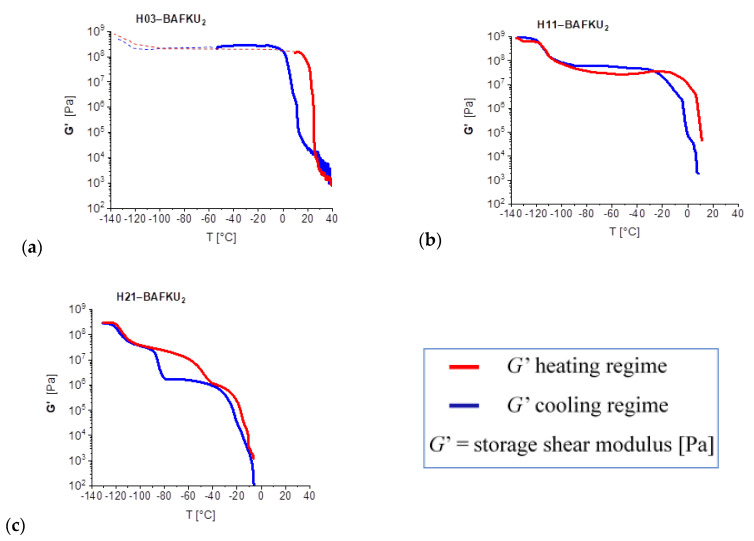
Dynamic-mechanical thermal analysis (DMTA) profiles (temperature-dependent storage modulus G’ only) of the copolymers (**a**) H03–BAFKU_2_, (**b**) H11–BAFKU_2_, and (**c**) H21–BAFKU_2_, recorded as heating and cooling scans.

Figure 1 illustrates the temperature dependence of the storage modulus in a heating and in a cooling run for all three studied copolymers. It can be observed that the copolymers with the longer PDMS chains, H11–BAFKU_2_ and H21–BAFKU_2_, behave like typical reversible elastomeric networks; they display a “rubbery plateau” with a storage modulus above (or well above) 1 MPa in relatively extended low-temperature regions. The short copolymer H03–BAFKU_2_, on the other hand, is a typical vitrimer; it undergoes a transition from the glassy (or possibly crystalline) state directly into a melt. The chain length of the PDMS segments has a marked effect on the rubbery behavior of the other two copolymers; the one with the mid-sized PDMS chains, namely H11–BAFKU_2_, displays the wider rubbery plateau (extent: 95 °C in heating scan, and 75 °C in cooling scan) and a higher melting point (ca. 8 °C; freezing point: 0 to −5 °C). Not surprisingly, due to the shorter elastic chains, the rubbery modulus of H11–BAFKU_2_ is also higher than in the case of the longer H21–BAFKU_2_; the width of the rubber plateau in H21–BAFKU_2_ is 20 °C while heating, and 55 °C while cooling, and the melting point is ca. −15 °C, while the freezing point is −20 °C.

The trend can be explained as follows. In the case of the very short H03 PDMS chain, the volume fraction of BAFKU mesogen makes up 52.4% (see Scheme 4) and it already dominates the thermomechanical properties of the material, which is either a crystalline molecular solid or a melt, depending on the temperature. In H11–BAFKU_2_ (37.3 vol% BAFKU), the elastic chains are sufficiently long for generating rubbery behavior, while the large volume fraction of BAFKU ensures highly efficient crosslinking via nano-aggregation of these terminal building blocks into domains which act as multiple junctions in the rubber (see further above, Scheme 1). Finally, in H21–BAFKU_2_ (13.5 vol% BAFKU), the relatively small mesogenic units are already considerably diluted in the mass of the PDMS chains, so that their aggregation is less efficient, resulting in a narrower rubbery plateau and a lower melting point.

H11–BAFKU_2_, the best low-temperature elastomer among the studied copolymers, displays a fairly high rubbery modulus in its extended rubber plateau, namely 30 MPa in the heating run and 60 MPa in the cooling run (see Figure 1b). It undergoes a glass transition near −110 °C. The melting region of the rubbery phase near 8 °C (freezing point: 0 °C; melting region—heating scan: 0 to 11 °C; cooling scan: −20 to 10 °C) is relatively steep, but still gradual, in contrast to molecular solids, which would melt abruptly. Some thermotropic transitions of the BAFKU aggregates can be suspected to occur in the melting region (progressive loosening or dissociation of the aggregates), in view of the step-like course of the modulus in this region. Additionally, gelation-type thermotropic transitions in the liquid region were confirmed by highly sensitive multi-frequency rheological tests (carried out using large parallel plates), which are discussed in a follow-on work [43]. The moderate increase in the modulus of H11–BAFKU_2_ with rising temperature practically in the whole rubbery region, which is typical of ideal elastomers, indicates an efficient physical crosslinking.

H21–BAFKU_2_, which is also a low-temperature elastomer (see Figure 1c), displays a much more complex behavior than H11–BAFKU_2_. In the heating scan, it displays only a very small rubbery region, or rather, a sloped step, but in the cooling scan, a much wider thermodynamically metastable rubbery plateau is observed. In both cases, the rubbery modulus is around 1 MPa. The vast difference in the width of the rubbery plateau during cooling and heating is caused by the behavior of the long linear PDMS chains in H21–BAFKU_2_. The latter display glass transition (−85 °C during cooling scan)/hidden cold crystallization (gradually occurring below −85 °C but above −110 °C)/melting (at −50 °C: during heating after cold crystallization). Such a behavior in pure PDMS is known from the literature, e.g., from [48]. Near −110 °C, there is a small step in the *G*’ = f(*T*) curve of H21–BAFKU_2_, which is assigned to freezing/unfreezing of segmental movements of the pendant methyl groups in PDMS chains, and which could be referred to as a minor glass transition. Below −110 °C, the H21–BAFKU_2_ copolymer is fully frozen and ideally glassy. It can be noted that in the case of the H11–BAFKU_2_ discussed above (see Figure 1b), which has shorter PDMS chains than H21–BAFKU_2_, only a single glass transition of the material occurs, namely at the temperature of the freezing of methyl groups movement in PDMS (−110 °C), while the vitrification/cold crystallization/melting hysteresis loop of PDMS is absent: the H11 chains are too short for such a behavior. The *G*’ = f(*T*) curve of H21–BAFKU_2_ is always decreasing with rising temperature in the rubbery plateau, which indicates that the physical crosslinking is not very efficient, and that some nanoaggregates of BAFKU end-groups dissociate even at temperatures relatively distant from the melting region. The onset of the melting (near −20 °C) is also distinctly more gradual (“melting point” itself is at ca. −15 °C) in H21–BAFKU_2_ than in H11–BAFKU_2_. (Freezing point of H21–BAFKU_2_: −20 °C melting region: −25 to 0 °C in heating and cooling scans). Although the rubbery plateau is small in H21–BAFKU_2_, the specific hysteresis behavior of this material could make it an interesting viscoelastic low-temperature rubber/oil.

H03–BAFKU_2_, the copolymer with the shortest elastic polysiloxane segments, displays very simple and rather unattractive thermomechanical characteristics (see Figure 1a). It behaves like a brittle “vitrimer”, which near 22 °C transforms directly from glass into melt (softening onset already at 14 °C), with no glass-to-rubber transition (freezing point of H03–BAFKU_2_: 5 °C melting region: heating scan: 15 to 25 °C; cooling scan: 0 to 25 °C). The melting transition is very steep in the heating run, but in the case of the cooling run, the freezing region is somewhat structured, thus indicating possible thermotropic transitions in the physical crosslinks in this temperature region. Rheological tests, which are discussed in a follow-on work [43], indeed confirmed this suspicion. The difference between the melting and the freezing temperature is also fairly high in H03–BAFKU_2_ (the difference is smaller in H11–BAFKU_2_ and nearly negligible in H21–BAFKU_2_). Due to its brittleness in the glassy state, it was impossible to study its thermomechanical properties below −40 °C, but in analogy to the other studied PDMS copolymers, a small step in the *G*’ = f(*T*) curve (dotted line in Figure 1a) would be expected in the range from −100 to −90 °C, caused by the freezing/unfreezing of the wagging movement of pendant methyl groups on PDMS segments. The H03–BAFKU_2_ copolymer might be of interest as a model compound or as a “plasticized BAFKU dimer”.

### 3.3. Phase Transitions in the Copolymers

#### 3.3.1. DSC: Specific Heat of Phase Transitions in the Copolymers

**Figure 2:**
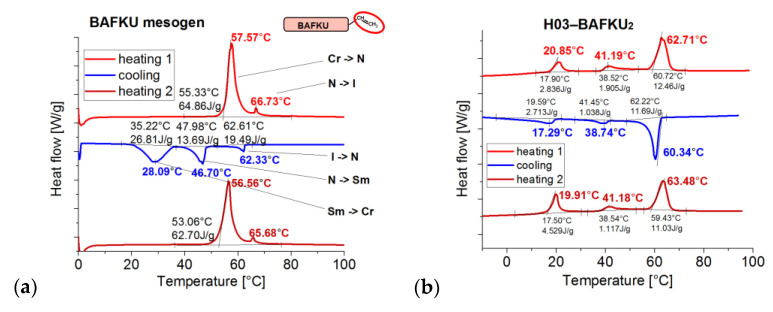
Differential scanning calorimetry (DSC) traces (**a**) of the neat BAFKU mesogen and (**b**) of the copolymer H03–BAFKU_2_.

**Figure 3:**
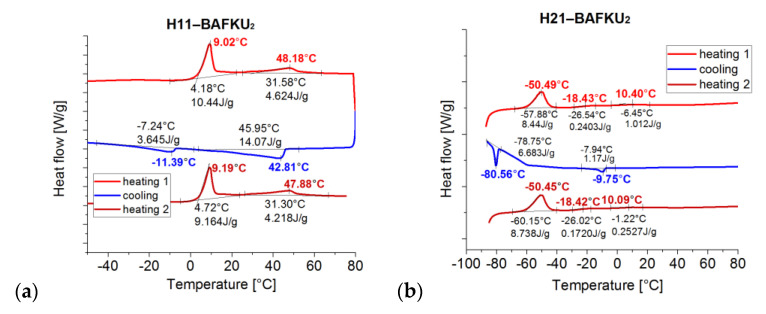
DSC traces of the copolymers (**a**) H11–BAFKU_2_ and (**b**) H21–BAFKU_2_.

The thermally induced transitions, which were observed by DMTA in the prepared copolymers, were further investigated by means of differential scanning calorimetry (DSC), in order to find out the nature and especially the involved values of specific heat of these transitions. The results are summarized in Figure 2 and Figure 3. The DSC trace of the neat mesogen BAFKU is also shown in Figure 2a for comparison.

The following trends can be observed. The specific heat values of the observed LC transitions distinctly decrease with increasing PDMS chain length, especially if going from H11 to H21.

Neat BAFKU in the heating DSC scan undergoes only the shorter phase sequence crystalline (Cr) →smectic C (SmC)→isotropic (I) [44], while in the cooling run, it undergoes the “full” phase sequence I→nematic (N)→smectic (Sm)→Cr transitions. The free mesogen is hence characterized by several monotropic transitions.

H03–BAFKU_2_ (see Figure 2b), the copolymer with the shortest PDMS chains, displays highly similar transition temperatures in its DSC trace like neat BAFKU in its cooling scan (compare Figure 2a,b), but all the transitions of H03–BAFKU_2_ are shifted to slightly lower temperatures. There are also some additional notable differences like different peak intensities, but also in the strictly enantiotropic nature of all transitions in H03–BAFKU_2_, with nearly identical specific heat values of the reverse processes. Notable in this context is also the near absence of undercooling effects in the copolymer. The N→I transition is always the most intense one in the copolymer, while in neat BAFKU, I→N is low-intensity and monotropic. The transitions in H03–BAFKU_2_ were assigned by correlating the DSC results with polarized light microscopy analysis discussed further below: highest temperature—N/I; medium temperature—Sm/N; lowest temperature—Cr/Sm. The slight down-shift of the transition temperatures in the copolymer (if compared to neat BAFKU) can be attributed to the moderately increased mobility of the mesogen units, which are attached to short, monodisperse (PDI = 1.11, see SI Table S2 in the Supplementary Materials), and highly flexible PDMS chains. These specific short spacers also apparently similarly strongly stabilize the smectic as well as the nematic phase in the copolymer, which is still dominated by BAFKU (52 vol%, see Table 1), thus leading to the absence of undercooling.

It can be further noted that H03–BAFKU_2_ melts at ca. 22 °C (see DMTA in Figure 1a), near its first thermotropic transition (19.9 °C, Cr→Sm), while the remaining transitions occur in the liquid phase (up to 63 °C). This means that residual ordered liquid crystalline aggregates must persist as branching points in the copolymer melt from 22 to 63 °C (N→I) and thus, can have interesting effects on its viscoelastic behavior.

H11–BAFKU_2_ (see Figure 3a) displays a smaller number of transitions than H03–BAFKU_2_, two instead of three, and also, the characteristic temperatures are lower in H11–BAFKU_2_. The “width” of the temperature range of the combined LC transitions in H11–BAFKU_2_ is practically the same as in H03–BAFKU_2_, however, suggesting that similar interactions play a role in both copolymers. The down-shift of the transitions in H11–BAFKU_2_ indicates a higher mobility of BAFKU due to the attached relatively long and segmentally very mobile of PDMS chains. In addition, the “dilution” of the aggregates in the now dominant PDMS phase (63 vol%, see Table 1) may play a role. The DSC peaks of H11–BAFKU_2_ were assigned in view of the polarization microscopy textures discussed further below as Cr/N (lower temperature) and N/I (flat peak extended over a wider region). In the case of the Cr/N peak, if the heating and cooling scans are compared, the change of the intensity (much less intense if cooling) and of the characteristic temperature (down-shift by 20 °C if cooling), indicates a difficult crystallization. This effect was also observed by PLM (as discussed further below), and it correlates with the down-shifted solidification of molten H11–BAFKU_2_ to rubber, which was observed by DMTA (see Figure 1b). On the other hand, the N/I transition in the melt displays only a small undercooling effect. This latter transition also shows the highest specific heat among the peaks in the cooling run, thus indicating that the formation of the nematic phase is fairly strongly favored by the H11 chains. The absence of a smectic phase in H11–BAFKU_2_ indicates that the medium–long H11 chains, albeit monodisperse, do not stabilize the highly ordered Sm phase in contrast to short H03 chains.

H21–BAFKU_2_ (see Figure 3b) displays practically no visible LC transitions in heating scans, and one weak but distinct transition in the cooling scan at −9.8 °C (quasi a monotropic one), which is positioned in the region of the onset of the solidification of the melt to rubber (see further above, DMTA) and was assigned to the N→Cr transition in the BAFKU nanoaggregates of H21–BAFKU_2_. The polarization microscopy (PLM) investigations, and also the X-ray-diffraction (XRD) patterns, which are both discussed further below, support the existence of BAFKU nanodomains (lamellae) in H21–BAFKU_2_, also in an extended temperature region of the liquid state. If an analogy would be drawn to the shorter H11–BAFKU_2_, the longer copolymer H21–BAFKU_2_ also could contain its mesogen nanoaggregates in the nematic ordering state. The anisotropic domains in PLM (see further below) are very small, however, and the textures are not very characteristic, so the assignment of the nematic phase in H21–BAFKU_2_ is only tentative. The suggested analogy between H21–BAFKU_2_ and H11–BAFKU_2_ is further supported by the analogous rheological behavior of both copolymers, which was studied in a separate work [43]. The low transition temperature, as well as the quasi-monotropic character (and small specific heat) of the single thermotropic transition observed in H21–BAFKU_2_ suggest the formation only of weaker and smaller BAFKU aggregates in this longest copolymer, in contrast to the shorter ones. It can be clearly concluded that in H21–BAFKU_2_, the effect of BAFKU on the thermal properties is diminutive. In spite of this, the physical crosslinking as observed by DMTA still is considerably strong in this copolymer, albeit it is the weakest in the compared series.

In contrast to the other copolymers, the DSC traces of H21–BAFKU_2_ additionally display distinct thermal transitions of the long PDMS chains of H21 (vitrification/cold crystallization/melting), which in fact dominate the whole DSC trace. The region of these transitions is marked by a frame in Figure 3b. In the heating scans, a broad melting peak is observed with a maximum at −50.5 °C, while in the cooling scan, a sloped course of the DSC trace, which corresponds to a glass transition, is observed between −60 and −85 °C, which also contains a cold crystallization peak (−80.6 °C). The vitrification/cold crystallization/melting of PDMS was discussed in detail in the literature, e.g., in [48].

#### 3.3.2. X-ray Diffraction (XRD) Analysis of the Phase Transitions

**Figure 4:**
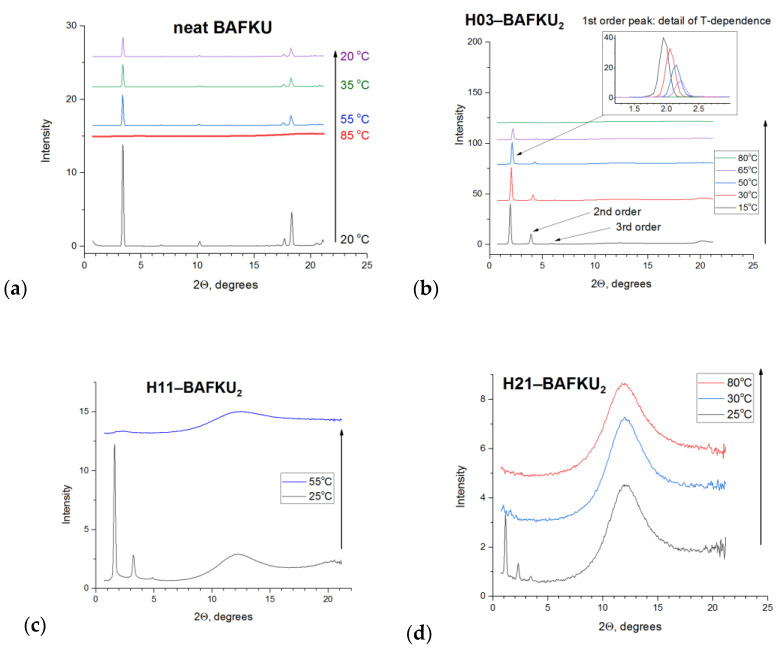
Temperature-dependent XRD patterns of: (**a**) neat BAFKU, (**b**) H03–BAFKU_2_, (**c**) H11–BAFKU_2_, and (**d**) H21–BAFKU_2_; in the case of neat BAFKU (**a**), the sample was first characterized at 20 °C, then heated up to 85 °C, and subsequently, step-wise cooled down back to 20 °C. This was done because the full series of phase transitions in BAFKU occurs only in the cooling scan; in the case of all the copolymer samples (**b**–**d**), they were step-wise heated from low temperature to high temperature during the XRD analyses.

The phase transition behavior of the studied copolymers was deeper elucidated by means of recording temperature-dependent X-ray scattering (small-angle X-ray scattering SAXS and wide-angle X-ray scattering WAXS) of the products and by comparing them with the patterns of the pure mesogen BAFKU. The results of the X-ray experiments are summarized in Figure 4. The observed XRD peaks are commented on in Table 2 and Table 3. Generally, all three copolymers were found to possess a distinctly lamellar structure, with BAFKU nanoaggregates as the lamellae.

In the case of the neat BAFKU (Figure 4a), the following assignment to the dimensions of the mesogen molecule can be done. The peak at 3.40° (scattering vector *q* = 0.242 Å^−1^, corresponding to the distance *d* = 2.60 nm) can be well correlated with the length of this rod-like building block, while the very weak peak at 6.80° (0.484 Å^−1^) appears to be a second-order reflection of the same distance. The peak at 10.18° (0.72 Å^−1^/0.87 nm—it is more intense than the one at 6.80°, and hence, it cannot be the 3rd order of the peak at 3.40°) can be assigned to the width of the whole mesogen (similarly like in [44]). Finally, both the peaks in the range of 18–20° (1.4 Å^−1^/0.45 nm) can be assigned to standard Van der Waals distances between organic molecules, e.g., in different directions in the crystal. The peaks of the neat mesogen are all sharp, thus indicating a highly ordered state at all the tested temperatures, except in the isotropic melt at 85 °C (see Figure 4a).

**Scheme 5:**
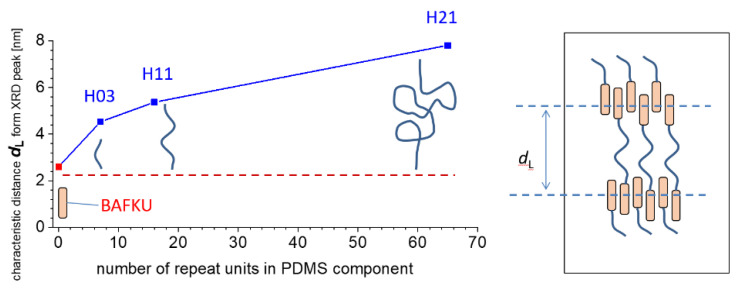
Lamellar structure of the copolymers and the effect of the chain length of the PDMS component on the characteristic distance observed by X-ray diffraction. (**Left**): plot of the observed (XRD) characteristic distance with in-laid approximate relative sizes of the segments of the studied copolymers; the coiling of PDMS chains plays an important role; (**Right**): idealized representation of the lamellar nanoaggregates of the BAFKU end-groups present in the copolymers below the isotropization temperature.

**Scheme 6:**
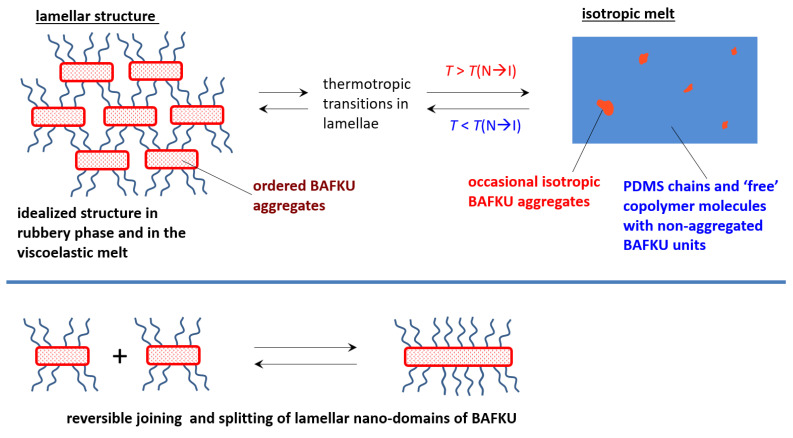
Postulated lamellar structure in the studied copolymers, composed of lamellar nanoaggregates of BAFKU and of elastic PDMS chains, its isotropization (according to Scheme 7 **bottom**), as well as the reversible growth and fragmentation of the lamellar BAFKU nanoaggregates.

In the case of the PDMS–BAFKU_2_ copolymers (Figure 4b–d), the BAFKU units also generate sharp X-ray diffractions, but their diffraction pattern is much simpler. Only one characteristic distance is observed, which generates a main peak, as well as second- and third-order reflections of this distance. This characteristic distance in the BAFKU domains of the copolymers becomes distinctly wider with the increasing length of the polysiloxane spring. The presence of such distinct 2nd and 3rd order reflections, as well as the relatively sharp peaks, are indicative of a well-developed lamellar structure (see Scheme 5 and Scheme 6 and structure discussion further below). The increasing characteristic distance corresponding to these reflections can be well correlated with the increasing spatial demand of the increasingly long and increasingly coiled PDMS chains (see Scheme 5), which separate the lamellae. Additionally, two very broad peaks (near 12° and near 21°) appear in the diffractograms of the copolymers, corresponding to amorphous halo structure, which is characteristic of PDMS. Especially, the first PDMS peak dramatically grows with increasing chain length of the PDMS component, and hence, with the PDMS fraction in the copolymer, while the second is only partly visible.

The diffractograms of the sample H03–BAFKU_2_ (Figure 4b) were measured at temperatures between and beyond the ones of the characteristic DSC transitions (discussed further above), namely at 15, 30, 50, 65, and 80 °C. We can see a systematic shift of the main reflection peak of the BAFKU domains with increasing temperature. Originally, the peak is positioned at 2θ = 1.96°, which corresponds to d = 4.50 nm (2nd order reflection at 3.94°; 3rd order at 5.87°). Sample heating leads to a higher angle 2θ, which means a shorter characteristic distance. A shorter distance between the lamellar BAFKU aggregates at higher temperatures could be explained by a more coiled average conformation of the PDMS chains, which is a known entropy effect in elastomers. The change of 2θ with *T* is almost linear, starting with 4.498 nm at 15 °C, via 4.288 nm at 30 °C, 4.114 nm at 50 °C, and, finally, 3.959 nm at 65 °C. Like the main reflection, the higher order interference maxima all show a proportional *T*-induced shift. Analogous behavior is observed for the reflections in all the copolymers (compare Figure 4b–d). The intensity of the peaks originating from BAFKU units gradually decreases with the temperature, indicating partial melting (dissociation of larger aggregates). No peaks are observed at 80 °C, which is in agreement with the isotropization of H03–BAFKU_2_ observed by DSC already at 63 °C (see further above).

The sample H11–BAFKU_2_ (Figure 4c) exhibits an analogous behavior like H03–BAFKU_2_, but the fundamental peak of its single reflection is found at 2θ = 1.62° (2nd order at 3.25°; 3rd at 4.88°), which corresponds to 5.435 nm. The distance is 21% larger than the characteristic one in H03–BAFKU_2_. Similarly, like in the latter sample, no XRD reflections of BAFKU are observed in the isotropic melt state of H11–BAFKU_2_.

The similarly behaving H21–BAFKU_2_ (Figure 4d) exhibits the largest characteristic distance in the BAFKU domains. At 25 °C, already in the liquid range (nematic), the sharp reflection is positioned at 2θ = 1.15° (2nd order at 2.31°; 3rd at 3.43°), which corresponds to d = 7.69 nm. This reflection is no longer observed at 30 °C, where the melt already is fully isotropic. No changes in amorphous halo were observed at higher temperature (between 25 and 80 °C). While the sensitivity of DSC was too low to detect phase transitions in H21–BAFKU_2_ in the heating regime, the X-ray results together with the light microscopy data presented further below indicate a phase transition (isotropization) near 30 °C, which to some extent, is also confirmed by rheology tests, which are discussed in a follow-on work [43].

#### 3.3.3. PLM Observation of Anisotropy and of Phase Transitions

**Figure 5:**
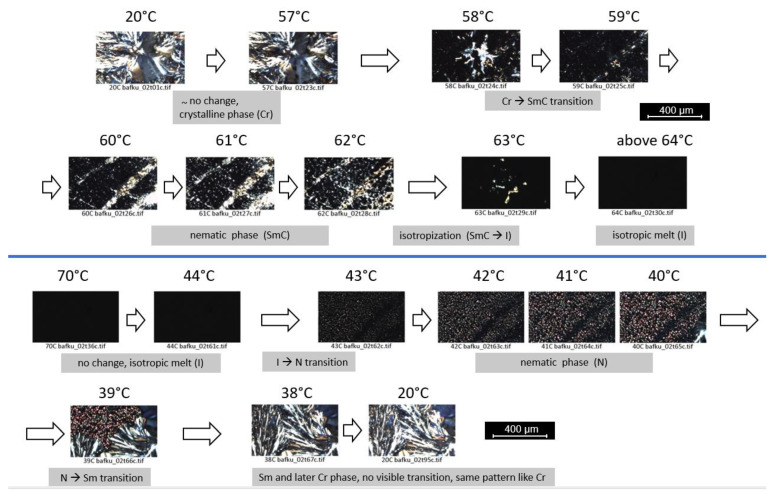
Textures observed in neat BAFKU mesogen. **Top**: heating run; **bottom**: cooling run; large representative images of each characteristic texture are shown in the Supplementary Materials, SI Figures S20 and S21.

**Figure 6:**
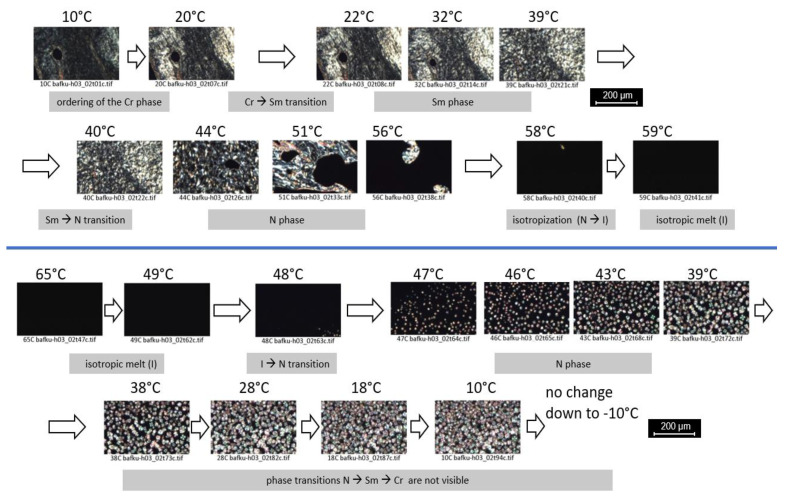
Textures observed in BAFKU–H03. **Top**: heating run; **bottom**: cooling run; large representative images of each characteristic texture are shown in the Supplementary Materials, SI Figures S22–S24.

**Figure 7:**
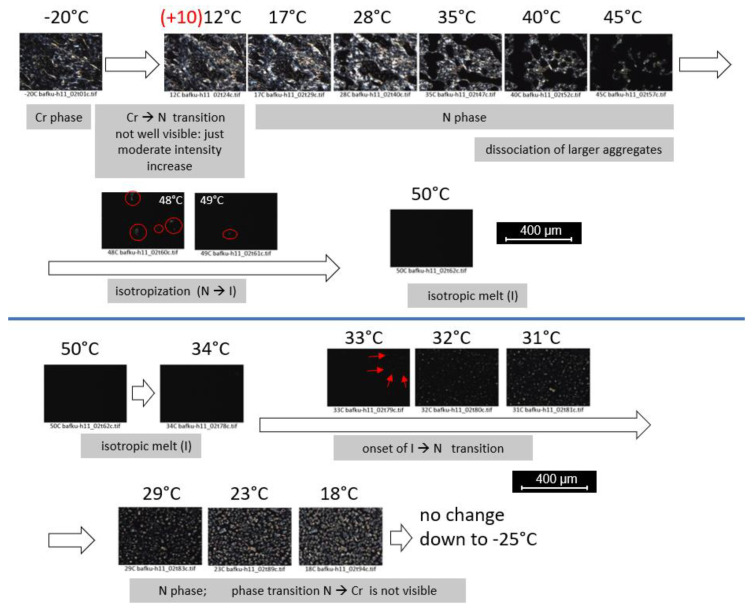
Textures observed in BAFKU–H11. **Top**: heating run; **bottom**: cooling run; large representative images of each characteristic texture are shown in the Supplementary Materials, SI Figures S25–S27.

**Figure 8:**
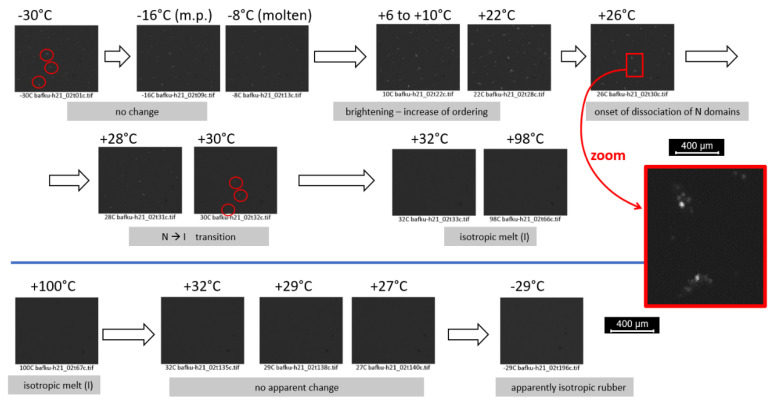
Textures observed in BAFKU–H21, top: heating run; **bottom**: cooling run; large representative images of each characteristic texture are shown in the Supplementary Materials, SI Figures S28–S30.

The phase transition behavior as well as ordering effects in the domains of the mesogenic units of the prepared copolymers were additionally evaluated by means of polarized light microscopy (PLM). The PLM analysis was of special interest, because the XRD patterns discussed above were dominated by the reflections caused by lamellae of BAFKU and on PDMS interchain distances, but these patterns did not provide any information about the arrangement in the lamellae. The PLM results are summarized in Figure 5, Figure 6, Figure 7 and Figure 8. It should be noted that the extended sub-millimeter-sized highly anisotropic regions observed in the copolymer samples at appropriate temperatures cannot be lamellae of aggregated and well-separated BAFKU units, in view of their volume fractions in the copolymers (see Table 1 and Scheme 4), and also in view of their bonding situation (see Scheme 1), which does not allow for the growth of very large 3D domains made from phase-separated pure BAFKU. The observed anisotropic areas hence can be assigned to regions in which the lamellar BAFKU nanodomains have some prevalent orientation, while being separated by PDMS, so that both the ordering inside of the nanodomains (lamellae), as well as their “global arrangement” (some analogy to lyotropic systems), contribute to the dichroism of the respective observed specimen.

As the reference compound, the neat mesogen BAFKU was also characterized. It can be seen in Figure 5 (representative high-resolution images of the characteristic textures are shown in SI Figures S20 and S21 in the Supplementary Materials) that its changes of PLM texture during the heating run fairly closely follow the temperature course of the phase transitions observed by DSC (see Figure 2a); one abrupt change is observed at 58 °C (DSC peak maximum: 57 °C: Cr→SmC, as assigned in [44]), while the second occurs between 63 and 64 °C (DSC: 67 °C: SmC→I). A fan-shape texture is observed for the crystalline phase. The transition at 58 °C is followed by a temporary darkening (isotropization), which precedes the formation of new, differently ordered extended textures. The second transition (SmC→I) occurs at a somewhat lower temperature in PLM than in DSC, which might be the result of a higher rate in the DSC scan (10 vs. 1 °C/min) and/or of the dissolution of the larger oriented domains of BAFKU molecules. This would lead to “earlier” apparent disappearance of anisotropy in PLM. In the cooling run studied by PLM (Figure 5), a distinct apparent undercooling is observed in comparison to the DSC scan in Figure 2a for the I→N transition, which starts at 43 °C in PLM (DSC: 62 °C), but this is possibly a consequence of the fact that only larger oriented domains or regions become visible (similarly like in the case of heating). This undercooling is also observed if the mean polarized light intensity from the whole image area is evaluated (see SI Figure S16 in the Supplementary Materials). The formation of small anisotropic domains directly invisible by the microscope would manifest itself as an increase in background light intensity, but due to an efficient undercooling, no such “hidden” intensity increase is observed (in contrast to some other samples). The next PLM-observed transition, N→Sm (fan-shape structure appears, similar to the original crystal), starting at 40 °C (well visible at 39 °C, finished at 38 °C) is less low-temperature shifted (47 °C in DSC). Both low-temperature shifts in the cooling PLM run might be a result of the narrow (<10 µm) distance of the microscopy glass platelets, which might cause a more difficult crystallization than in the thicker DSC samples. Because the texture observed after the N→Sm transition of BAFKU is already practically identical with the one observed for the crystalline phase (see heating scan in Figure 5), the Sm→Cr transition of neat BAFKU (DSC: 28 °C) cannot be recognized by PLM.

The H03–BAFKU_2_ copolymer in a certain sense displays similar trends in the temperature-dependent PLM textures (Figure 6; representative high-resolution images of the characteristic textures are shown in SI Figures S22–S24 in the Supplementary Materials) like neat BAFKU, but the textures are of somewhat different appearance and their interconversions are rather gradual than abrupt, in contrast to PLM of BAFKU, or to the DSC traces of this same copolymer, H03–BAFKU_2_, where the peaks are fairly sharp and where only negligible undercooling is observed. Between 10 and 20 °C in the heating run, an apparent increase in long-range ordering of H03–BAFKU_2_ can be observed, which manifests itself by increasing texture intensity, without any change of pattern. Above 20 °C (Cr→Sm transition in DSC), the intensity does not change any more, but while the global pattern does not change markedly (nor do the colors), the fine pattern of the texture changes from fibrillar to particulate. Above 40 °C, the domains of the fine pattern at first visibly increase in size and change their color (DSC: 41 °C: Sm→N). As the temperature further rises, the fraction of anisotropic areas decreases, while larger apparently isotropic domains start to grow (considerable isotropization at 56 °C and nearly complete one at 58 °C). In DSC (Figure 2b), the isotropization (N→I) peak is observed near 63 °C. The “earlier” occurrence of the isotropization in the PLM scan can be attributed to the dissolution of the larger regions with oriented nano-lamellae of BAFKU units in the nematic state, in some analogy to neat BAFKU.

In the cooling PLM experiment, the H03–BAFKU_2_ copolymer displays even greater undercooling effects than neat BAFKU; the onset of visible nematic ordering (nematic dots) is observed near 48/47 °C (DSC peak: 60 °C). At lower temperatures, these “dots” grow and finally cover the whole observed area, but no change of pattern is observed at any temperature (in DSC, peaks are observed at 39 and 17 °C), as if the sample froze to a nematic glass. This behavior may be attributed to the thin sample layer (similarly like in neat BAFKU). Efficient undercooling is also confirmed, if the mean polarized light intensity from the whole image area is evaluated (see SI—Figure S17 in the Supplementary Materials). No “hidden” intensity increase is observed prior to the appearance of the visible patterns at 48/47 °C. The sample undergoes slow “ageing” at room temperature, however. After 3 days, the dot texture, which was obtained at the end of the cooling run, changes to the texture observed for the smectic phase in the heating run (very similar to the crystalline one). Theoretically, this slow ageing could be explained in two ways—either the transition N→Sm is very slow and gradual or the observed texture is strongly influenced by the arrangement of slowly growing lamellae to large “global” patterns. The second explanation is supported (and the first disproven) by the fact, that the DSC trace of H03–BAFKU_2_ displays nearly identical transition temperatures and peak intensities (and shapes) in both heating and cooling runs. The role of the lamellae (texture generation similar to lyotropic systems) also would explain why the textures of H03–BAFKU_2_ change so gradually also in the heating scan, where no “delaying effects” would be expected (as the DSC transitions are very sharp).

The H11–BAFKU_2_ copolymer, which is richer in PDMS, displays similar but simpler trends (see PLM textures in Figure 7; representative high-resolution images of the characteristic textures are shown in SI Figures S25–S27 in the Supplementary Materials) like H03–BAFKU_2_. In the heating run, the difference is small between the texture of the sample with crystalline domains of BAFKU (according to DSC in Figure 3a, below 10 °C) and between the subsequently occurring liquid crystalline state at higher temperature, assigned as nematic. The transition at 10 °C mainly manifests itself as a subsequent very gradual intensity decrease in the rotated light. Above 35 °C, a progressing apparent isotropization (decay of larger regions with oriented lamellae) is observed, and the visible anisotropic domains nearly disappear at 45 °C (last smallest remnants until 49 °C). In DSC, the maximum of the corresponding N→I peak is observed at 48 °C. As the copolymer H11–BAFKU_2_ displays only one liquid crystalline phase in melt, no distinct changes in texture type are observed above 10 °C.

In the cooling run, the H11–BAFKU_2_ copolymer behaves very similarly like H03–BAFKU_2_. It seems to undercool and to apparently freeze to a nematic glass. The process weakly sets on at 33 °C (DSC: I→N transition: 43 °C) and becomes well-visible at 29 °C. The nematic dot domains grow less dramatically than in H03–BAFKU_2_. No change in texture is observed upon further cooling. If the mean polarized light intensity from the whole image area is evaluated (see SI Figure S18a), then an increase in intensity is observed, which already starts during the cooling run just below 38 °C, only 5 °C below the DSC transition where small BAFKU nano-lamellae are expected to start to assemble. Hence, the apparent undercooling does not mean a complete absence of BAFKU aggregation in this whole temperature region. The easier ordering of the mesogenic units of H11–BAFKU_2_ in the cooling scan, if compared to H03–BAFKU_2_, could be attributed to a higher mobility of the BAFKU units attached to the longer flexible chains. In the case of the heating run of H11–BAFKU_2_ (see SI Figure S18a), the light intensity curve closely follows the trends noted by “simple” PLM observation, as would be expected.

The H21–BAFKU_2_ copolymer, which is the richest in PDMS, displays much simpler PLM textures (see Figure 8; representative high-resolution images of the characteristic textures are shown in SI Figures S28–S30 in the Supplementary Materials) than H03–BAFKU_2_ and H11–BAFKU_2_.

In the heating run, only small dots are observed in H21–BAFKU_2_ for the rubbery state (with solid BAFKU aggregates), as well as in melt until 30 °C. The texture is somewhat similar like the nematic dots observed in H11–BAFKU_2_ during its cooling near 31 °C, but the dots are smaller and less characteristic in the case of H21–BAFKU_2_. The melting of rubbery H21–BAFKU_2_ near −15 °C does not cause any marked change in the texture. The critical temperature is near 30 °C, where XRD experiments observe the disappearance of the ordering of the LC units in H21–BAFKU_2_, and where rheology experiments, which are discussed in a follow-on work [43], suggest a drop in elasticity. While approaching this temperature in the heating scan (PLM), the small dots intensely brighten. The brightening occurs between 6 and 10 °C, the high intensity persists until 22 °C, thereafter it fades until 28 °C, and completely disappears above 30 °C (efficient dynamic disconnection of nanoaggregates of LC units). The ordering which causes the mentioned brightening of the texture can be attributed to an increased mobility of the BAFKU units.

Similarly, like in the case of the other copolymers, the cooling scan of H21–BAFKU_2_ yields different results (apparent “undercooling”), then would be expected in view of DSC results (H21–BAFKU_2_ has a distinct peak at −9.8 °C), or of the known solidification point (melt→rubber, observed by DMTA, −15 to −20 °C for H21–BAFKU_2_). No change in texture appears during the whole cooling scan of H21–BAFKU_2_, the texture remains isotropic, down to −29 °C. Similarly, like in the case of the other copolymers, this “undercooling” might be the result of the slow formation of extended ordered regions of the lamellar structure observed in XRD, if the sample is present as a very thin layer (PLM). Small randomly oriented lamellar regions do not generate sufficient optical anisotropy to be simply visible by PLM. If, however, the mean polarized light intensity from the whole image area is evaluated (see SI Figure S19b), then, like in the case of H11–BAFKU_2_, an increase in intensity is observed in the “undercooled isotropic region”. It starts during the cooling run just below 30 °C, where in view of the X-ray (further above) and rheology results, which are discussed in a follow-on work [43], small BAFKU nano-lamellae are expected to start to assemble. In the case of the heating run, (see SI Figure S19a), the light intensity curve follows the trends noted by the “simple” PLM observation.

#### 3.3.4. Structure of the BAFKU Aggregates

In view of the combined characteristics of the copolymers obtained by DSC, XRD, and by polarization microscopy (PLM), as well as by thermomechanical analysis (DMTA), important conclusions can be drawn about the aggregating behavior in BAFKU end-groups. First, the single characteristic reflection (XRD) of BAFKU structures, accompanied by well-visible 2nd and 3rd order peaks, indicates a distinct lamellar structure in all the copolymers. Secondly, the different characteristic distances corresponding to the mentioned reflection in the different copolymers well correlate (see Scheme 5 further above) with the approximate “effective length” of the coiled PDMS chains in each of them, and thus, with the distance between the BAFKU lamellae (rather than with their thickness).

Depending on the temperature, the BAFKU aggregates change their internal arrangement, as indicated by DSC and PLM, but below the isotropization temperature, they always must be lamellar in shape, and the lamellae must be at regular distances (like in the example in Scheme 6 further above), in order to generate the mentioned relatively sharp XRD reflection combined with the higher order peaks. Larger areas with regularly and hence, anisotropically arranged BAFKU aggregates (lamellae) can generate textures visible by PLM, in contrast to small regions of regularity. The latter nevertheless can produce significant background intensity in PLM, which is not easily seen by the naked eye, but which still was possible to evaluate, as mentioned further above (see PLM analysis of H21–BAFKU_2_). 

Thermally induced fragmentation and recombination of BAFKU aggregates is also shown in Scheme 6, as well as the isotropization (maximum fragmentation and loss of ordering in the residual small dynamic aggregates or associates of BAFKU end-groups). Analogous aggregate reorganizations are also expected to occur in the case of strain damage and self-healing.

**Scheme 7:**
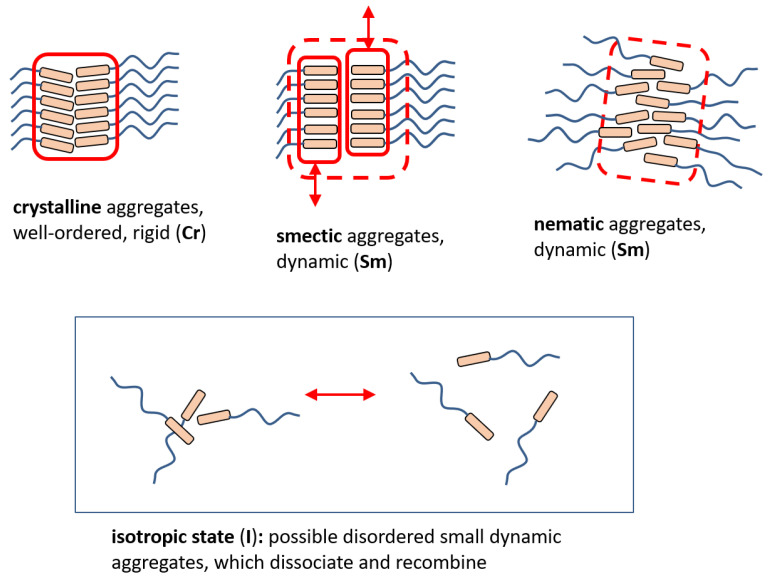
Postulated idealized structure in the crystalline (Cr), smectic (Sm), and nematic nanoaggregates of BAFKU end-groups in the studied copolymers, as well as the arrangement in the copolymers’ molecules in the isotropic melt.

The postulated thermotropic phase transitions in the BAFKU lamellae are illustrated in Scheme 7. In the rubbery state, the nanoaggregates (lamellae) are in the crystalline state and are rigid. In the BAFKU-rich H03–BAFKU_2_ copolymer, a smectic structure (as observed by PLM and DSC) is stable in the melt region in a favorable temperature range; the layers in the aggregates can move (see Scheme 7). Additionally, besides the mentioned shear deformation, the smectic aggregates are “dynamic”, and can undergo fragmentation and recombination (see Scheme 6). Such effects make possible the flow of the molten smectic copolymer, but the necessary reorganization of the aggregates during the flow consumes a considerable amount of energy, as is illustrated by rheology experiments discussed in a follow-up paper [43], including thixotropy effects (see e.g., the high storage and loss moduli of the melt of H11–BAFKU_2_ at 25 °C, after abrupt melt cooling, in the Supplementary Materials of [43]). The smectic state is not observed in the copolymers with the longer PDMS chains, H11–BAFKU_2_ and H21–BAFKU_2_, where, as demonstrated by DSC, the “nematic aggregate phase” is directly formed from the rigid aggregates during the melting of the rubbery copolymer.

The nematic-ordered aggregates are of “dynamic” nature, similarly like the smectic-ordered ones. In the molten H03–BAFKU_2_ copolymer, the thermotropic transition Sm→N occurs in the interior of the aggregates at a characteristic temperature, as observed by DSC (41.2 °C). In the “longer copolymers”, no difference in texture is observed between the “crystalline” and the “molten nematic” state of the mesogen aggregates, in both the heating and the cooling run, in strong contrast to neat BAFKU or H03–BAFKU_2_ (Cr→Sm: no change, but Sm→N: vast change). This implies that the “crystalline state” in the longer copolymers could in fact be a less ordered “frozen nematic” state. The somewhat different PLM textures observed during the heating and cooling runs of these longer copolymers could be the result of prolonged “aging” at room temperature in the nematic molten state, which always occurred (sample storage) prior to the initial heating run. The reversible reorganizations of the aggregates discussed above would namely allow for the growth of larger domains with anisotropic ordering during long storage, in contrast to the cooling PLM run (starting from isotropic melt, running at 1 °C/min), during which the samples dwelt only for a relatively short time in the nematic molten state. 

As mentioned in the section dedicated to the synthesis of the copolymers, the azo units incorporated in the BAFKU building blocks of the copolymers offer the potential of reversibly photo-switching the mesogenic units between the *cis*- and *trans*- state. In this way, the stability, the transition temperatures, as well as the tendency of the lamellae discussed above to grow or to split would be greatly altered, resulting in switching of elastic/viscoelastic properties.

## 4. Conclusions

Physically crosslinked thermo-reversible low-temperature rubbers with interesting viscoelastic properties in the melt state (very large step-wise changes of elasticity and viscosity) were successfully prepared; they are based on linear polydimethyl-siloxane (PDMS) capped in α,ω-positions with liquid crystalline (LC) building blocks called “BAFKU”. The LC building blocks make reversible UV light-induced switching of material properties possible.

Copolymers based on three different commercial α,ω-Si–H-functional PDMS molecules, namely DMS H03 (8.6-mer), DMS H11 (16.3-mer), and DMS H21 (64.4-mer), were obtained via hydrosilylation reactions of these PDMS precursors with the mono-vinyl-functional mesogen BAFKU.

Thermomechanical analysis (DMTA) proved that the PDMS–BAFKU_2_ copolymers, based on namely the H11–BAFKU_2_ and H21–BAFKU_2_, behave like typical reversible elastomer networks. The physical crosslinking via nano-aggregation of BAFKU units was found to be reversible by heat, as well as by organic solvents.

On the other hand, the copolymer based on the short polydimethylsiloxane DMS H03 is a brittle (vitrimeric) material, with no transition glass→rubber. Upon heating (DMTA), it transforms directly from glass to melt. This is due to the high-volume fraction (52 vol%) of rigid BAFKU.

DSC study of the phase behavior indicates similar sharp transitions in neat BAFKU and in LC-rich H03–BAFKU_2_ (three transitions at similar *T* values); H11–BAFKU_2_ shows different, less intense, simpler, but still multiple (two) and sharp, low-*T*-shifted transitions; H21–BAFKU_2_ displays no well-visible LC transitions upon heating, but one LC transition during the cooling scan; other methods (XRD, PLM) indicate, however, that H21–BAFKU_2_ displays two LC transitions, similarly like H11–BAFKU_2_; the increasing chain length of PDMS leads to higher mobility of BAFKU, thus down-shifting its LC transitions in the copolymers and reducing the number of stable LC phases to one, namely the nematicum.

XRD analyses indicate a distinct lamellar structure in all three PDMS–BAFKU copolymers; the lamellar structure (as branched macromolecule associates) was found to persist also in the molten state, until the isotropization transition temperature observed by DSC.

PLM investigations support the existence of smectic and nematic phases in the molten state of H03–BAFKU_2_, and of nematic phases in the melt of H11–BAFKU_2_ and H21–BAFKU_2_, as well as the transition temperatures observed by DSC and XRD; in the case of the copolymers with longer PDMS chains (H11, H21), the solid nanoaggregates of BAFKU end-groups seem to be of the frozen nematic type, rather than of a distinct crystalline one.

The studied low-temperature elastomers might be of interest as passive smart materials for advanced applications such as viscoelastic coupling (melting/gelation to rubber), but also as damping materials (energy absorption via physical crosslink disconnection).

A follow-up paper is dedicated to the effects of the structure of the studied copolymers on their viscoelastic and rheological properties in the rubbery state, as well as in melt.

## Figures and Tables

**Table 1:** Amounts of the components used in the synthesis of the main chain liquid crystalline copolymers based on polydimethylsiloxane (PDMS) and liquid crystalline (LC) mesogen.

	Components	BAFKU Mesogen				Precursor DMS H03				Precursor DMS H11				Precursor DMS H21				Chloroform		Karstedt’s Catalyst 2 wt.% Solution	
Sample Name																					
		[g]	[mmol] of C=C Groups	wt%	Vol%	[g]	[mmol] of SiH Groups	wt%	Vol%	[g]	[mmol] of SiH Groups	wt%	Vol%	[g]	[mmol] of SiH Groups	wt%	Vol%	[g]	[mL]	[g] as 2% Solution	[mmol]
**H03–BAFKU_2_**		0.5	1.19	57.4	52.44	0.371	1.19	42.6	47.56									2.956	24.76	0.0087	0.0228
**H11–BAFKU_2_**		0.5	1.19	41.43	37.29					0.711	1.19	58.57	62.71					2.956	24.76	0.0087	0.0228
**H21–BAFKU_2_**		0.5	1.19	15.1	13.47									2.832	1.19	84.9	86.53	2.956	24.76	0.0087	0.0228

**Table 2:** X-ray diffraction results. BAFKU: interference maxima and the corresponding characteristic distances.

BAFKU			
2θ [°]	q [Å^−1^]	d [nm]	comment
3.40	0.242	2.60	BAFKU length
6.80	0.484	1.30	-“- (second order of 3.40°)
10.18	0.724	0.87	BAFKU width
17.70	1.255	0.50	standard Van der Waals distance in crystal
18.36	1.301	0.48	standard Van der Waals distance in crystal

**Table 3:** X-ray diffraction results. PDMS–BAFKU_2_ copolymers: Interference maxima and the corresponding characteristic distances.

H03			H11			H21			
2θ [°]	q [Å^−1^]	d [nm]	2θ [°]	q [Å^−1^]	d [nm]	2θ [°]	q [Å^−1^]	d [nm]	comment
1.95	0.139	4.53	1.644	0.117	5.37	1.13	0.0804	7.81	main reflection
3.90	0.277	2.26	3.28	0.233	2.69	2.26	0.161	3.90	2nd order
5.85	0.416	1.509	4.93	0.351	1.79	3.39	0.241	2.60	3rd order
8–16	0.57–1.13	1.1–0.55	8–16 (12.24)	0.57–1.13 (0.896)	1.1–0.55 (0.723)	8–16 (12.24)	0.57–1.13 (0.896)	1.1–0.55 (0.723)	PDMS
20.1	1.42	0.44	20.6	1.46	0.43	--	--	--	org.–org. (+PDMS)

## Supplementary Materials

The following are available online at https://www.mdpi.com/2073-4360/12/11/2476/s1, SI Figure S1: Spectroscopic evaluation of the completion of the hydrosilylation. SI Figure S2: FT-IR spectra of silanes and copolymers. SI Figure S3: Detail of the characteristic FT-IR peak of the Si-H bond stretching. SI Figure S4: 1H-NMR spectrum of the pure BAFKU mesogen with assigned peaks. SI Figure S5: 1H-NMR spectra of the precursors H03 and H11. SI Figure S6: 1H-NMR spectrum of the PDMS precursor H21. SI Figure S7: 1H-NMR spectra of copolymers H03–BAFKU2; H11–BAFKU2. SI Figure S8: 1H- NMR spectrum of the copolymer H21–BAFKU2. SI Figure S9: GPC traces of the prepared copolymers, overlaid. SI Figure S10: GPC trace of pure BAFKU. SI Figure S11: GPC trace of pure precursors H03, H11, H21. SI Figure S12: GPC trace of the copolymer H03–BAFKU2, H11–BAFKU2, H21–BAFKU. SI Figure S13: MALDI-TOF spectra: H03 and H03–BAFKU2. SI Figure S14: MALDI-TOF spectra: H11 and H11–BAFKU2. SI Figure S15: MALDI-TOF spectra: H21 and H21–BAFKU2. SI Figure S16: neat BAFKU: Temperature-dependent PLM intensity. SI Figure S17: H03–BAFKU2: Temperature-depend ent PLM intensity. SI Figure S18: H11: Temperature-dependent PLM intensity. SI Figure S19: H21–BAFKU2: Temperature-dependent PLM intensity. SI Figure S20: High-resolution textures of neat BAFKU: top: at 21 °C, bottom: at 61 °C, both in heating run. SI Figure S21: High-resolution textures of neat BAFKU: top: at 62 °C (heating run), bottom: at 39 °C (cooling run). SI Figure S22: High-resolution textures of H03–BAFKU2: top: at 10 °C, bottom: at 20 °C, both in heating run. SI Figure S23: High-resolution textures of H03–BAFKU2: top: at 46 °C, bottom: at 51 °C, both in heating run. SI Figure S24: High-resolution textures of H03–BAFKU2: top: at 45 °C, bottom: at 20 °C, both in cooling run. SI Figure S25: High-res H11–BAFKU2: top: at 10 °C, bottom: at 21 °C, both in heating run. SI Figure S26: High-resolution textures of H11–BAFKU2: top: high detail at 29 °C, bottom: at 22 °C, both cooling run. SI Figure S27: High-resolution textures of H11–BAFKU2: very high detail at 29 °C. SI Figure S28: High-resolution textures of H21–BAFKU2: top: at 10 °C, bottom: at 21 °C, both in heating run. SI Figure S29: High-resolution textures of H21–BAFKU2: top: at 10 °C, bottom: at 21 °C, both in heating run. SI Figure S30: High-resolution textures of H21–BAFKU2: texture at −29 °C, end of cooling run. SI Table S1: Molecular parameters of the DMS precursor polymers determined by 1H-NMR. SI Table S2. Characterization of molecular mass.
