# Supplementary material for: Low-Temperature Meltable Elastomers Based on Linear Polydimethylsiloxane Chains Alpha, Omega-Terminated with Mesogenic Groups as Physical Crosslinkers: A Passive Smart Material with Potential as Viscoelastic Coupling. Part I: Synthesis and Phase Behavior
Source: Polymers (Basel). 2020 Oct 25;12(11):2476. doi: 10.3390/polym12112476 (PMC7693640; doi:10.3390/polym12112476)
Supplement: Supplementary file 1 [file polymers-12-02476-s001.pdf]

SUPPLEMENTARY INFORMATION

Low-temperature-meltable elastomers based on linear polydimethylsiloxane chains alpha,omega-terminated with mesogenic groups as physical crosslinker: A passive smart material with potential as viscoelastic coupling. Part I: Synthesis and phase behaviour

Sabina Horodecka[1,2], Adam Strachota[1]*, Beata Mossety-Leszczak[3], Beata Strachota, Miroslav Šlouf[1], Alexander Zhigunov[1], Michaela Vyroubalová[1], Dana Kaňková[1], Miloš Netopilík[1], Zuzana Walterová[1]

[1)] Institute of Macromolecular Chemistry, Czech Academy of Sciences,
Heyrovskeho nam. 2, CZ-162 06 Praha, Czech Republic

[2)] Faculty of Science, Charles University,
Albertov 6, CZ-128 00 Praha 2, Czech Republic

[3)] Faculty of Chemistry, Rzeszow University of Technology,
al. Powstancow Warszawy 6, PL-35-959 Rzeszow, Poland

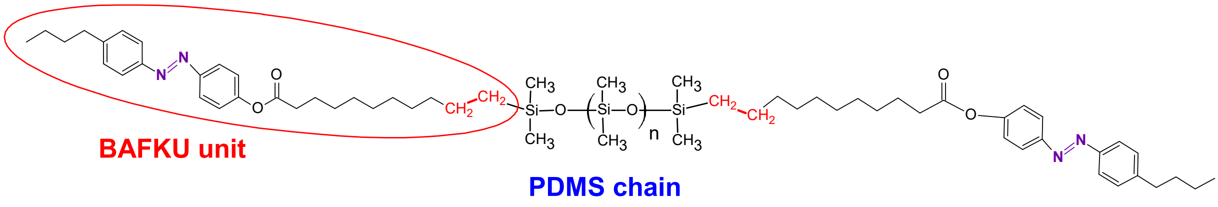

1. Synthesis and chemical microstructure

1.1. Evaluation of conversion of the PDMS during hydrosilylation reaction by means of FTIR

(a)

(b)

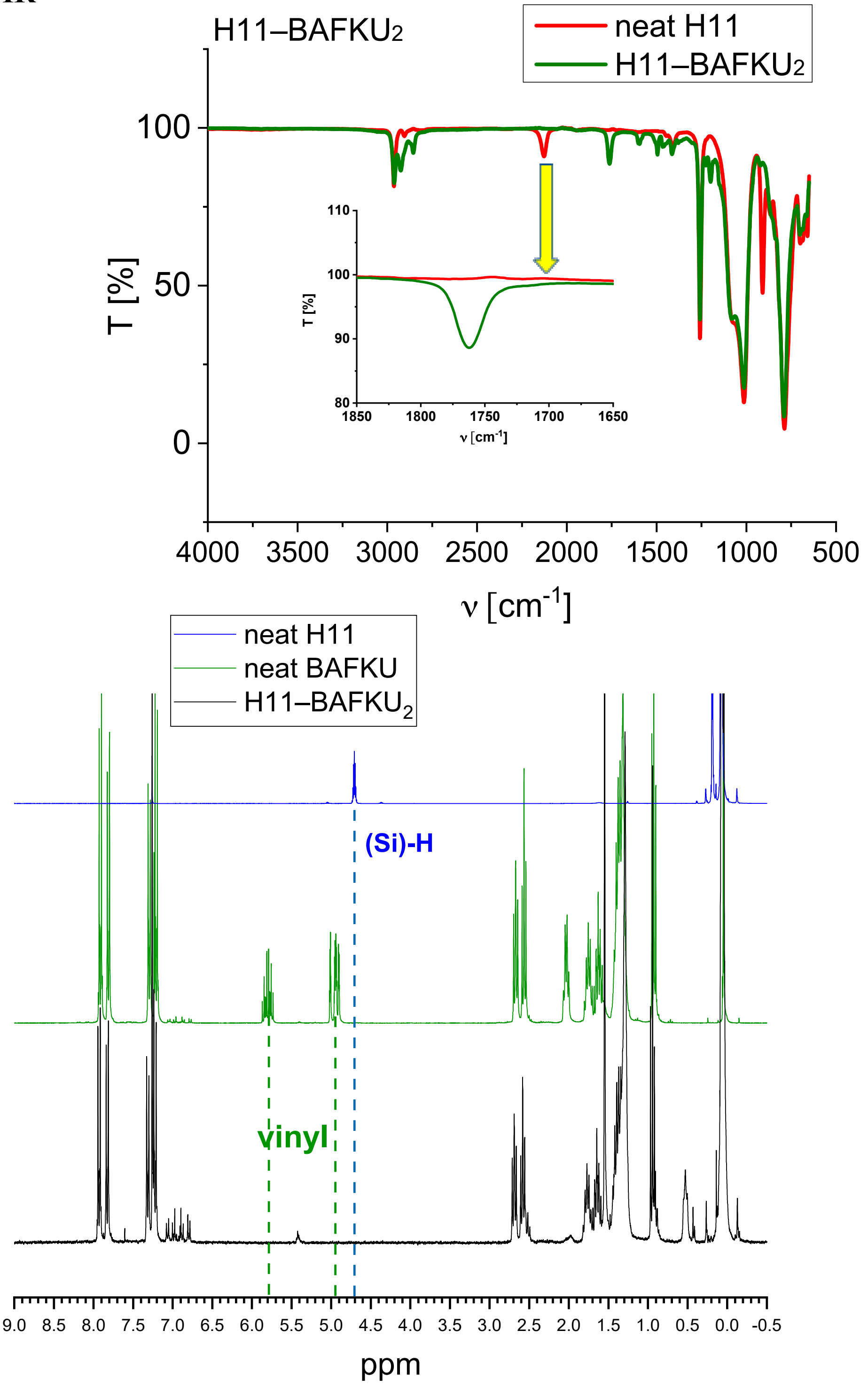

SI-Fig. 1: *Spectroscopic evaluation of the completion of the hydrosilylation reaction: (a): FTIR spectra of pure H11 and of H11–BAFKU₂ highlighting the disappearance of the Si-H as zoomed inlay; (b):* ^{1}*H-NMR of pure H11, BAFKU and H11–BAFKU₂ - details in* ***SI-Fig.*** *7.*

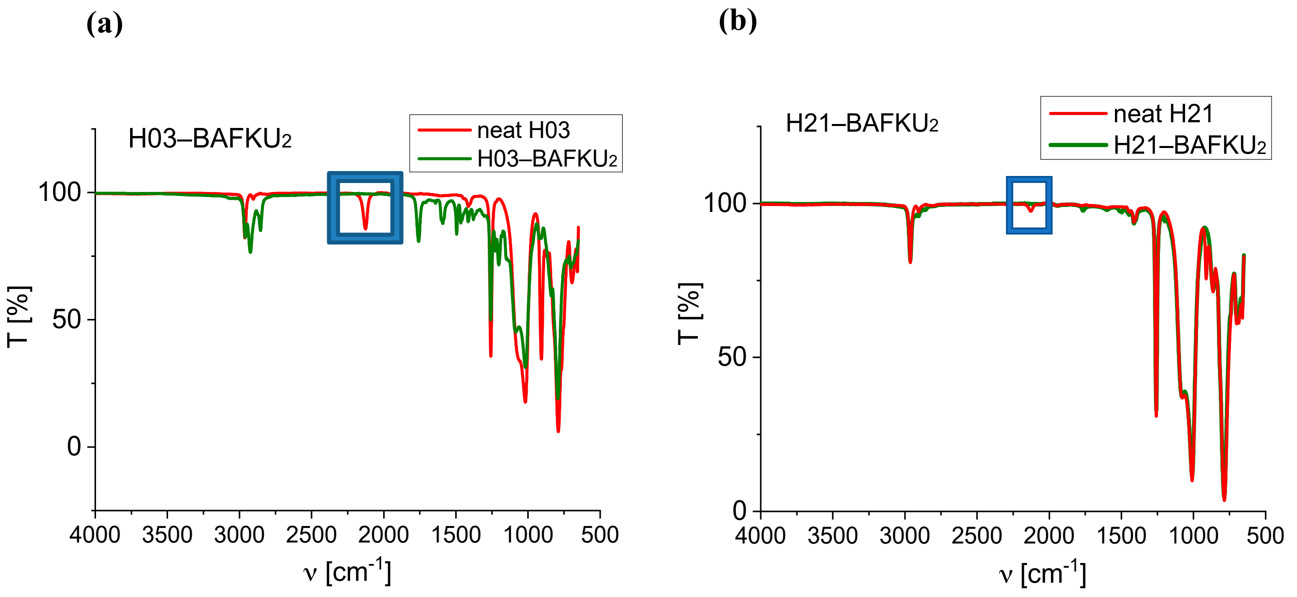

SI-Fig. 2: *FT-IR spectra of (a): pure H03 and of H03–BAFKU2; (b): pure H21 and of H21-BAFKU2; the characteristic peak of the Si-H bond stretching is highlighted.*

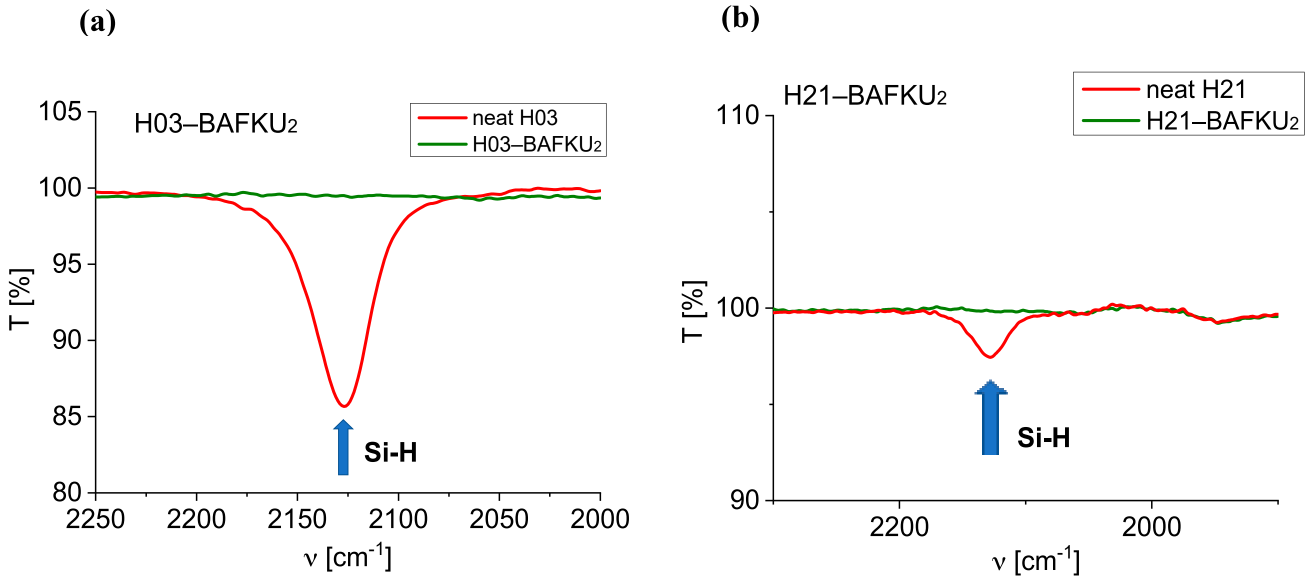

SI-Fig. 3: *Detail of the characteristic FT-IR peak of the Si-H bond stretching: (a): H03-BAFKU2 vs. H03; (b): H21–BAFKU2 vs. H21.*

1.2. ^{1}H-NMR: Purity of components and products, as well as synthesis conversion

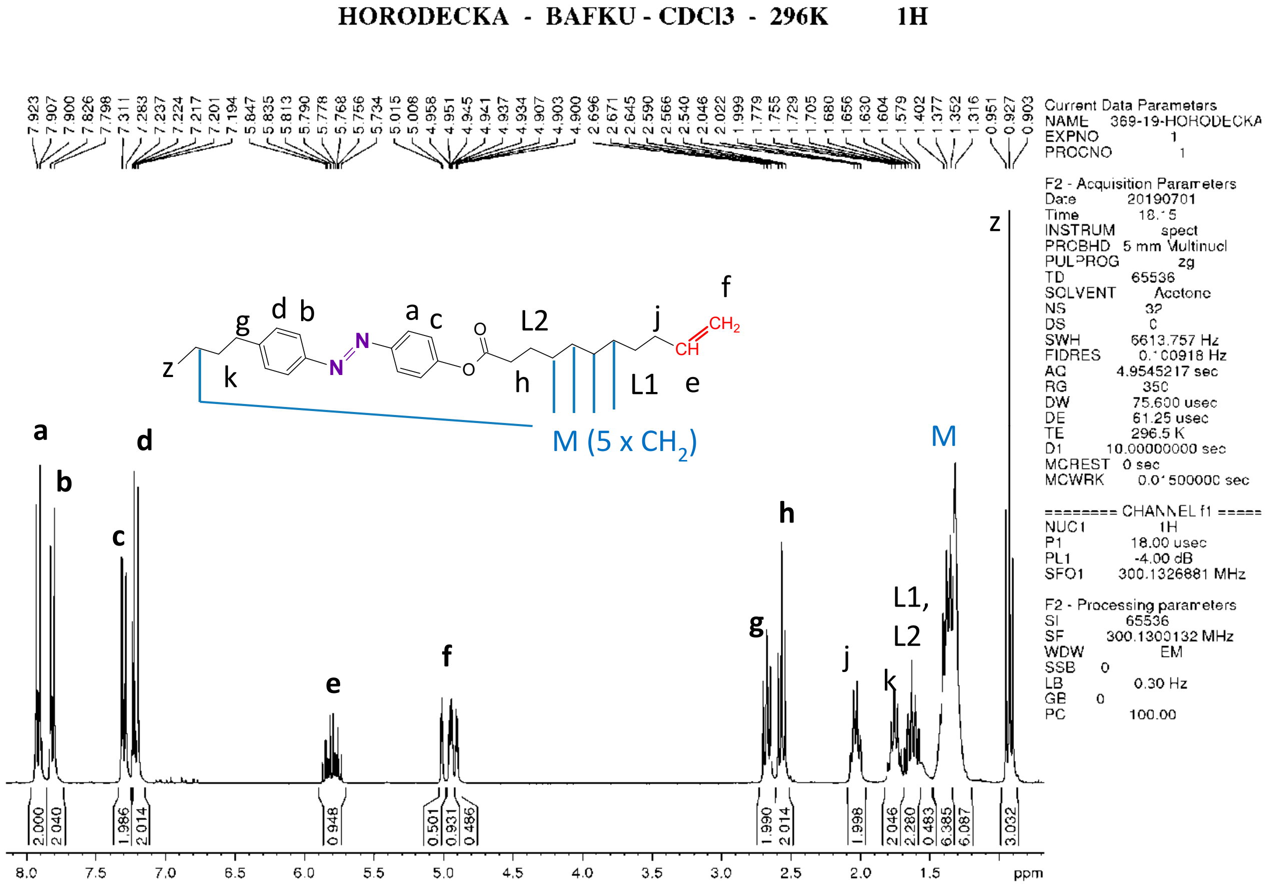

SI-Fig. 4: *^{1}H-NMR spectrum of the pure BAFKU mesogen with assigned peaks.*

^{1}H-NMR: analysis of the PDMS components

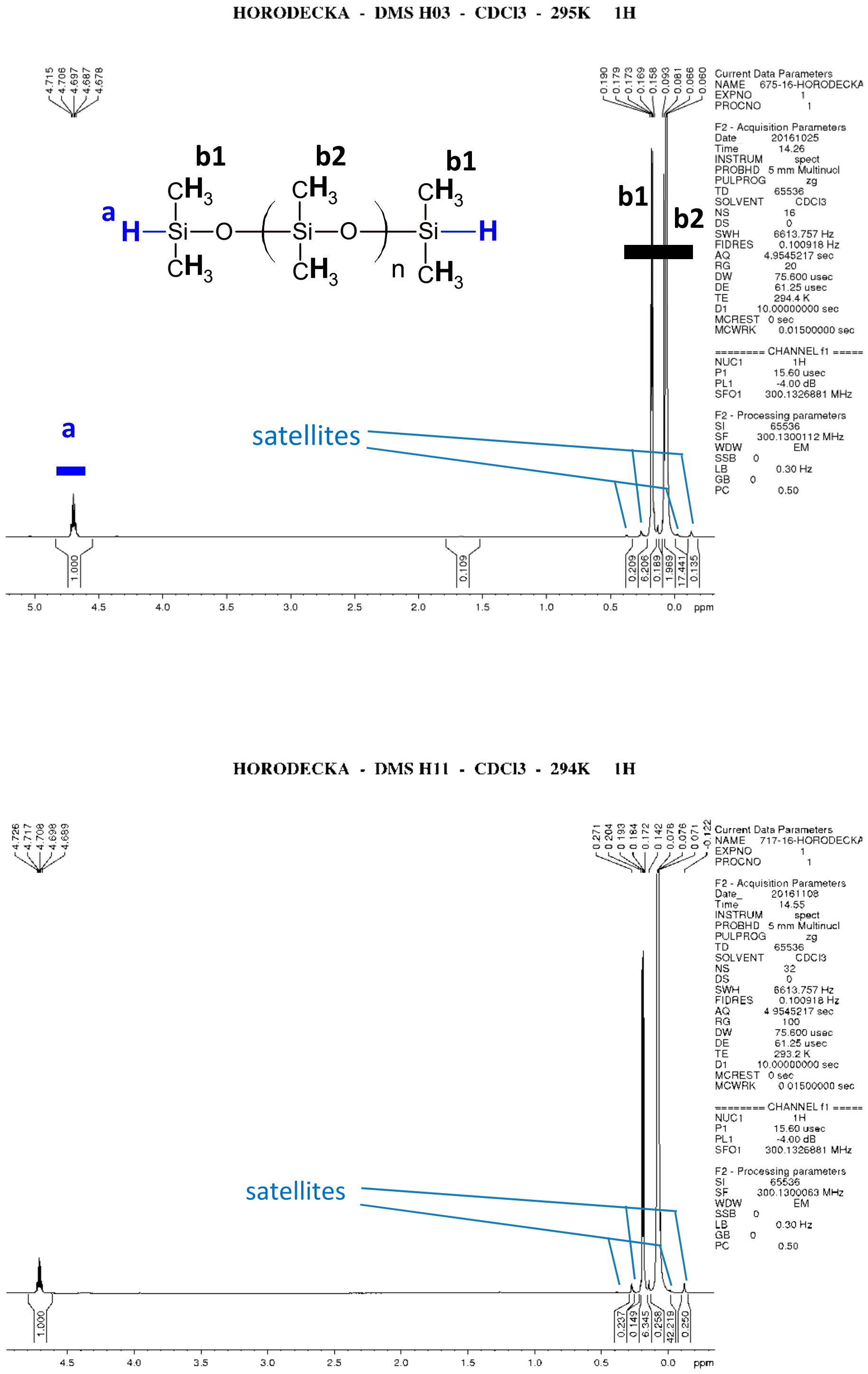

SI-Fig. 5: *^{1}H-NMR spectra of the PDMS precursors: (top): H03; (bottom): H11; the peak of the methyl groups is near 0.1 ppm, the Si-H peak near 4.70 ppm.*

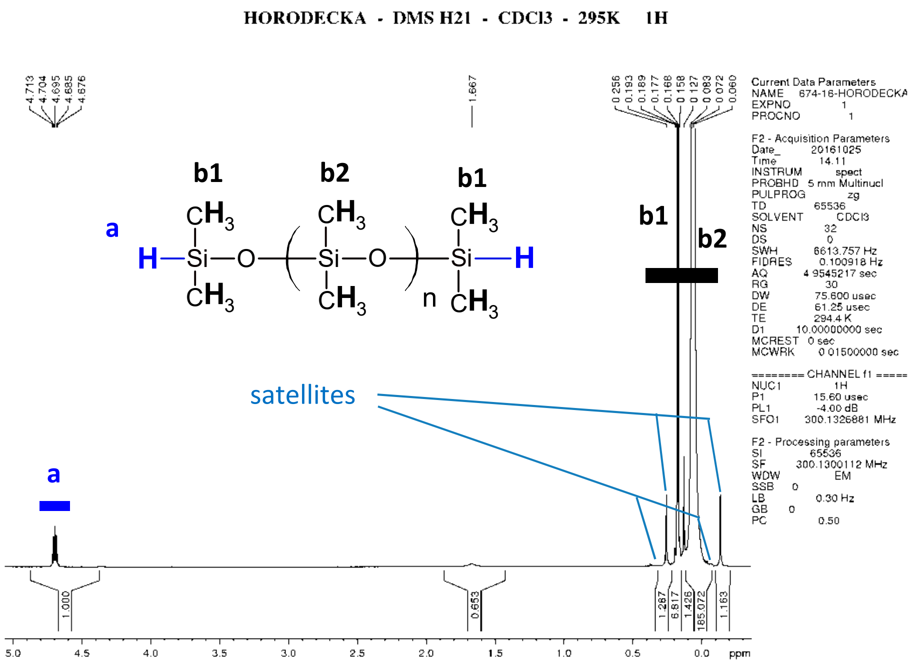

SI-Fig. 6: *^{1}H-NMR spectrum of the PDMS precursor H21; the peak of the methyl groups is near 0.1 ppm, the Si-H peak near 4.70 ppm.*

1.2.1. Structure parameters of “DMS” (PDMS) polymers from ^{1}H-NMR analysis

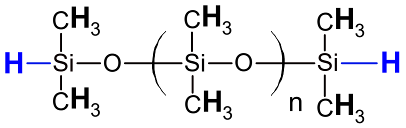

Aim:

calculation of **n (number of internal repeat units)**, coefficient from above structure formula, of the molecular mass of the whole molecule, as well as of the equivalent molecular mass per SiH function (***EqMW***); the latter is needed to calculate accurate reactant amounts for syntheses.

Ratios of signal integrals from the 1H-NMR spectra (***SI-Fig.*** *5 and* ***6***) in relation to the above structure formula:

**H** in **SiH** end groups	**H** in **CH_3** groups in all siloxane repeat units
2	(2*3)*n
if the SiH integral is normalized to 1.0, the following integral ratios are obtained:	
1	3*n

Magnitudes for calculation:

n: coefficient from above structure formula (number of internal repeat units)

m = n + 2 : number of all siloxane repeat units

RU = molecular mass of an internal repeat unit (of the block $[(CH_3)_2SiO]$)
= 74.154 g/mol

E = molecular mass of both end groups taken together, hence of $(CH_3)_2(H)Si\text{-}O\text{-}Si(H)(CH_3)_2$
= 134.325 g/mol

M = molecular mass of the whole “DMS” molecule (as shown in the above structure formula)

*Obtaining **n** from the ^{1}H-NMR spectra in **SI-Fig. 5** and **6***:

-at first, the integrals in the spectra are normalized so, that the value of the integral of the SiH peak is equal to 1.00 (as done in ***SI-Fig.*** *5 and* ***6***); it is then assigned 1 = ‘*SiH_norm*’; each molecule contains two hydrogens from SiH groups;

-next, the sum of all integrals of the peaks of $(CH_3)_2Si$< groups, '*CH3*' is calculated; each siloxane repeat unit in the DMS molecule contains 6 hydrogens from two methyl groups; as result of the above normalization of the SiH signal, an integral value of 3 corresponds to one siloxane repeat unit; the normalized value '*CH3_norm*' is calculated by dividing '*CH3*' by three;

-finally, the coefficients **m** (number of all Si-containing units) and **n** (number of internal siloxane units) are calculated, using '*CH3_norm*' and '*SiH_norm*':

m = *CH3_norm* / *SiH_norm* = *CH3_norm* / 1 = *CH3_norm* = '*CH3'* / 3

hence **m = '*CH3'* / 3**

and **n = m – 2**

M = E + n*RU (with molecular masses of the constants E, RU taken from above)

EqMW = M/2

Results:

SI-Table 1: *Molecular parameters of the PDMS precursor polymers as determined by ^{1}H-NMR, compared with the data given by the manufacturer (last two columns, italics).*

**name of siloxane precursor**	**'CH_3' from NMR**	***m*** (number of all repeat units) **from NMR**	***n*** (number of internal repeat units) **from NMR**	**M_n** (= 'M') **from NMR** **[g/mol]**	**EqMW = M_n/2** **[g/mol]**	**M_n** (supplier) **[g/mol]**	***m*** (supplier, calculated)
H03	25.805	8.602	6.602	623.865	311.9325	*450±50*	*6.257*
H11	48.971	16.324	14.324	1196.482	598.241	*1 050±50*	*14.348*
H21	193.315	64.438	62.438	4764.377	2382.189	*4 500±500*	*60.873*

1.2.2. Copolymers PDMS–BAFKU$_2$: ^{1}H-NMR analysis

The ^{1}H-NMR spectra of the copolymers are shown in ***SI-Fig. 7–8***. In ***SI-Fig. 7***, the assignment of the peaks is shown on the example of H03–BAFKU$_2$. It can be seen that the characteristic peaks of the BAFKU units are preserved, except for the vinyl groups (atoms “e”, “f”), which disappeared completely (the signals of “e”, “f” are newly found among the alkyl groups). From the PDMS precursors, the SiH peaks also quantitatively disappeared, while the signals of the methyl groups persist. The disappearance of the vinyl- and SiH-signals was mentioned above as a proof of quantitative conversion of accurately stoichiometric amounts of the copolymer components. The integral of the peak of the methyl groups of PDMS (near 0.06 ppm) is ‘reinforced’ by the intensity of former terminal methylene units of the vinyl groups of BAFKU.

If an isolated signal of BAFKU is taken as ‘end group signal’, e.g. the one of the aromatic H atoms “a” at 7.93 ppm, then it can be compared with the intensity of the methyl signals of the PDMS repeat units. If the signal “a” is calibrated to 2.00, then the integrals of the PDMS methyl groups should be approximately the same like in case of the PDMS precursors (there are 4 hydrogens “a” per copolymer molecule, while there were only 2 SiH hydrogens, hence the value of the calibration). In order to obtain the intensity of the ‘original’ methyl groups of the PDMS precursor, the value of the integral of the methyl groups of PDMS has to be corrected by subtracting 2.00: under the given calibration, this corresponds to 4 H atoms of the terminal CH_2 units from BAFKU which now are bonded to Si atoms (“f” in ***SI-Fig. 7 top***). The corrected integral then should be identical with ‘CH3’ from ***SI-Table 1***, while the number of repeat units m = ‘CH3’/3 should be identical. The results can be seen below:

**copolymer name**	***‘CH$_3$’* from NMR**	***corrected ‘CH$_3$’***	***m*** (number of all repeat units) **according to NMR**	expected ***m*** according to precursor	***deviation*** (expressed as excess of PDMS) **[%]**
H03–BAFKU$_2$	27.443	25.443	8.481	8.602	-1.4
H11–BAFKU$_2$	57.342	55.342	18.447	16.324	+13.0
H21–BAFKU$_2$	243.869	241.869	81.290	64.438	+26.1

It can be observed, that the end-group analysis (numbers of the repeat units of PDMS, **m**) yields nearly identical results in case of the shortest PDMS chain (H03), while in case of the longer chains, an increasing excess of PDMS seemingly is observed. This result rather can be attributed to the very different relaxation dynamics of the highly mobile PDMS chains and of the rigid BAFKU units, which gain influence with increasing chain length. The seeming excess of PDMS (up to 26%) namely is not accompanied by the appearance of a residual SiH peak, which should be expected in such a case. In view of the missing signals of the functional groups of the reactants, and of the probable highly different relaxation behaviour of the molecular segments, the most accurate ^{1}H-NMR-derived molecular masses of the copolymers appear to be sums of NMR-determined M_n of PDMS precursors (from ***SI-Table 1***) plus two masses of the BAFKU units. This calculation was used in the respective column in ***SI-Table 2*** as M_n from ^{1}H-NMR.

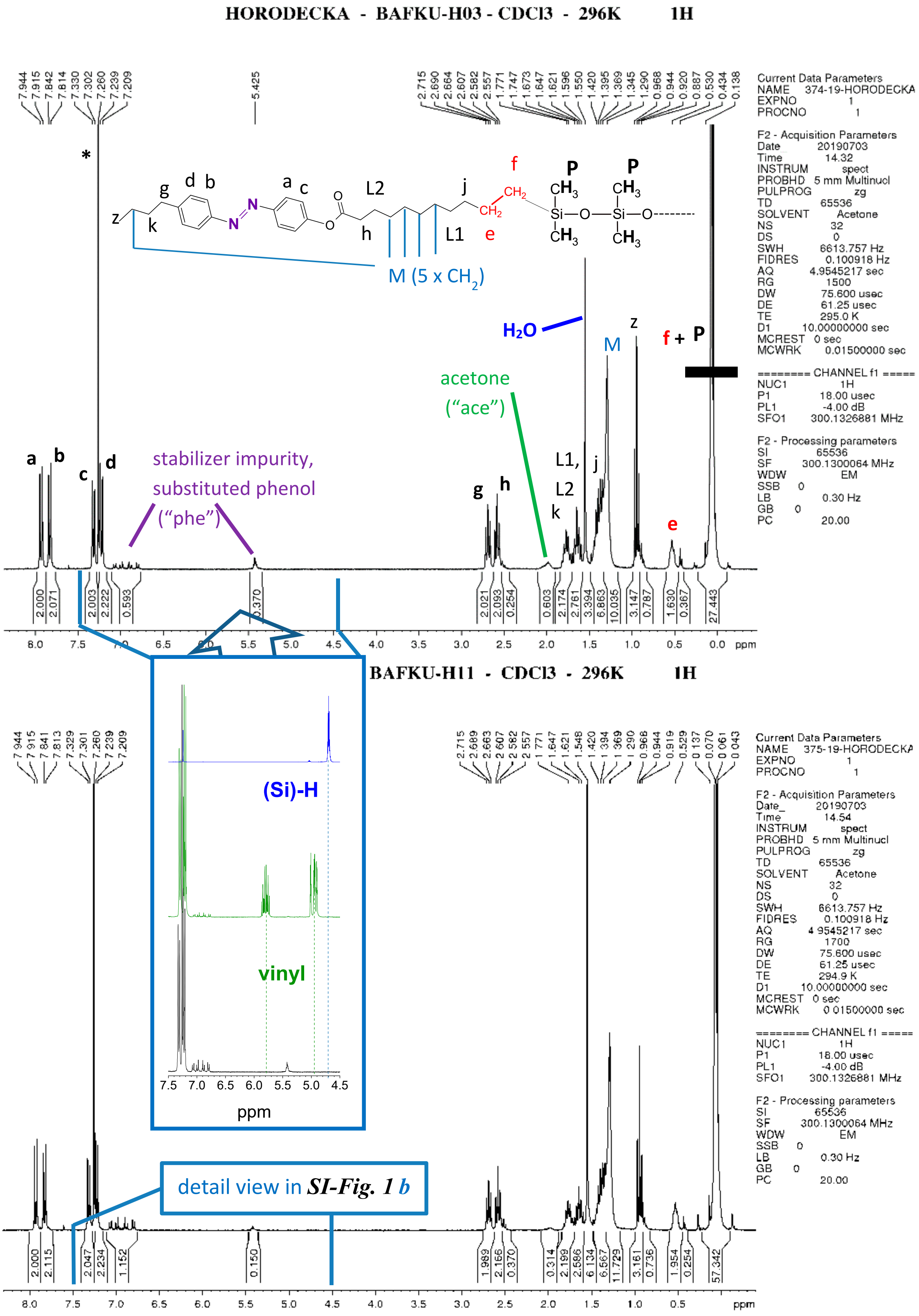

SI-Fig. 7: *^{1}H-NMR spectra of the copolymers (top): H03–BAFKU₂; (bottom): H11–BAFKU₂.*

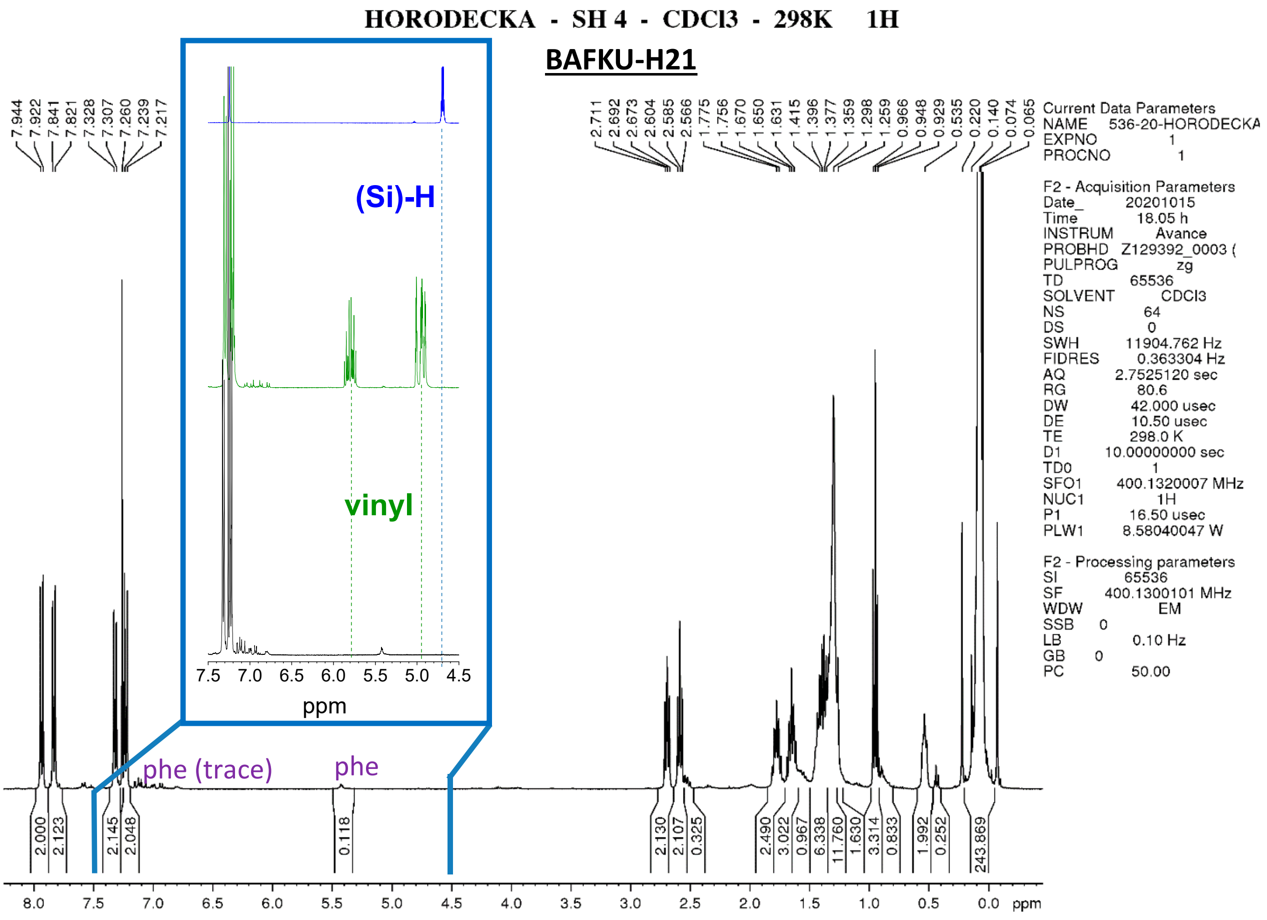

SI-Fig. 8: *^{1}H-NMR spectrum of the copolymer H21–BAFKU$_2$.*

1.3. Molecular mass analysis of the copolymers, as well as of the precursors

The molecular masses of the prepared copolymers were verified using several methods, namely SEC (GPC), MALDI-TOF and ^{1}H-NMR (equipment and setup: see Experimental Part of the main Manuscript). If the results (***SI-Table 2***) obtained by NMR, GPC and MALDI-TOF are compared, some differences can be clearly seen:

The ^{1}H-NMR spectroscopy (see spectra of copolymers: ***SI-Fig. 7*** and ***8***, reactants: ***SI-Fig. 4–6***) proved itself as the most accurate, albeit indirect method for numeric average molecular mass determination, as will be explained below: The characteristic groups of 'hydrido-PDMS' precursor polymers generate well-separated and sufficiently distinct signals in all the studied cases, namely the signals of SiH end-groups near 4.70 ppm and the signals of the PDMS methyl groups at 0.18–0.06 ppm, which make possible a fairly exact determination of the ratio of the two terminal repeat units to the remaining ones (see assignment of peaks in ***SI-Fig. 5***). In this way, the average length of the PDMS precursor is determined (see ***SI-Table 1***). In this way, the exact molecular masses of the precursors were determined prior to the syntheses, as described above, in order to use precisely stoichiometric reactant ratios. In case of the copolymers, the nearly quantitative conversion of the constituent components BAFKU and PDMS prepolymers was subsequently confirmed by the disappearance of the vinyl and SiH signals, which otherwise persist in case of non-stoichiometric or un-reacted mixtures. If the end-group analysis was carried out after the hydrosilylation coupling – which was carried out at precise stoichiometry and with no residues of reactive groups were observed after the synthesis – then the end group analysis (aromatic protons of mesogen vs. protons of PDMS methyl groups, as described above) yielded satisfactory results in case of the shortest PDMS chain (H03): identical PDMS chain length of precursor and final copolymer was then obtained from the evaluation of integrals. In case of the longer precursors, differences in relaxation behaviour led to over-estimated PDMS content in the copolymer spectra (but no residual SiH signals), as was discussed above (in the same samples, GPC indicated contrarily a small excess of BAFKU). Hence, the most precise determination of molecular weight (M_n) of the prepared copolymers by ^{1}H-NMR was achieved, if two masses of BAFKU were added to one mass of the PDMS precursor determined by ^{1}H-NMR prior to the synthesis, under the condition of confirmed absence of un-reacted functional groups via post-synthesis ^{1}H-NMR.

The SEC (GPC) results are illustrated by ***SI-Fig. 9*** (see also ***SI-Table 2***; source data are shown in ***SI-Fig. 10–12***): The GPC traces of copolymers are compared with BAFKU and with the PDMS precursors, and all are shown as traces in the ELSD (evaporative-light-scattering) detector, while for the copolymers, also the UV/Vis trace (refraction index (at λ = 264 nm) detector) also is shown. GPC yields fairly realistic results (if compared to the indirect NMR method which was considered most accurate) for products with molecular weights in

the range of 4 to 6 kDa. For molecular masses of 2 kDa and smaller, the accuracy of GPC was already poor (over-estimated by +50%). The addition of the BAFKU end groups to the mass of the whole molecule is best visible by GPC in case of the mid-sized H11–BAFKU$_2$. The difference between the NMR and GPC results seems to partly originate in the different molecular masses of repeat units of polystyrene (calibration standard), and of PDMS and BAFKU. Moreover, the coiling/uncoiling tendency of the product molecules is affected by the aggregation tendency of BAFKU, which also could cause the formation of associates of several molecules under favourable conditions. Similar effects in GPC were also observed by the authors on distantly related PDMS copolymers with BAFKU in a previous work **[1]**.

The GPC traces in ***SI-Fig. 9*** suggest the presence of some amount of non-bonded BAFKU in the final product, ca 5% of the integral area – if the more accurate ELSD detector is used. In case of the more biased refraction-index-detector (UV light, 264 nm), the residual BAFKU peak amounts to up to 25% of integral area. Comparison with NMR (***SI-Fig. 1b***, invisible vinyl groups of free BAFKU, nor Si-H from PDMS), however, indicates that the amount of free BAFKU is greatly over-estimated by the UV detector, and possibly somewhat over-estimated by the ELSD detector. At least in case of the short copolymer H03–BAFKU$_2$, a 5 wt.% excess of BAFKU should be well visible as vinyl signals, and also as discrepance in the ratio aromatic / PDMS signals. The strong bias of the UV detector (264 nm) can be explained by a relatively strong UV/Vis absorption of azobenzene and hence of BAFKU near 250 nm, while the relative proximity of the ELSD laser wavelength (658 nm) to the long-wave absorption peaks of azobenzene between 400–500 nm also could generate some over-estimation.

The MALDI-TOF results are listed in ***SI-Table 2*** while the mass spectra are shown in ***SI-Fig. 13–15***. In case of MALDI-TOF, good results are obtained for small or mid-sized and reasonably stable molecules like neat BAFKU, or the copolymers H03–BAFKU$_2$ and H11–BAFKU$_2$. In case of the larger macromolecules like H21–BAFKU$_2$ and neat H21, their fragmentation or vaporization of low-molecular fractions dominates and the MALDI-TOF results highly under-estimate the molecular mass. In case of the small H03 (neat) molecule, a doubled molecular mass is observed, most likely due to condensation of SiH end-groups during vaporisation. This latter effect is suppressed in H11 and H21, due to the 'dilution' of the end groups.

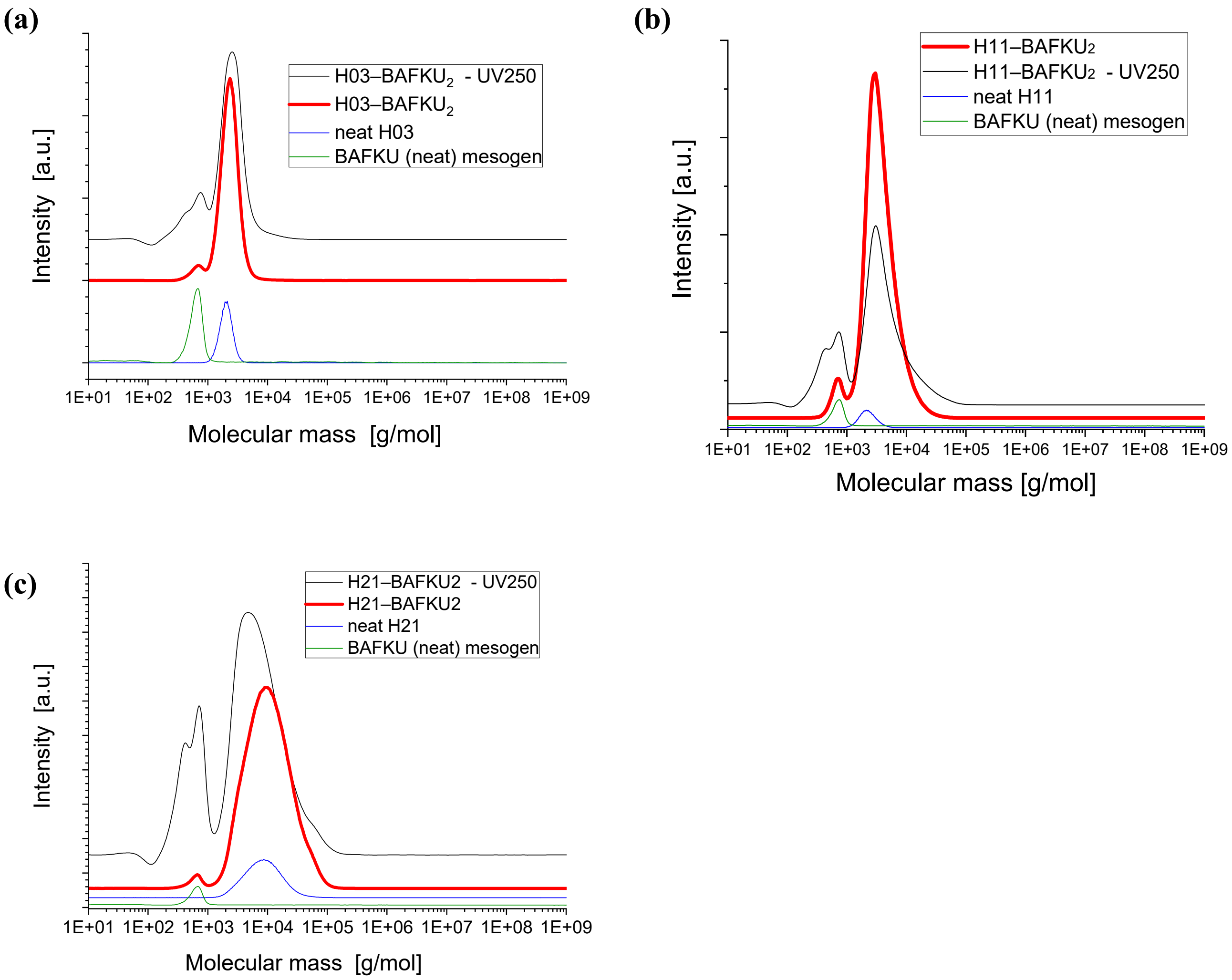

SI-Fig. 9: *GPC traces of the prepared copolymers, overlaid with the traces of their respective PDMS precursors and of the BAFKU mesogen: (a): H03–BAFKU₂; (b): H11–BAFKU₂; (c): H21–BAFKU₂; all samples were analysed using the ELSD detector, while the copolymers were additionally analysed also using the UV-refraction index (at λ = 264 nm) detector, as noted in the legend.*

SI-Table 2. *Characterization of molecular mass of the prepared copolymers, as obtained by GPC, ¹H-NMR and MALDI-TOF; data obtained for the precursors are also listed for comparison.*

Sample	GPC: Peak maximum [g/mol]	GPC: M_n from automatic evaluation [g/mol]	GPC: PD (autom.) [ ]	M_n from producer [g/mol]	M_n by ¹H-NMR [g/mol]	M_n by MALDI-TOF [g/mol]
**BAFKU**	732	654	1.07	420.587	n.a.	421
**H03**	1 759	1 595	1.11	450±50	624	1250 (!)
**H03–BAFKU$_2$**	2 371	1 693	1.11	1 391	1 465	1 442
**H11**	2 157	2 105	1.12	1 050±50	1 196	1 368
**H11–BAFKU$_2$**	3 008	3 161	1.40	1 891	2 037	1 886
**H21**	7 367	5 710	1.63	4 500±500	4 764	1 590 (!)
**H21–BAFKU$_2$**	9 605	7 819	1.93	5 341	5 605	2 331 (!)

1.3.1. GPC source data

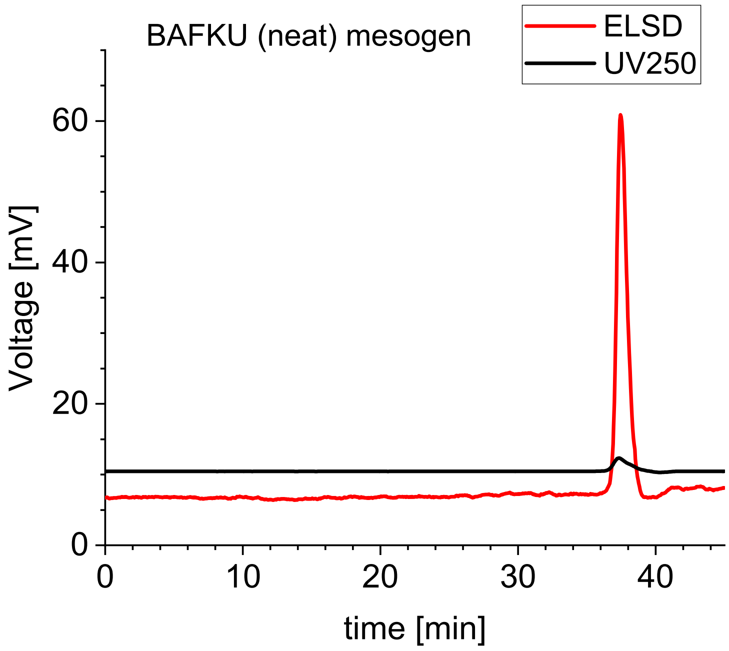

BAFKU

nr.	Max. RT	Start RT	End RT	flow rate correction	Mp	Mn	Mw	Mz	PD	I [mV]	I [%]
x	37.45	34.67	39.45	1	750	654	703	769	1.074924	54.41	100

SI-Fig. 10: *GPC trace of pure BAFKU, the trace of the UV-vis detector is given in mV and not magnified.*

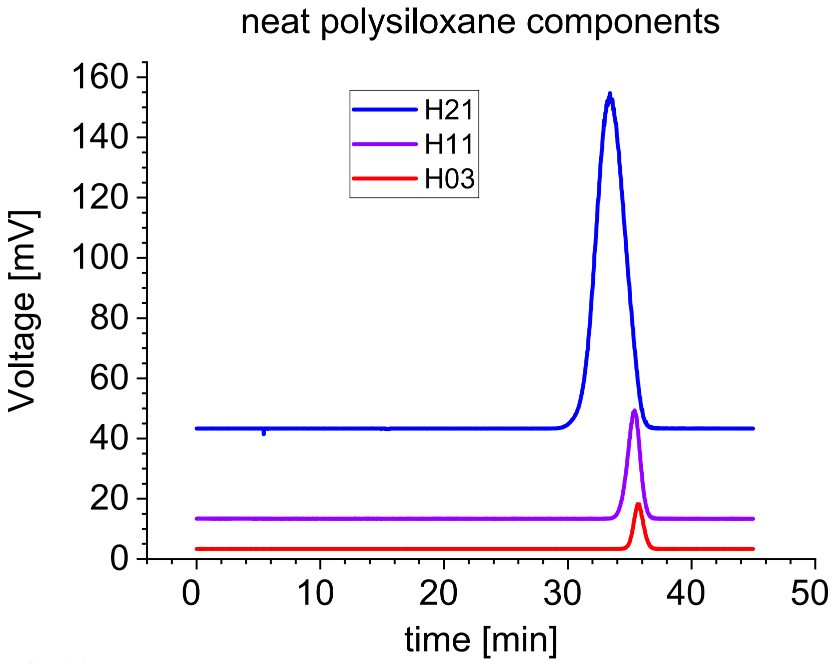

DMSH03

nr.	Max. RT	Start RT	End RT	flow rate correction	Mp	Mn	Mw	Mz	PD	I [mV]	I [%]
x	35.65	32.27	40.87	1	1801	1595	1771	1951	1.1102	15.10	100

DMSH11

nr.	Max. RT	Start RT	End RT	flow rate correction	Mp	Mn	Mw	Mz	PD	I [mV]	I [%]
x	35.42	32.38	38.58	1	2091	2105	2356	2661	1.1193	36.29	100

DMSH21

nr.	Max. RT	Start RT	End RT	flow rate correction	Mp	Mn	Mw	Mz	PD	I [mV]	I [%]
x	33.41	27.92	40.04	1	7471	5710	9322	15792	1.6327	111.81	100

SI-Fig. 11: *GPC traces of pure polydimethylsiloxane precursors H03, H11 and H21, as detected by ELSD.*

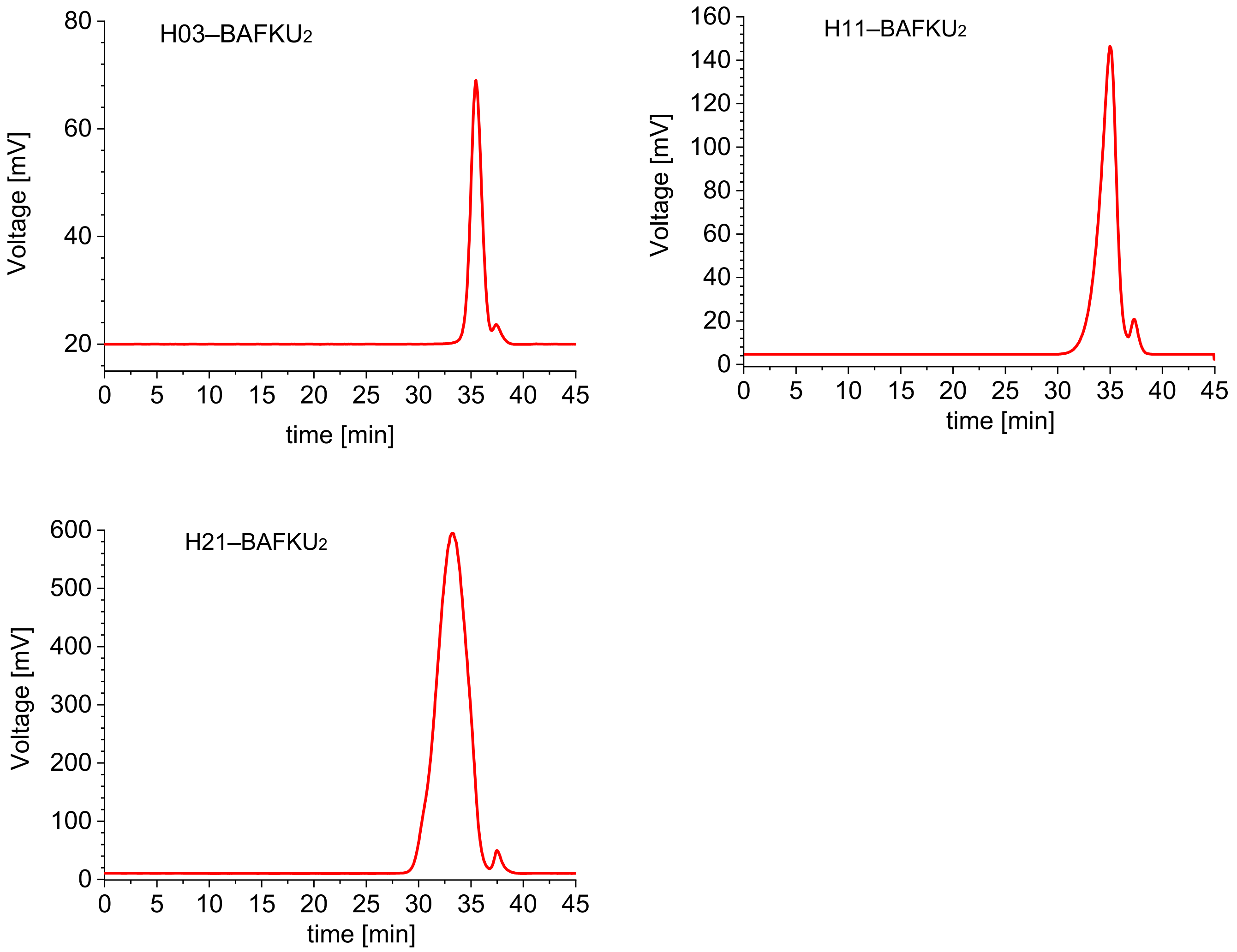

SI-Fig. 12: *GPC traces of the copolymers H03–BAFKU$_2$, H11–BAFKU$_2$ and H21–BAFKU$_2$, as detected by the more accurate ELSD detector; small peaks of un-reacted neat BAFKU are visible to the left from the main peak: in view of the* ^{1}*H-NMR results, the BAFKU peaks appear to be somewhat over-estimated also by the ELSD detection.*

Data from ***SI-Fig. 12***:

H03–BAFKU$_2$

nr.	Max. RT	Start RT	End RT	flow rate correction	Mp	Mn	Mw	Mz	PD	I [mV]	I [%]
1	35.49	33.61	36.87	1	1825	1693	1882	2097	1.1121	735.04	93.19
x	37.45	36.87	39.34	1	525	475	510	542	1.0741	53.72	5.66

H11–BAFKU$_2$

nr.	Max. RT	Start RT	End RT	flow rate correction	Mp	Mn	Mw	Mz	PD	I [mV]	I [%]
**1**	35.25	31.02	36.85	1	2698	3046	3905	5492	1.2822	515.28	94.75
**x**	37.50	36.95	38.62	1	663	603	623	642	1.0327	28.54	5.25

H21–BAFKU$_2$

nr.	Max. RT	Start RT	End RT	flow rate correction	Mp	Mn	Mw	Mz	PD	I [mV]	I [%]
1	33.27	28.45	36.63	1	9941	7819	15076	29164	1.9283	585.65	93.65
x	37.50	36.63	39.85	1	729	636	697	754	1.0967	39.69	6.35

1.3.2. MALDI-TOF spectra

H03–BAFKU$_2$

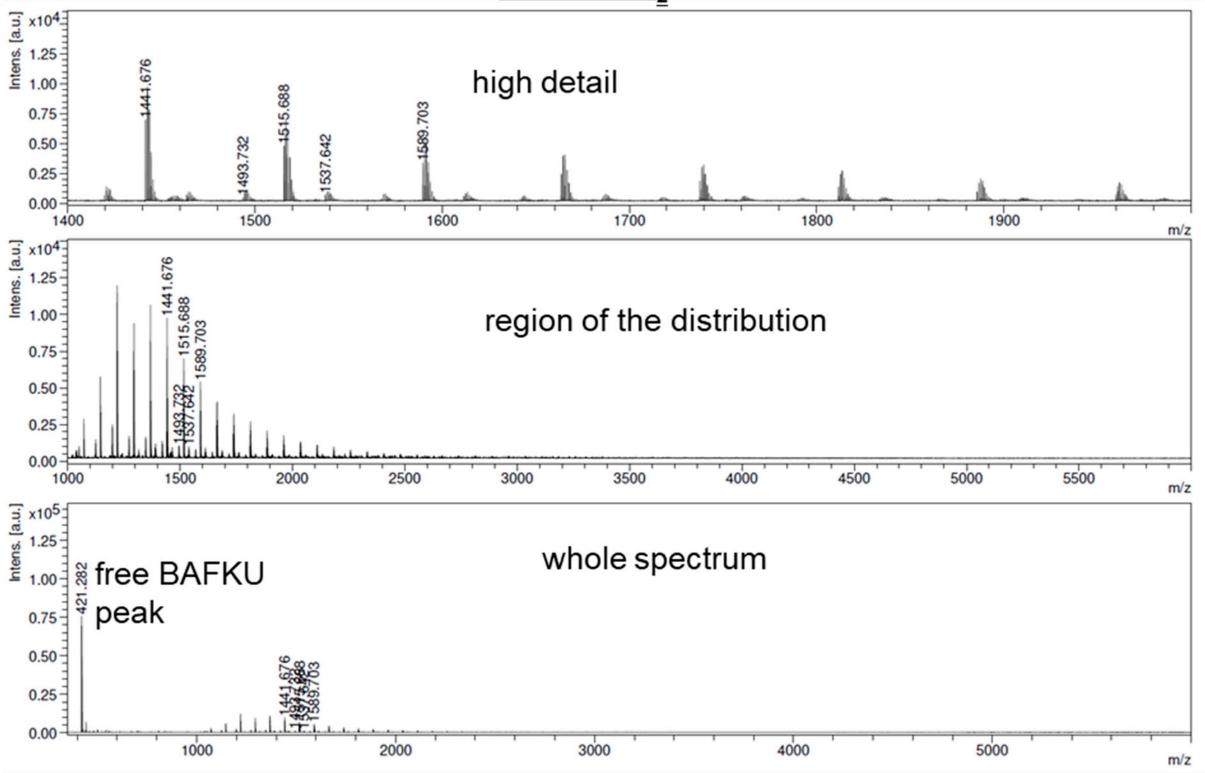

H03–BAFKU$_2$

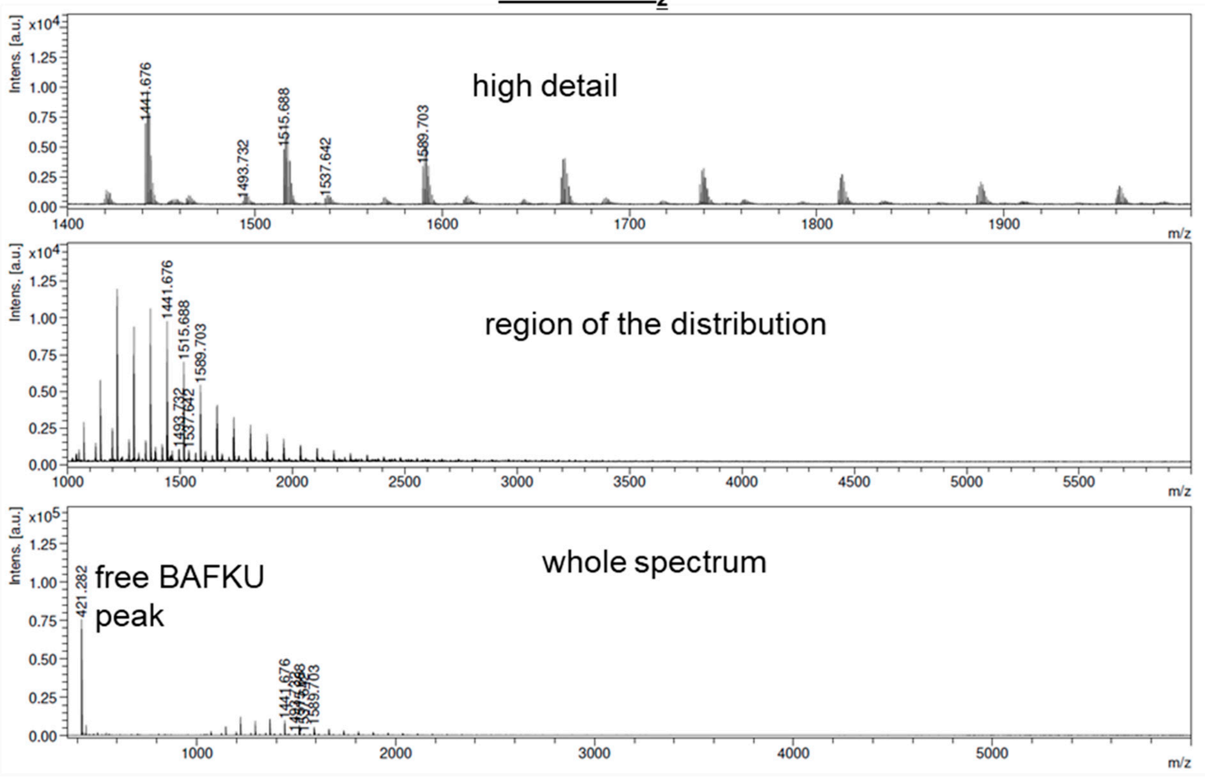

SI-Fig. 13: *MALDI-TOF spectra: H03–BAFKU$_2$ (top) vs. neat H03 (bottom).*

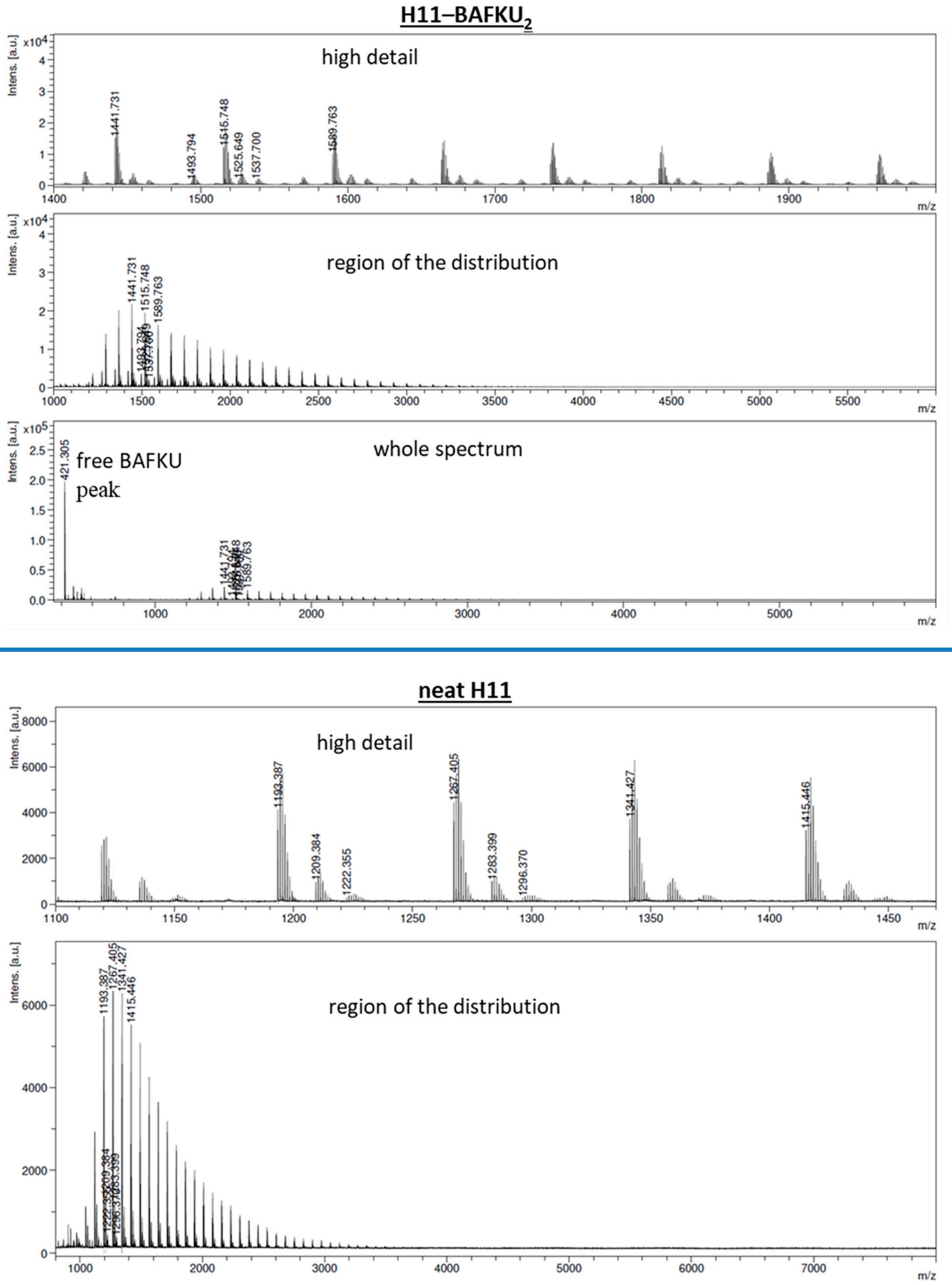

SI-Fig. 14: *MALDI-TOF spectra: H11–BAFKU2 (top) vs. neat H11 (bottom).*

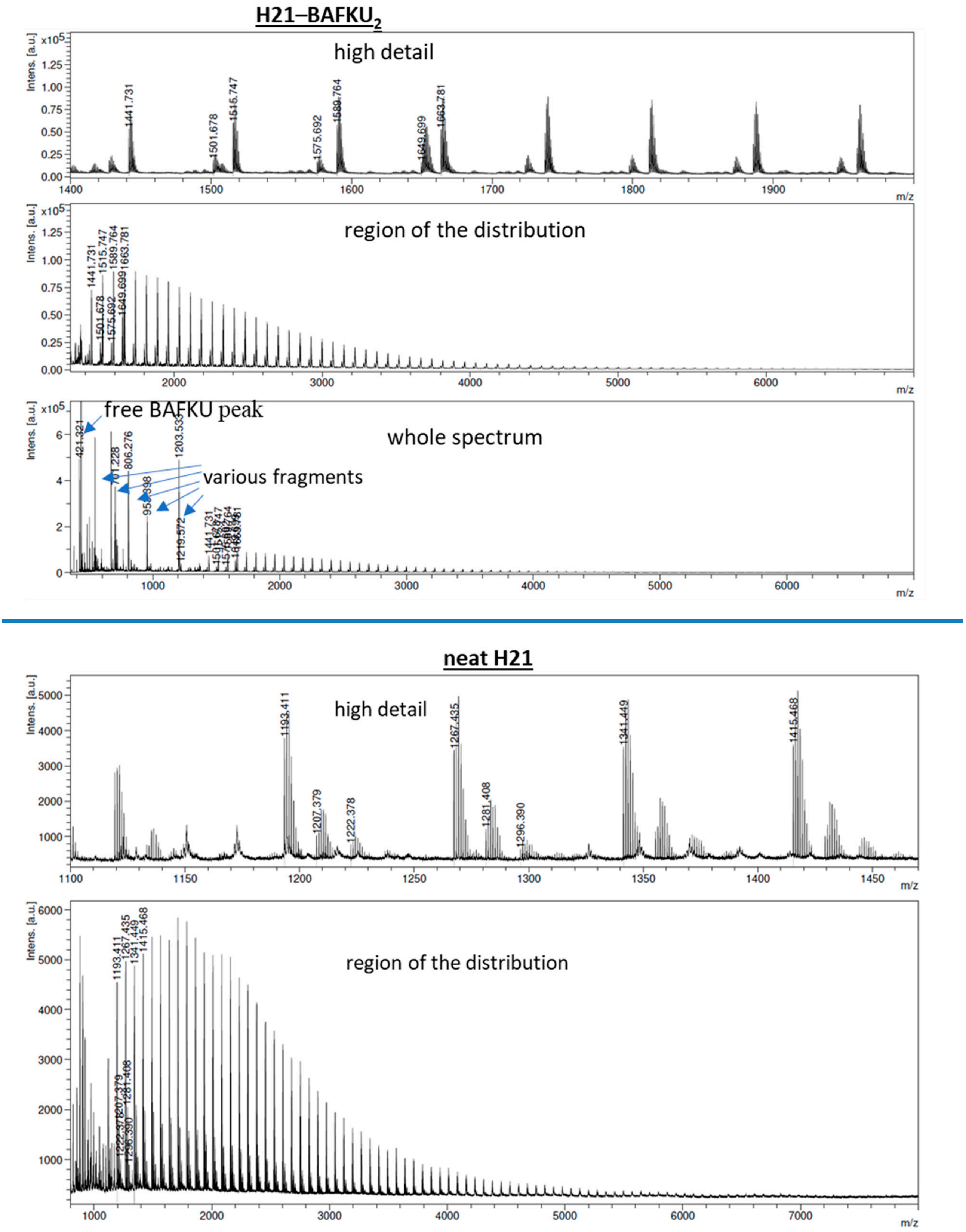

SI-Fig. 15: *MALDI-TOF spectra: H21–BAFKU2 (top) vs. neat H21 (bottom).*

1.4. Polarized light microscopy (PLM)

PLM intensity: neat BAFKU2

(a) **(b)**

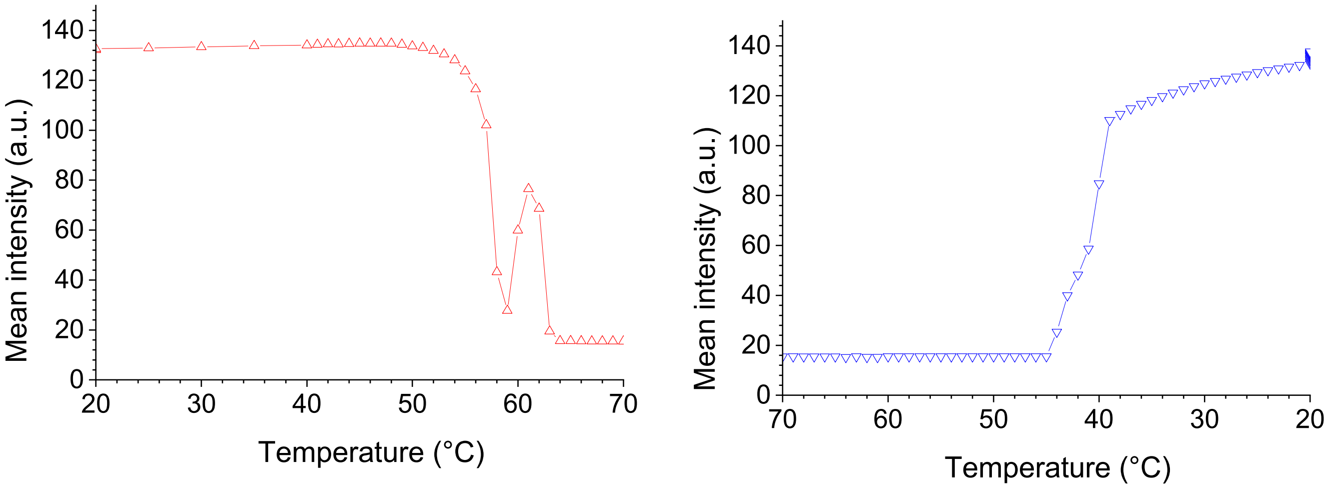

SI-Fig. 16: *Temperature-dependence of the average polarized light intensity ('black and white mode') calculated from the whole image frames taken during the PLM investigation of* ***neat BAFKU****: (a) heating run from 20 to 70°C; (b) cooling run from 70 to 20°C.*

PLM intensity: H03-BAFKU2

(a) **(b)**

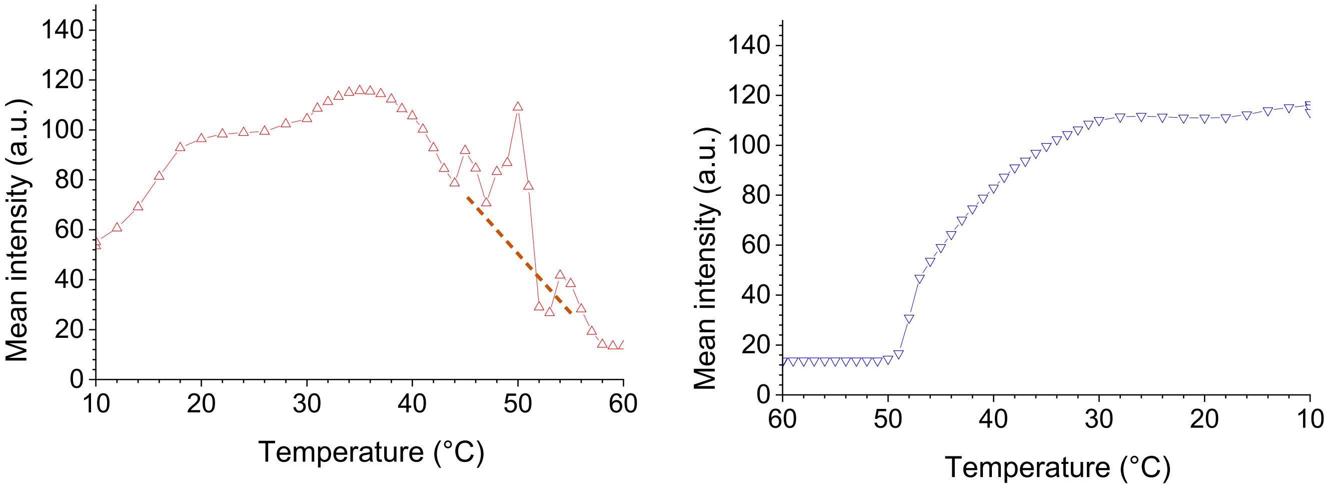

SI-Fig. 17: *Temperature-dependence of the average polarized light intensity ('black and white mode') calculated from the whole image frames taken during the PLM investigation of* ***H03-BAFKU$_2$*** *: (a) heating run from 10 to 60°C; (b) cooling run from 60 to 10°C.*

PLM intensity: H11-BAFKU2

(a)

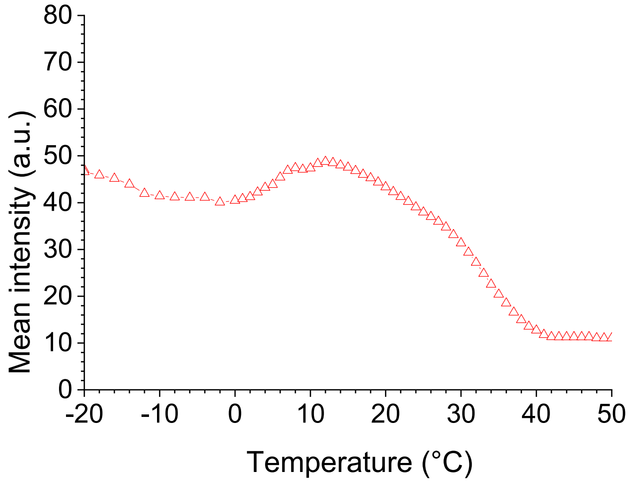

(b)

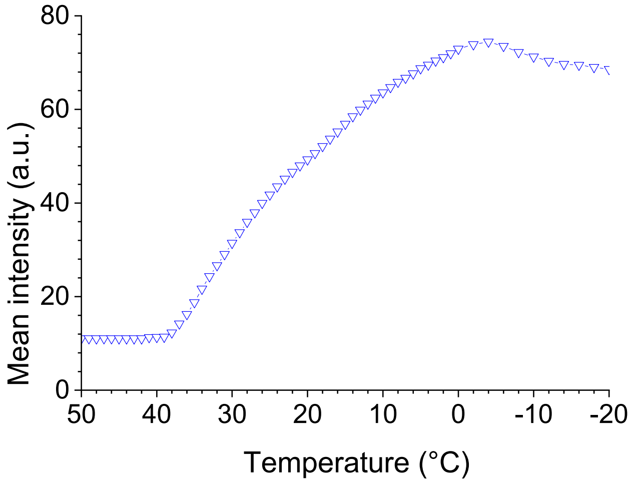

SI-Fig. 18: *Temperature-dependence of the average polarized light intensity ('black and white mode') calculated from the whole image frames taken during the PLM investigation of* ***H11-BAFKU$_2$*** *: (a) heating run from -20 to 50°C; (b) cooling run from 50 to -20°C.*

PLM intensity: H21-BAFKU2

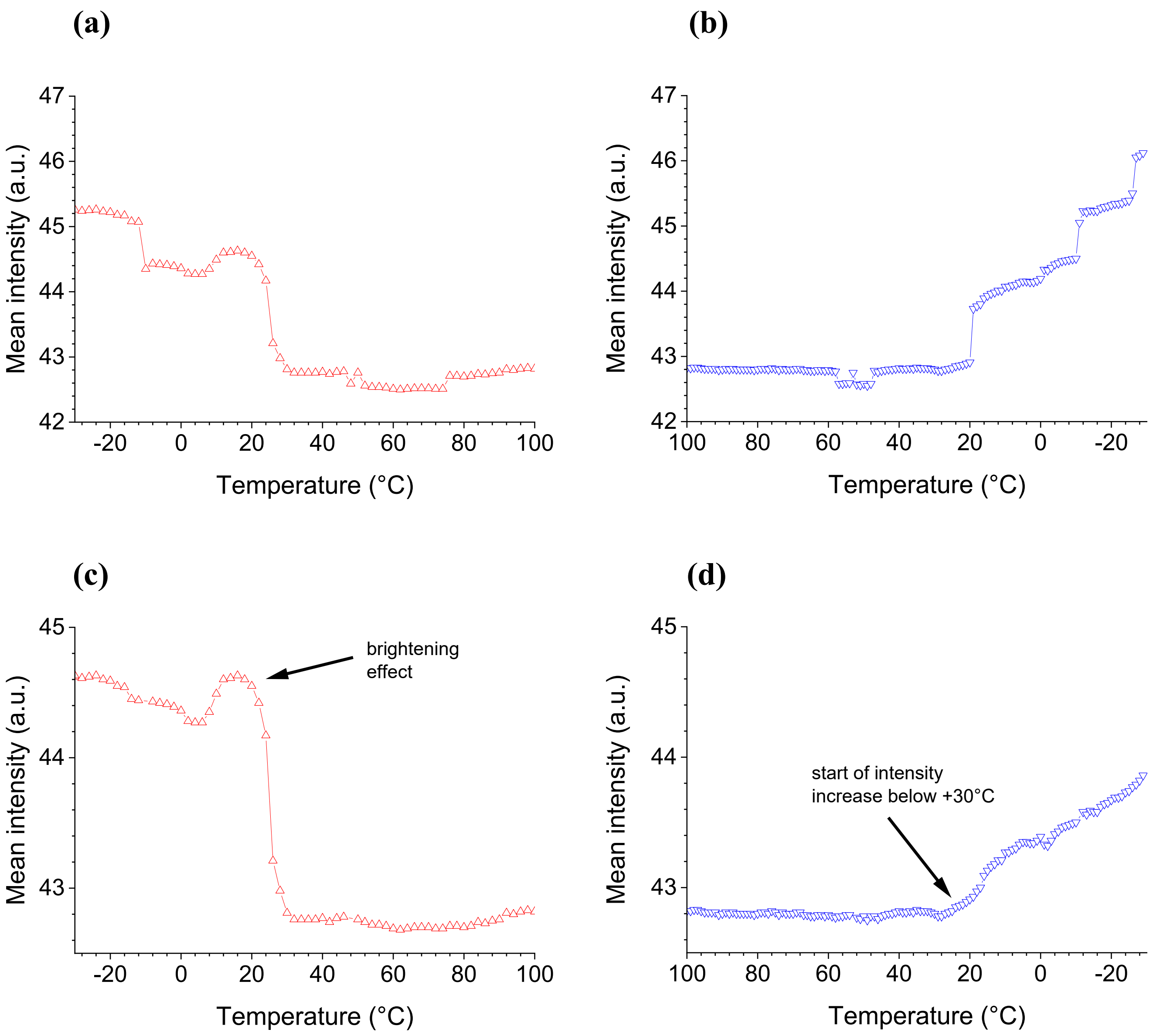

SI-Fig. 19: *Temperature-dependence of the average polarized light intensity ('black and white mode') calculated from the whole image frames taken during the PLM investigation of* ***H21–BAFKU$_2$*** *: (a, c) heating run from -30 to 100°C; (b, d) cooling run from 100 to -30°C; (a, b) original data; (c, d) data with corrected effect of change of focusing.*

1.4.1. Details of PLM textures of neat BAFKU

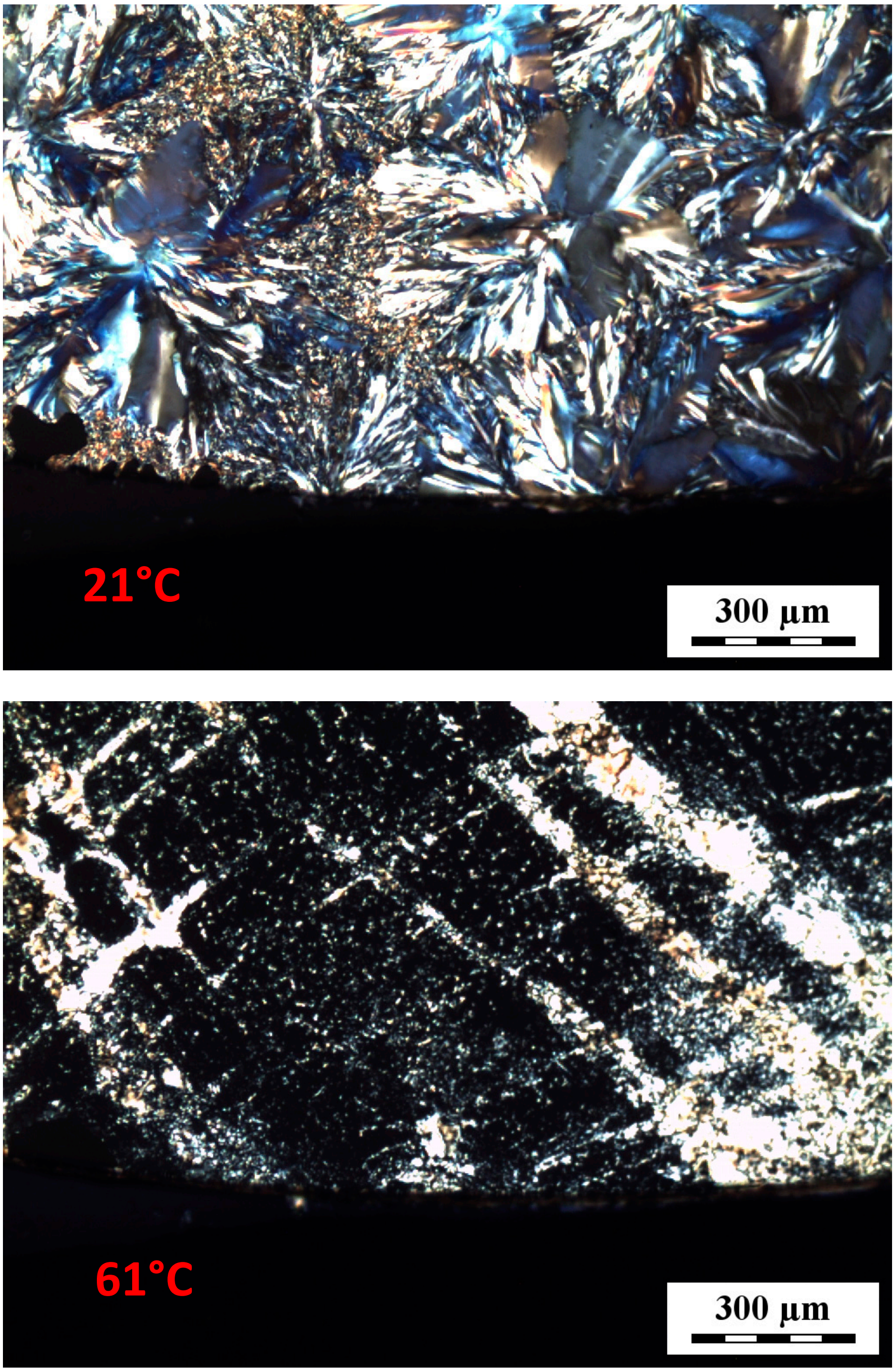

SI-Fig. 20: *High-resolution images of textures of neat BAFKU: top: 21°C, bottom: 61°C, both in heating run.*

Neat BAFKU

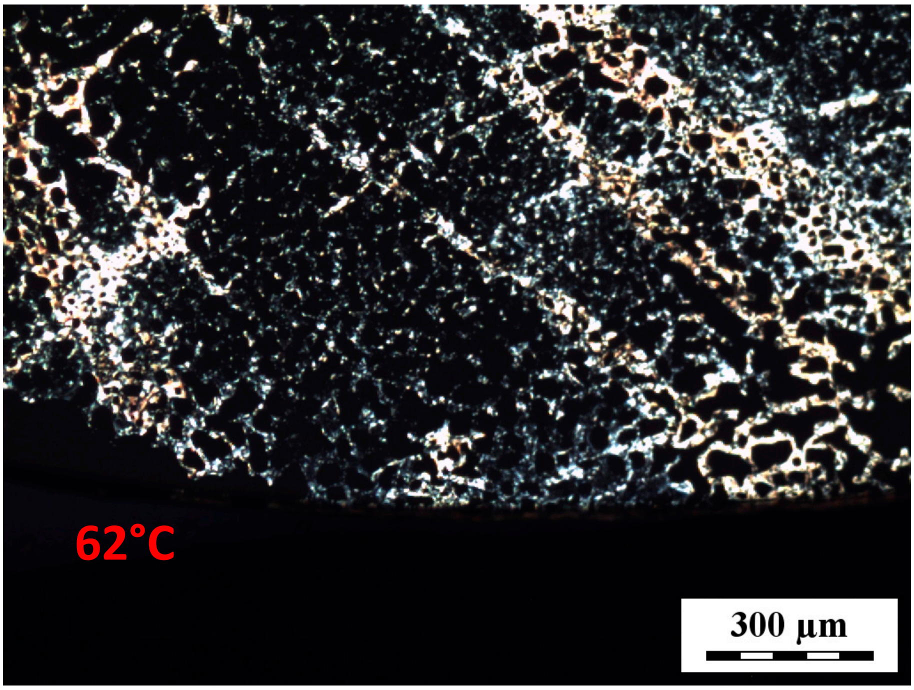

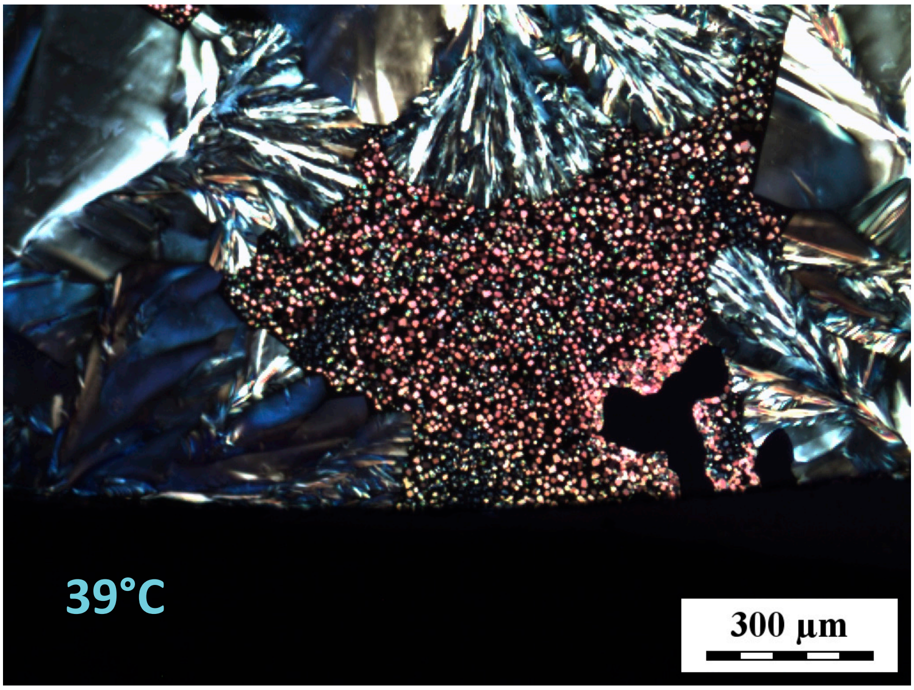

SI-Fig. 21: *High-resolution images of textures of neat BAFKU: top: 62°C (heating run), bottom: 39°C (cooling run).*

1.4.2. Details of PLM textures: H03–BAFKU$_2$

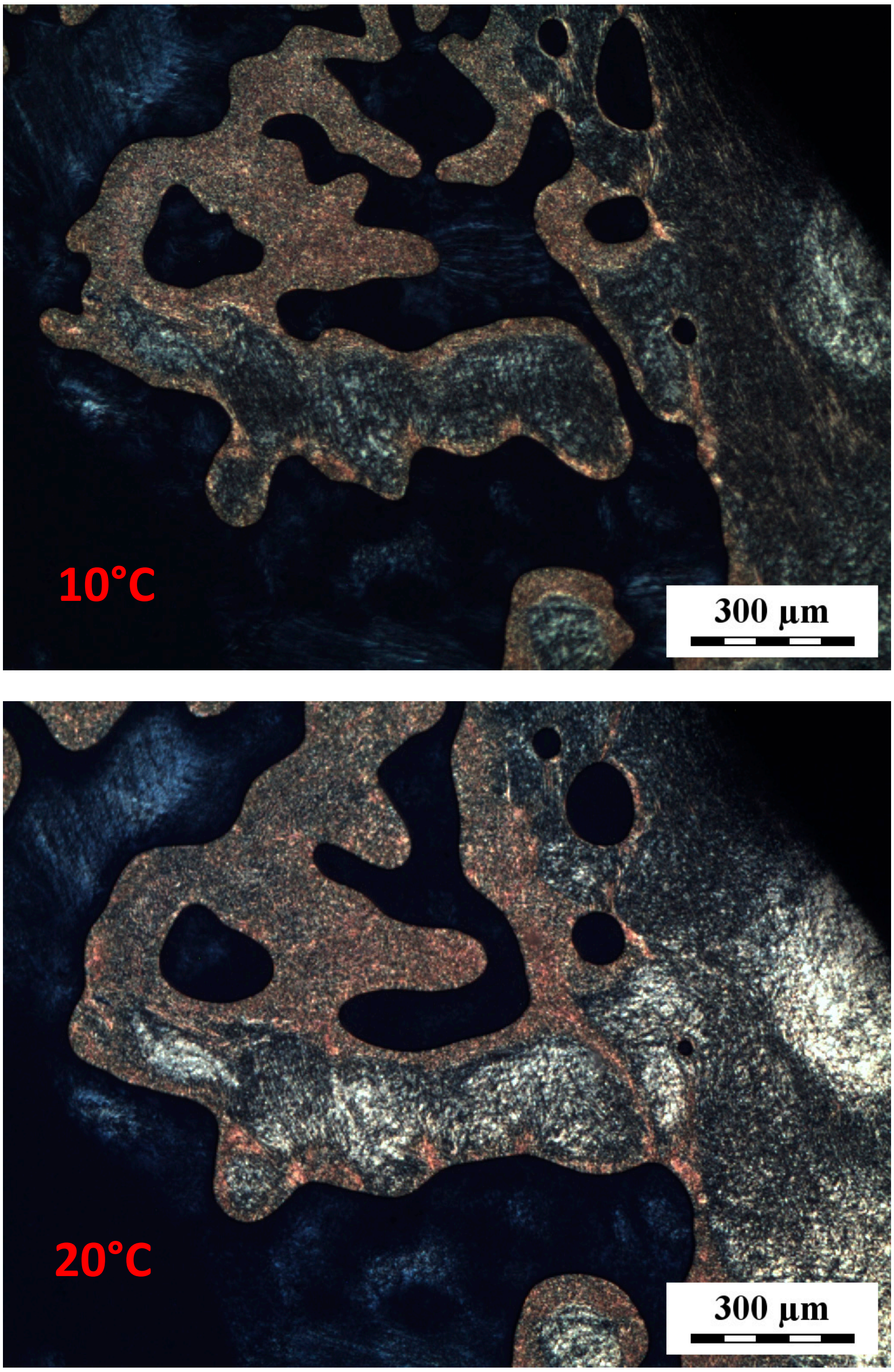

SI-Fig. 22: *High-resolution images of textures of H03–BAFKU$_2$: top: 10°C, bottom: 20°C, both in heating run.*

H03–BAFKU$_2$

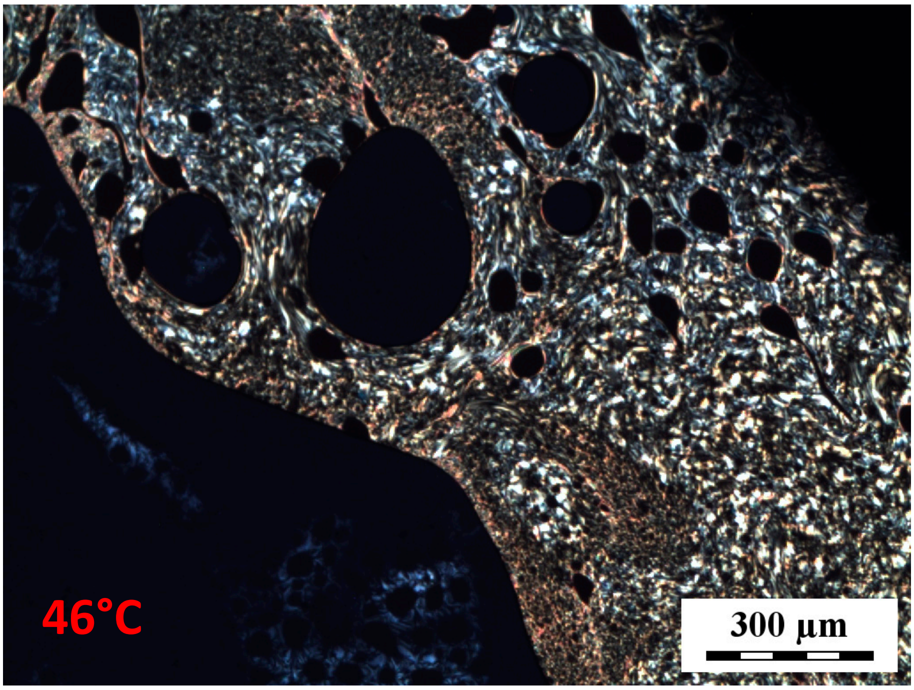

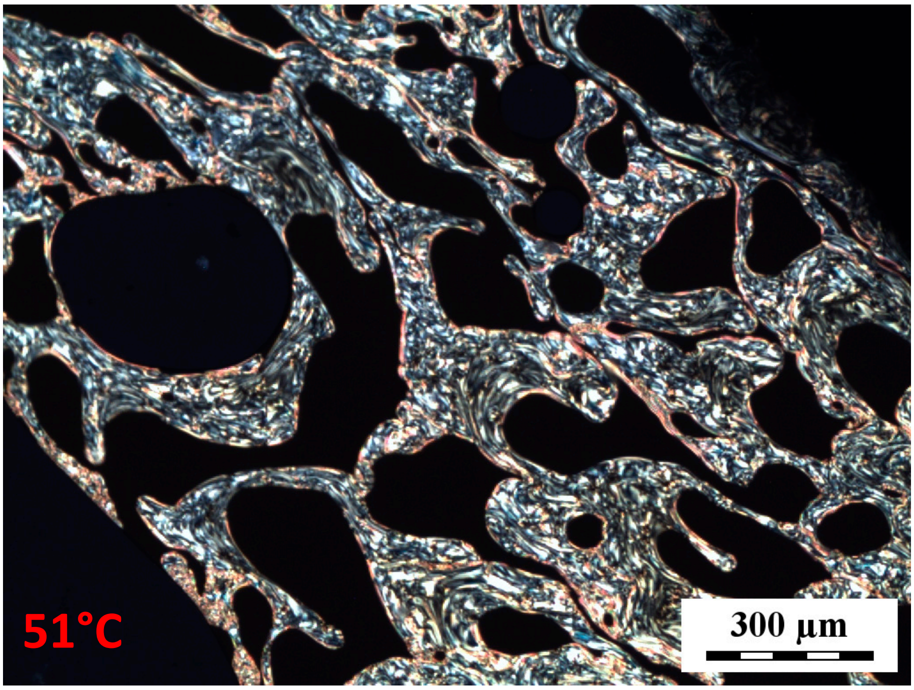

SI-Fig. 23: *High-resolution images of textures of* ***H03–BAFKU$_2$****: top: 46°C, bottom: 51°C, both in heating run.*

<u>H03–BAFKU$_2$</u>

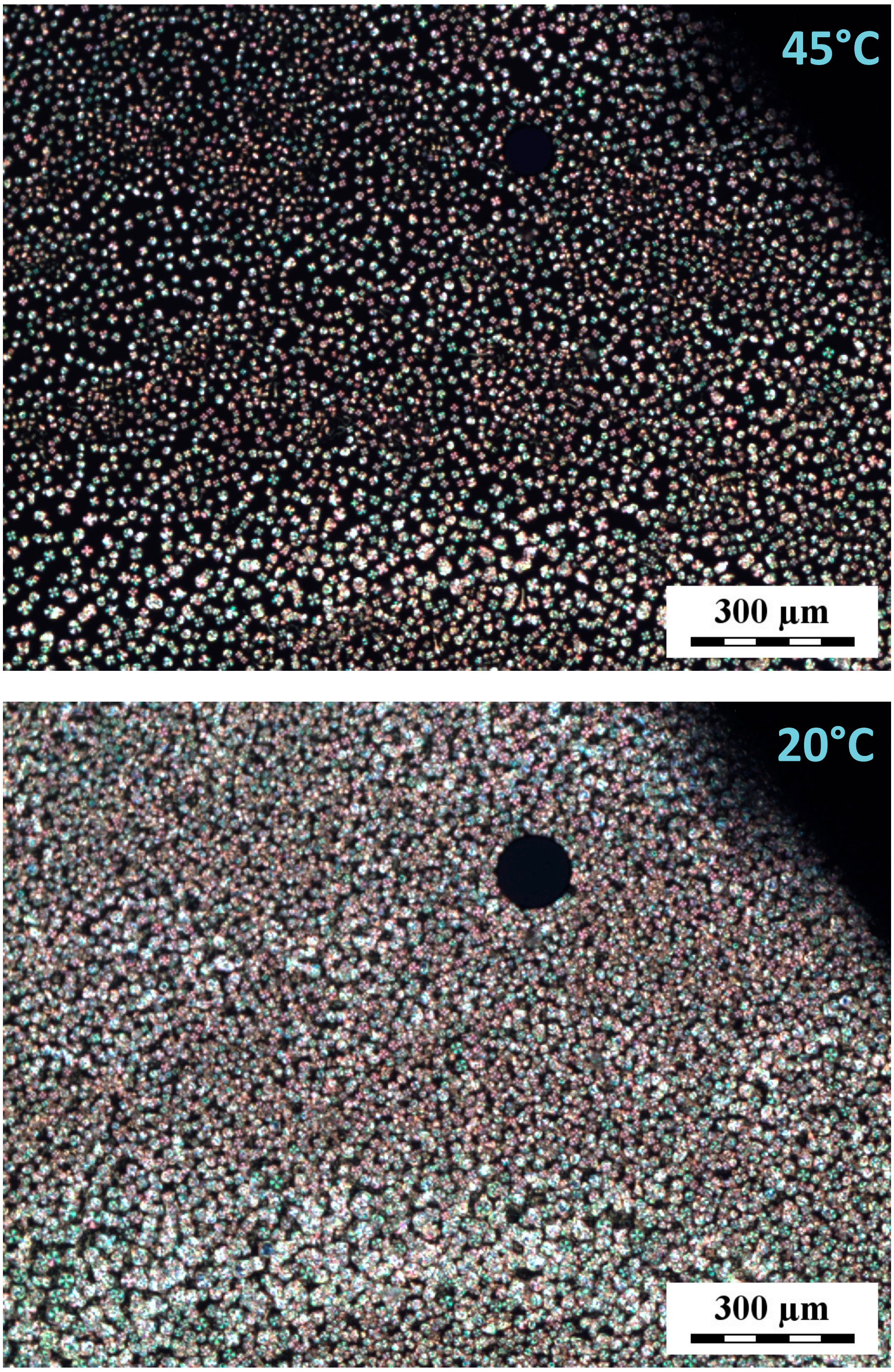

SI-Fig. 24: *High-resolution images of textures of* ***H03–BAFKU$_2$****: top: 45°C, bottom: 20°C, both in cooling run.*

1.4.3. Details of PLM textures: H11–BAFKU$_2$

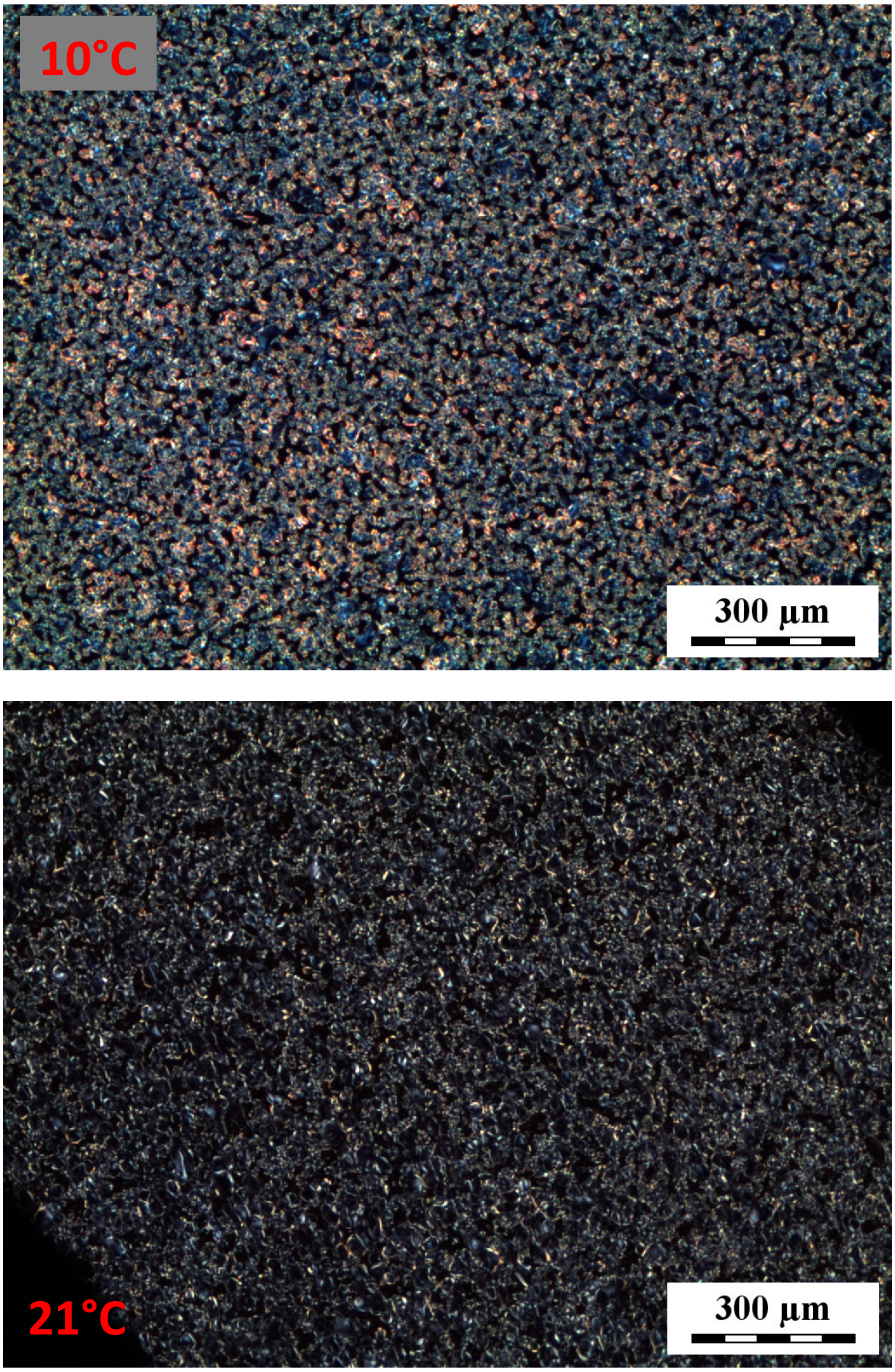

SI-Fig. 25: *High-resolution images of textures of* ***H11–BAFKU$_2$****: top: 10°C, bottom: 21°C, both in heating run.*

H11–BAFKU$_2$

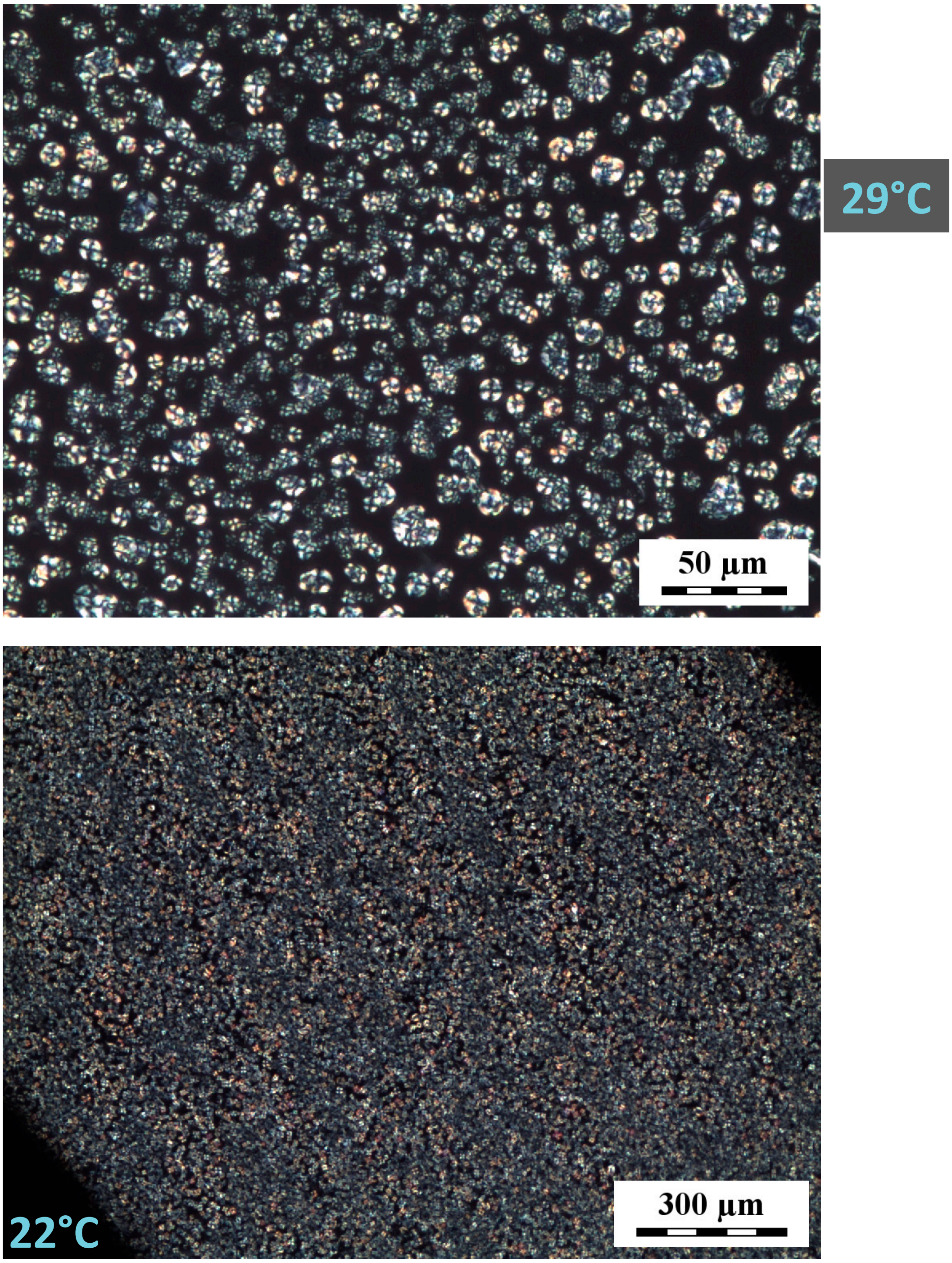

SI-Fig. 26: *High-resolution images of textures of* ***H11–BAFKU$_2$****: top: high detail at 29°C, bottom: 22°C, both in cooling run.*

H11–BAFKU$_2$

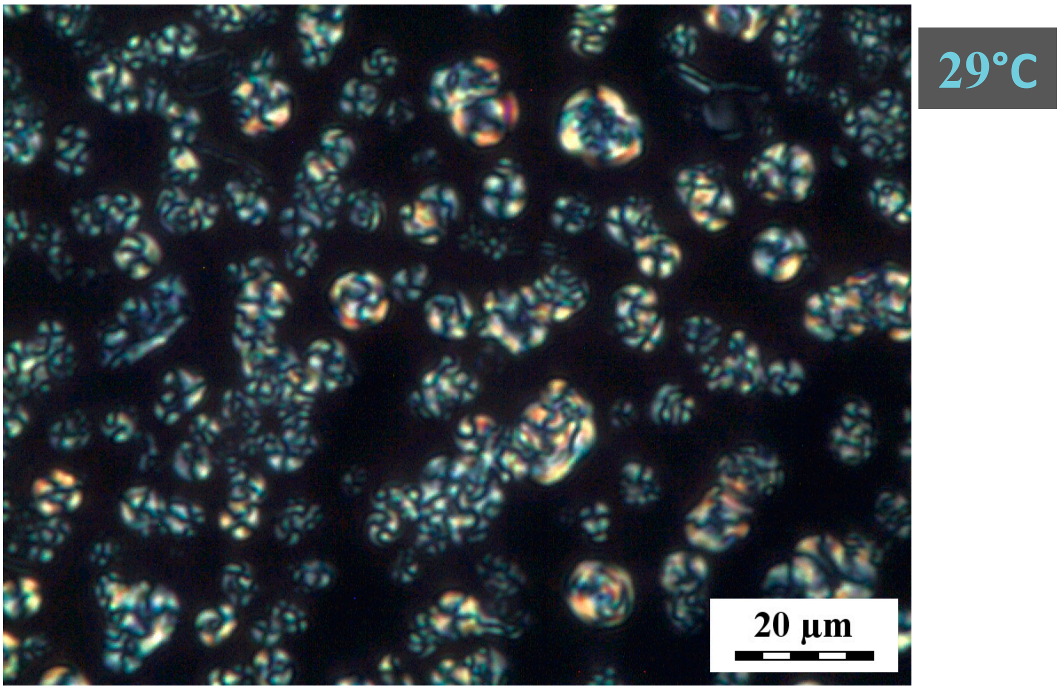

SI-Fig. 27: *High-resolution images of textures of* ***H11–BAFKU$_2$****: very high detail at 29°C.*

1.4.4. Details of PLM textures: H21–BAFKU$_2$

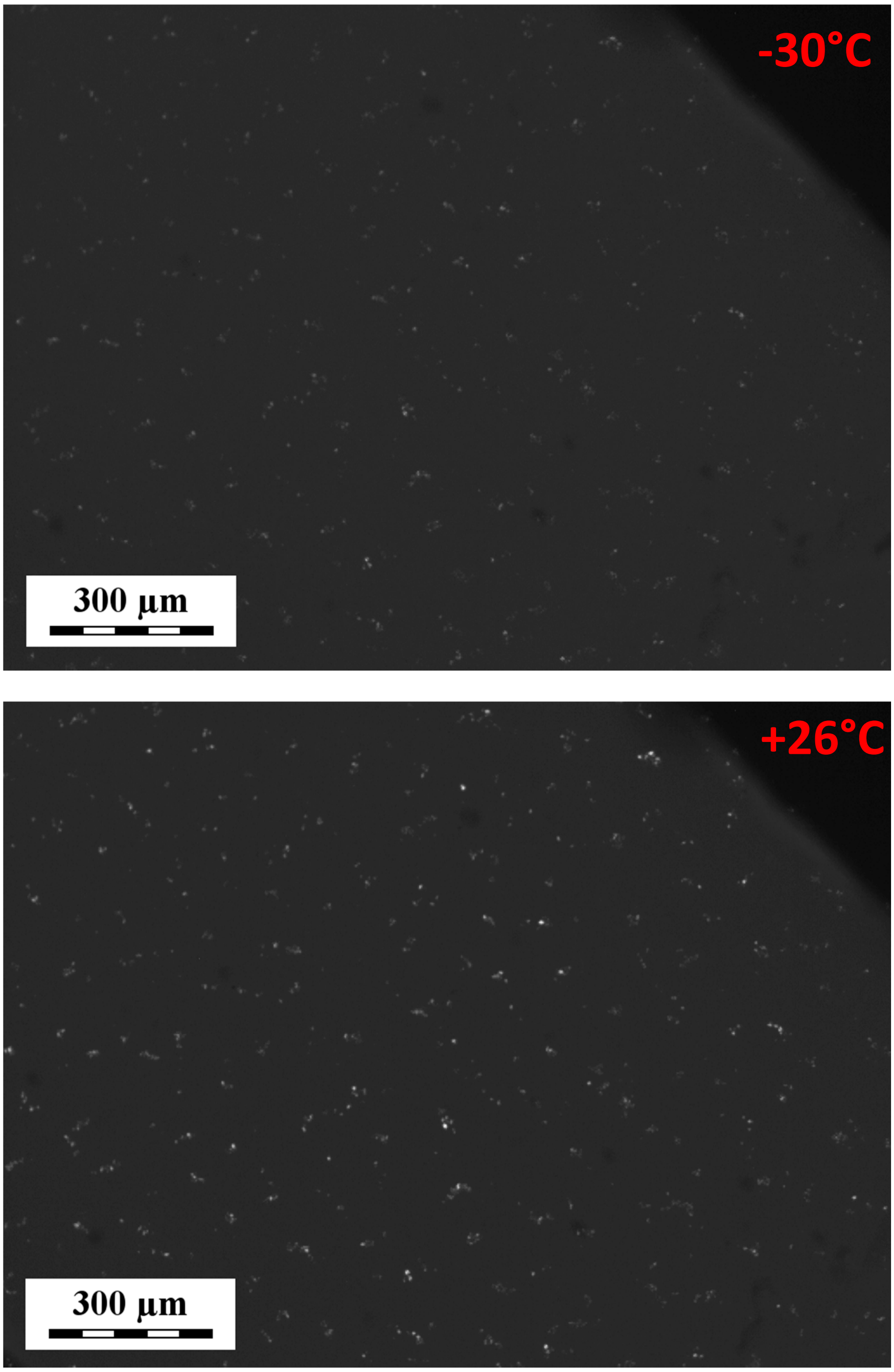

SI-Fig. 28: *High-resolution images of textures of **H21–BAFKU$_2$**: top: 10°C, bottom: 21°C, both in heating run.*

H21–BAFKU$_2$

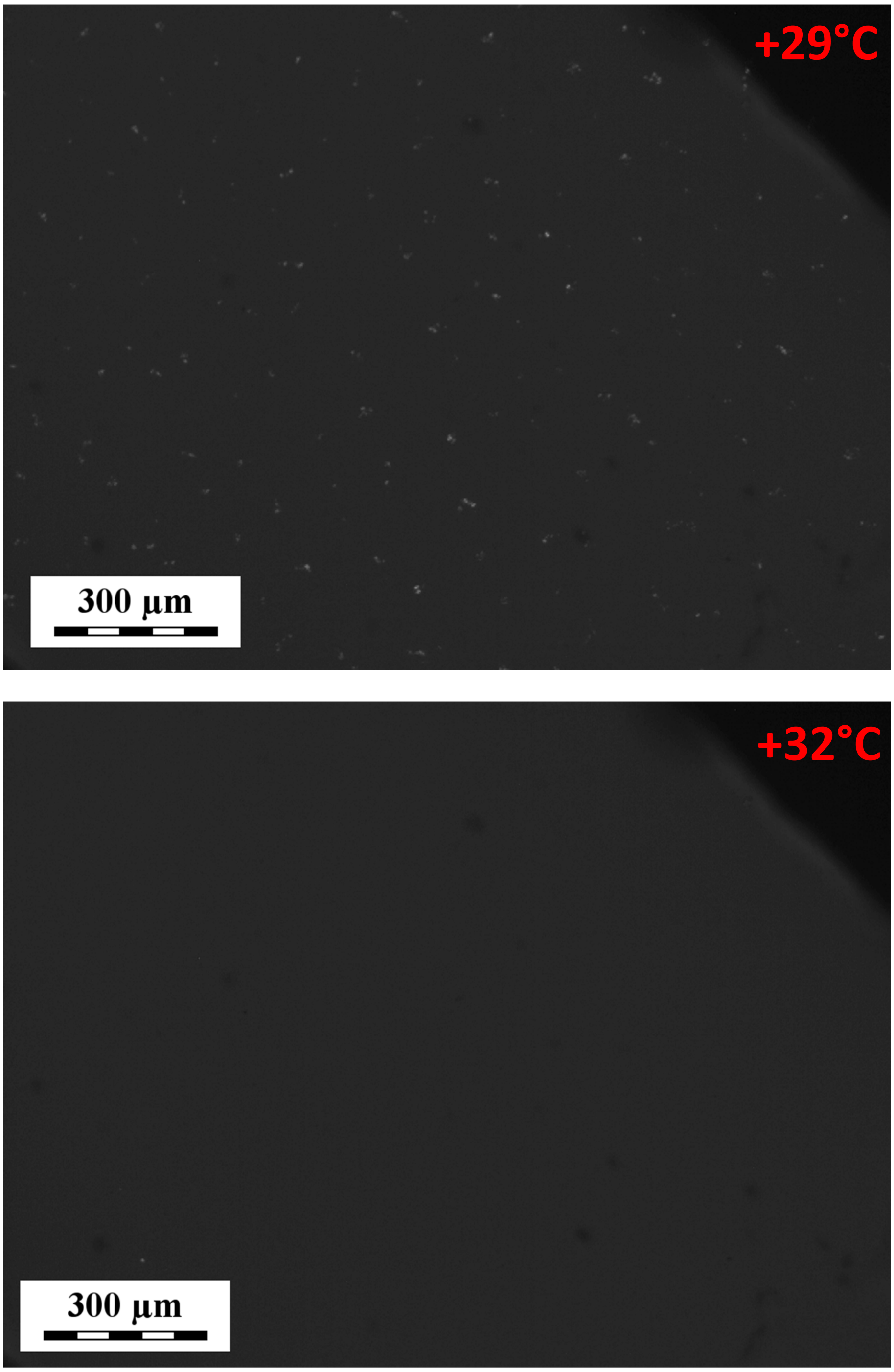

SI-Fig. 29: *High-resolution images of textures of* ***H21–BAFKU$_2$****: top: 10°C, bottom: 21°C, both in heating run.*

H21–BAFKU$_2$

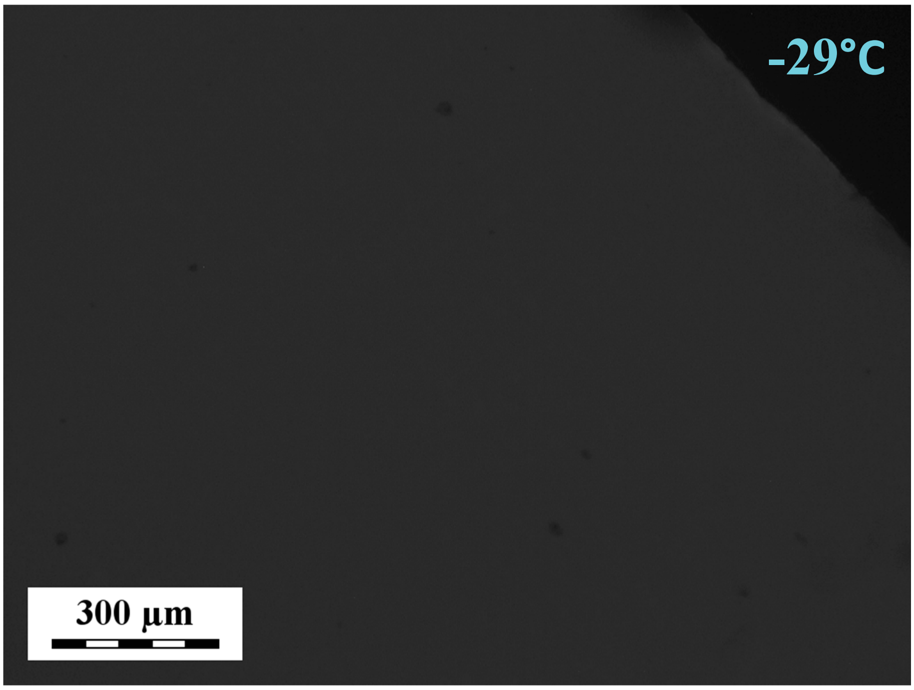

SI-Fig. 30: *High-resolution images of textures of* ***H21–BAFKU$_2$****: -29°C, end of cooling run.*

References

[1] S. Horodecka, A. Strachota, B. Mossety-Leszczak, M. Šlouf, A. Zhigunov, M. Vyroubalová, D. Kaňková, M. Netopilík, Meltable copolymeric elastomers based on polydimethylsiloxane with multiplets of pendant liquid-crystalline groups as physical crosslinker: A self-healing structural material with a potential for smart applications. *European Polymer Journal* 137 (2020) #109962. DOI: https://doi.org/10.1016/j.eurpolymj.2020.109962
